# Pervasive phosphorylation by phage T7 kinase disarms bacterial defences

**DOI:** 10.1038/s41586-026-10934-5

**Published:** 2026-08-19

**Authors:** Tara Bartolec, Karin Mitosch, Clément Potel, Federico Corona, Alessio Ling Jie Yang, Nicolai Karcher, Mira Lea Burtscher, Alexandra Koumoutsi, Isabelle Becher, Lena Sarah Müller, Jacob Bobonis, Manjeet Kumar, Marco Galardini, Athanasios Typas, Mikhail M. Savitski

**Affiliations:** 1 Molecular Systems Biology Unit, European Molecular Biology Laboratory, Heidelberg, Germany; 2 Genome Biology Unit, European Molecular Biology Laboratory, Heidelberg, Germany; 3 Collaboration for joint PhD degree between EMBL and Heidelberg University, Faculty of Biosciences, Heidelberg, Germany; 4 Microbial Automation & Culturomics Core Facility, European Molecular Biology Laboratory, Heidelberg, Germany; 5 Florida Research and Innovation Center, Cleveland Clinic, Port St Lucie, FL USA; 6 Cluster of Excellence RESIST (EXC 2155), Hannover Medical School (MHH), Hannover, Germany; 7 Present Address: Division of Microbial Ecology, Centre for Microbiology and Environmental Systems Science, University of Vienna, Vienna, Austria; 8 Present Address: Institute for Molecular Bacteriology, TWINCORE Centre for Experimental and Clinical Infection Research, Hannover, Germany

**Keywords:** Phage biology, Proteomics, Phosphorylation, Bacteriophages

## Abstract

Bacteria and bacteriophages are in a constant arms race to develop defence and anti-defence systems, respectively. Currently known phage-encoded anti-defence systems are specific to the activity of the targeted bacterial defence system. Here we identify a mechanism by which the T7 bacteriophage broadly counteracts bacterial defences using protein phosphorylation. Its kinase (T7K), which has been reported to redirect the function of a few host proteins^1–5^, is actually a hyperpromiscuous dual-specificity kinase that phosphorylates nearly all host and phage proteins during infection. The scale of phosphorylation vastly exceeds known phosphosites in *Escherichia coli*, has no sequence motif specificity and results in a higher proteome-wide phosphorylation density than mammalian cells with around 500 kinases. Stoichiometry analysis of phosphorylation sites revealed strong bias in T7K activity towards nucleic-acid-binding substrates mediated by its C-terminal DNA-binding domain. This highly stoichiometric phosphorylation enables the deactivation of DNA-targeting or DNA-containing bacterial defence systems. We provide mechanistic insights into how T7K weakens DNA-containing Retron-Eco9 through specific phosphorylation events, with single phosphomimetic mutations in key sites of the toxin abolishing defence. Moreover, by screening a large collection of *E. coli* strains, we provide evidence of broad anti-defence abilities of T7K in nature, as counteracted strains contain diverse bacterial defence systems. T7K homologues are found almost exclusively in phages, with hyperpromiscuous kinase activity probably being enabled by a divergent DFG-like motif in the catalytic centre.

## Abstract (web summary)

The T7 bacteriophage deactivates bacterial defence systems through T7K phosphorylation of nearly all host and phage proteins during infection.

## Main

Bacteria and phages engage in an evolutionary arms race to overcome each other’s defences^6^. Our understanding of the bacterial immune repertoire and its mechanisms^7^ has increased considerably since 2018 from a few defence systems (mainly CRISPR and restriction-modification enzymes) to hundreds of new families^6,8^. Consistently, most of the phage-encoded anti-defence proteins are anti-CRISPR or anti-restriction-modification enzymes^9,10^. Only a few dozen anti-defence phage proteins against other systems have been recently identified. Most directly counteract the defence system^11^ by blocking its activity^12^ or sequestering its product^13^, while others repair the damage caused by the defence system^14^. In all cases, known phage-encoded anti-defence proteins counteract a single bacterial defence system, or more than one system when those use the same molecule^15^ or target the same host cellular machinery^14^.

Short reproduction cycles necessitate fast defence and anti-defence mechanisms^16^. Protein post-translational modifications, which are prominent in eukaryotic host–pathogen interfaces^17^, are emerging to be as important in phage–bacterial interactions. Acetylation deactivates host CRISPR defences^18^, ubiquitin-like modification interferes with phage assembly^19^ and ADP-ribosylation modulates host transcription^20^ and translation^21^. Protein phosphorylation is used in bacterial host defence^22^ and was recently implicated in phage-encoded anti-defence^23^.

**Fig. 1:**
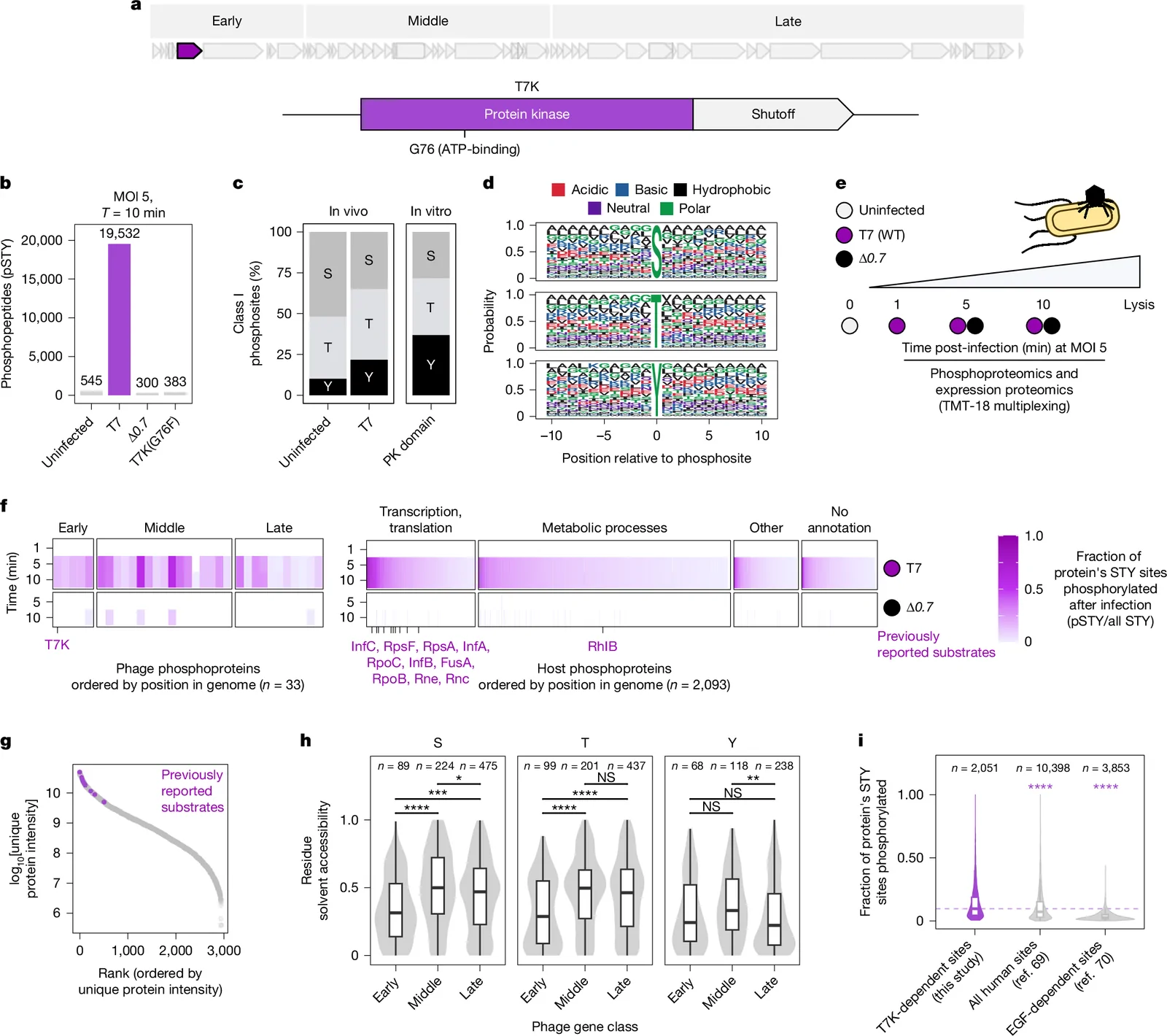
T7K hyperphosphorylates host and phage proteomes on Ser/Thr/Tyr residues. **a**, The phage T7 genome, with gene expression classes^41^. The domain architecture and ATP-binding site of the protein kinase T7K (encoded by *0.7*) are shown.** b**, Total unique Ser/Thr/Tyr phosphopeptides (*n* = 3 biological replicates; phosphoenrichment, label-free MS/MS) indicate massive phosphorylation during T7 infection, but not during infection with the Δ*0.7* or T7K(G76K) T7 mutants. **c**,**d**, T7K has dual specificity and no motif preference. **c**, The proportion of class I phosphosites (PTMProphet score ≥ 0.75) localized to Ser/Thr/Tyr residues in uninfected and T7-infected *E. coli* in vivo, or from an in vitro assay using purified recombinant T7K kinase domain (6×His-TEV-PK) on tryptic *E. coli* lysates.** d**, The corresponding sequence motif logo plot (10-mer window). **e**–**g**, Time-resolved quantitative (phospho)proteomics monitoring Δ*0.7* and WT T7 infections (*n* = 3 biological replicates) reveals rapid phosphorylation of most host and phage proteins by T7K. **e**, Experimental design.** f**, The fraction of significantly phosphorylated Ser/Thr/Tyr (pSTY) residues per protein (>2-fold, adjusted* P* < 0.05, limma versus uninfected; proteins with ≥1 phosphosite are shown, known substrates are indicated). Phage and host proteins are grouped by expression class and GO slim category, respectively.** g**, The intensities across time course show that known T7K substrates (purple) rank among the most abundant *E. coli* proteins.** h**, The residue solvent accessibility (RSA) of Ser/Thr/Tyr as determined using AlphaFold3 (1, exposed; 0, buried) reveals the depletion of accessible residues from early and middle phage protein surfaces. Statistical analysis was performed using unpaired two-sided *t*-tests. From left to right, *P* = 2.6 × 10^−6^, 0.00072, 0.014, 3.7 × 10^−6^, 3 × 10^−5^, 0.24, 0.11, 0.73, 0.0075. **i**, T7K targets relatively more proteins than all human kinases together. The fraction of phosphorylated Ser/Thr/Tyr residues per phosphoprotein was compared using two-sided unpaired two-sample Wilcoxon tests (*P* < 2 × 10^−16^ for both,* n* values indicate the number of phosphoproteins). T7K-dependent sites (purple) were significantly upregulated (>2-fold, adjusted *P* < 0.05, limma) in the WT versus uninfected and Δ*0.7* groups at matched timepoints. Human meta-analysis^69^ and an EGF-stimulated HEK293F^70^ dataset using the same phosphopeptide enrichment approach are included. The box plots in **h** and **i** show the median (centre line) and the interquartile range (IQR, 25th–75th percentiles; box limits) and the whiskers cover the smallest and largest values within 1.5 × IQR; outliers were omitted as they are present in the violin representation. *P ≤ 0.05, **P ≤ 0.01, ***P ≤ 0.001, ****P ≤ 0.0001; NS, not significant.

Bacteriophage T7 infects *E. coli* and encodes a serine–threonine protein kinase known as gp0.7 or T7K (encoded by *0.7*)^2,24^ that was identified in the 1970s^24^ (Fig. 1a). Known T7K substrates include the kinase itself^25^ and selected host proteins involved in transcription^1–3^, translation^26,27^ and nucleic acid processing^1,27,28^. The model is that T7K-mediated phosphorylation targets specific host proteins to hijack host cellular machineries for phage reproduction, shutting off or modulating host transcription^2–5^ and stabilizing phage mRNAs^1^. However, a clear understanding of the T7K function is lacking. The kinase is dispensable in standard growth conditions, with mild negative phenotypes in nutrient-poor medium and under heat stress^29^, and deactivates itself by phosphorylation early in the infection cycle. T7K is also needed during infection of *E. coli* armed with plasmids encoding the channel-forming colicin-1b toxin^30,31^. Finally, although the C-terminal shutoff (SO) domain of T7K is not required for kinase activity, it is highly toxic and capable of inhibiting host transcription independently of the kinase domain^24,32^.

Here we used modern phosphoproteomics to reinvestigate T7K activity and function. As opposed to the current model, we found that T7K acts as a hyperpromiscuous dual-specificity kinase that targets most phage and host proteins during a short burst of activity. This hyperactivity is conserved among *Autographiviridae* homologues. T7K gains preference towards nucleic-acid-binding proteins through its C-terminal domain, which we established binds to DNA, but probably not to RNA. Preferential targets get phosphorylated with high stoichiometry, which can lead to their deactivation. As many defence systems sense and/or target nucleic acids, we assessed the role of T7K during infection of *E. coli* armed with nucleic acid-binding defence systems. T7K-mediated phosphorylation at specific sites of Retron-Eco9 (ref. ^33^) and DarTG1 (ref. ^34^) abolished the ability of at least the former to defend against T7 infection. Finally, by screening a library of natural *E. coli* isolates, we found that T7K acts against a broad range of host defence systems, constituting a general phage-encoded anti-defence mechanism.

## T7K targets the entire *E. coli* proteome

**Extended Data Fig. 1:**
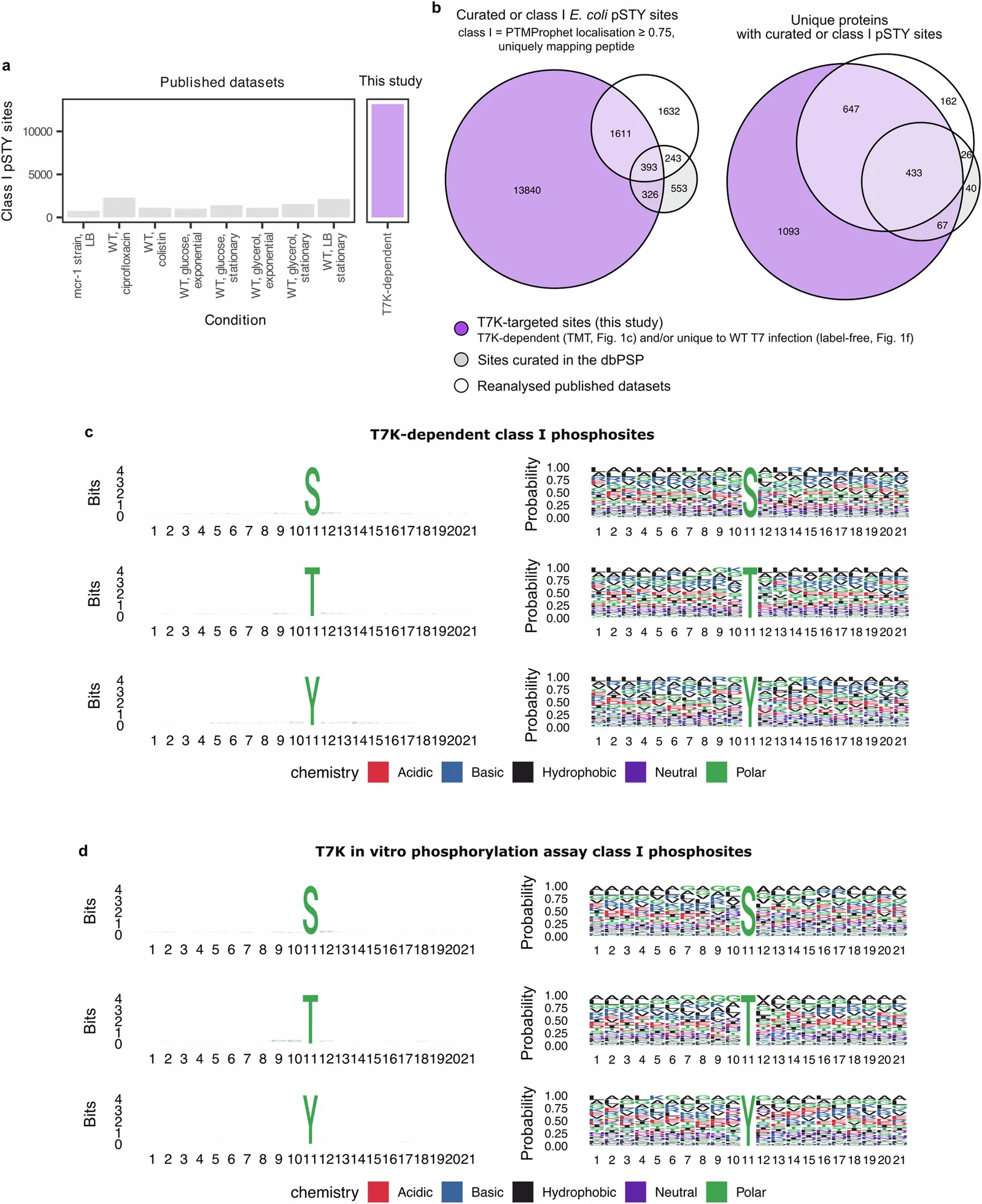
T7K is a hyper-promiscuous protein kinase. (**a**) The number of well-localized phosphosites on serine (S), threonine (T) and tyrosine (Y) residues that were T7K-dependent compared to several other published phosphoproteomic datasets of various cellular perturbations of *E. coli*. These included *E. coli* (i) grown in either glycerol or glucose at exponential or stationary phase^81^, (ii) grown in minimal media^120^, or (iii) under antibiotic stress, with and without genetic resistance to the colistin antibiotic (mcr-1 mutant strain)^121^. All datasets were re-analysed using the same MSFragger settings and FASTA database to facilitate comparison. T7K-dependent refers to sites from phosphopeptides significantly upregulated in wildtype T7 infection relative to uninfected, and also relative to the Δ*0.7* infection at a matched time-point (limma, FC > 2, adjusted p-value < 0.05). Class I sites have a high confidence of the modification’s localization within the peptide (PTMProphet^90^ localization score ≥ 0.75). (**b**) Overlap of phosphosites and phosphoproteins with relevant databases and the reanalyzed, previously published datasets. Class I phosphosites considered to be T7K-targeted were those that were host sites and were either (i) found uniquely in the wildtype T7 infection samples in label-free analysis described in Fig. 1b, or (ii) found to be T7K-dependent in the TMT time-course (see above). These sites and resulting phosphoproteins were compared to the curated *E. coli* sites in the bacterial phosphorylation database dbPSP^97^, and also to the bacterial mass-spectrometry based studies of the *E. coli* phosphoproteome under various perturbations which we reanalyzed as described above. (**c**) and (**d**) Phosphosite motif analysis. Motif analysis of phosphosites from class I phosphopeptides (PTMProphet localization ≥ 0.75, uniquely mapped to a single protein) from either (**c**) T7K-dependent phosphopeptides identified in the TMT time-course (Fig. 1e) or (**d**) from phosphopeptides identified in the in vitro phosphorylation assay using the purified T7K kinase domain (Fig. 1c).

Using a bacterial-adapted phosphoproteomics approach, we found that host and phage proteomes were hyperphosphorylated during T7 infection. We detected 19,532 phosphopeptides in wild-type (WT) T7 infection, compared with a few hundred in the uninfected and mutant controls, the T7K knockout (T7Δ*0.7::cmk*, hereafter Δ*0.7*) and the catalytic-dead G76F^35^ mutant (Fig. 1b and Supplementary Table 1), implying that T7K drives this massive phosphorylation. Detected phosphosites exceeded, by over threefold, the total number of reported sites in *E. coli* probed in different conditions (Extended Data Fig. 1a,b and Supplementary Table 1). As most phosphorylation occurred on unreported sites and proteins, we reasoned that T7K deposits these modifications. Phosphorylation occurred on serine (Ser), threonine (Thr) and tyrosine (Tyr) residues (Ser/Thr/Tyr; Fig. 1c), suggesting that T7K is a dual-specificity, highly promiscuous kinase. Structural predictions of T7K resembled human dual-specificity kinases like CLK1, CLK2 and DYRK1A, and the top PFAM match for its orthologous group was the dual-specificity kinase domain PF07714.16 (Supplementary Table 2).

To validate T7K’s dual specificity and high promiscuity, we performed in vitro phosphorylation of *E. coli* tryptic digests using the purified, dephosphorylated T7K kinase domain. We identified 12,642 phosphopeptides with Ser, Thr and Tyr phosphosites (Supplementary Table 1). Tyrosine phosphorylation was higher in vitro than in vivo (Fig. 1c), probably because Tyr residues are frequently buried in folded proteins^36^. Importantly, T7K recognized sites without sequence specificity (Fig. 1d and Extended Data Fig. 1c,d), confirming its promiscuity.

**Extended Data Fig. 2:**
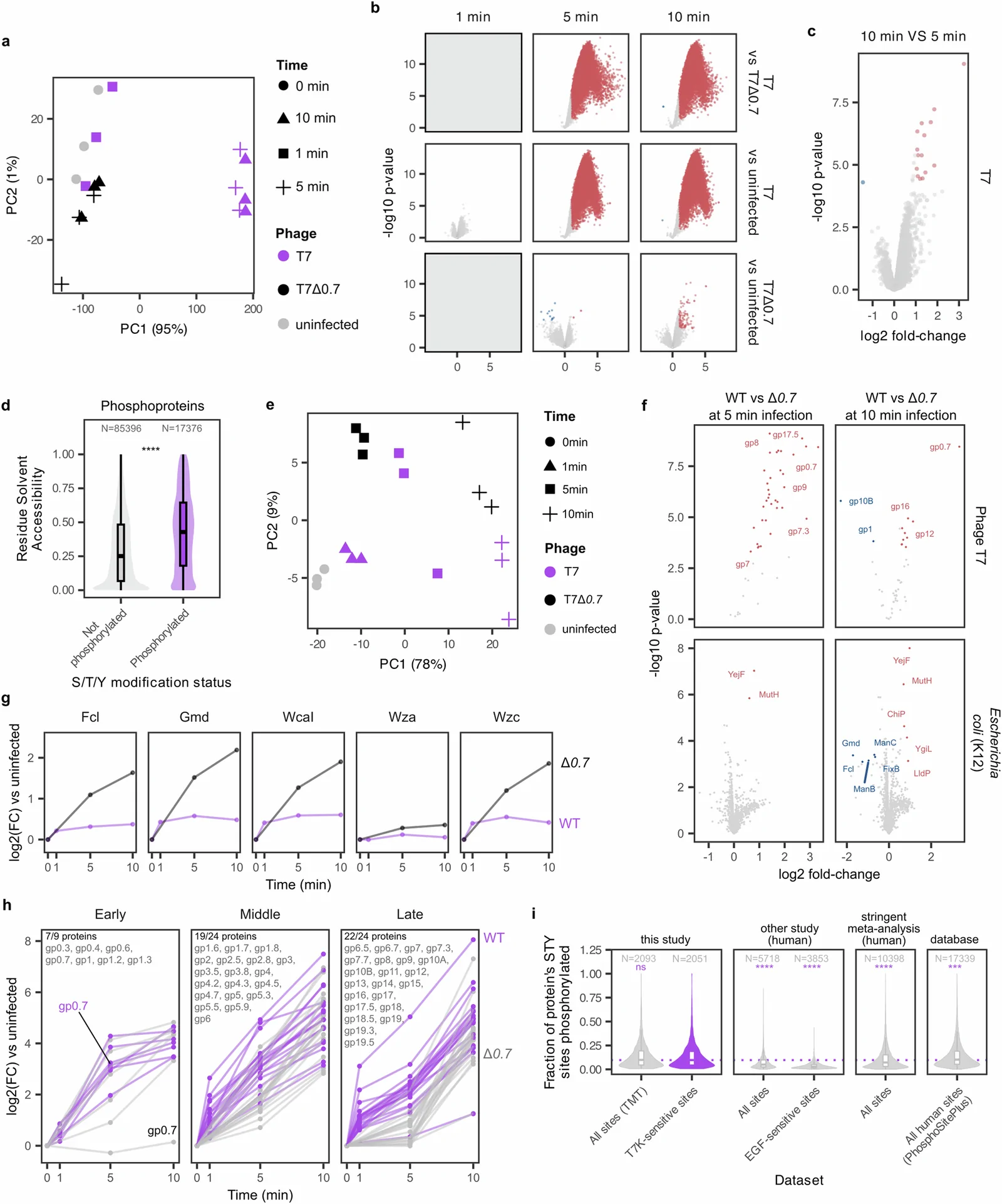
Analysis of T7 infection time-course phosphoproteomics and proteomics datasets. (**a**) Principal component analysis (showing top-2 principal components) of samples based on vsn-normalized^93^ phosphopeptides with full quantification in the TMT-multiplexed time-course analysis of wildtype T7 and Δ*0.7* infections, across three biological replicates (Fig. 1e). (**b**) Volcano plots showing the results of limma differential phosphopeptide abundance analysis for several comparisons of wildtype or Δ*0.7* infections to the uninfected sample, or to each other at matched time-points. Red and blue dots show phosphopeptides with significance (moderated unpaired two-sided t-test (limma), adjusted p-value < 0.05, 2-fold change) with upregulated sites as red and downregulated sites as blue. Grey facets show comparisons that were not possible (comparison against 1 min infections involving Δ*0.7*) due to the TMT-multiplexing design constraints. (**c**) Volcano plot showing an additional comparison between 5 and 10 min of wildtype T7 infection, illustrating minimal changes to the phosphorylation levels which likely reflects deactivation of the kinase activity by autophosphorylation. Red and blue dots show phosphopeptides with significance (moderated unpaired two-sided t-test (limma), adjusted p-value < 0.05, 2-fold change) with upregulated sites as red and downregulated sites as blue. (**d**) Residue surface accessibility of S/T/Y residues within host phosphoproteins (proteins found phosphorylated at at least one site in the TMT analysis) subsetted by their phosphorylation status in the TMT analysis. Note: non-unique peptides were included for this analysis. The distributions were compared with two-sided, unpaired t-tests (**** meaning p-value ≤ 0.0001, p < 2.2e-16). N indicates the number of residues considered in each distribution. (**e**) Principal component analysis (showing top-2 principal components) of samples based on vsn-normalized protein intensities with full quantification in the TMT-multiplexed time-course analysis of wildtype T7 and Δ*0.7* infections, across three biological replicates (Fig. 1e). (**f**) Volcano plots showing the results of limma differential protein abundance analysis for wildtype T7 infection compared to Δ*0.7* infection at 5 min and 10 min. Red and blue dots show proteins with significance (moderated unpaired two-sided t-test (limma), adjusted p-value < 0.05, 1.5-fold change) with upregulated sites as red and downregulated sites as blue, and proteins are grouped by their organismal source. N = 48 phage proteins for the top facet, and N = 2,919 host proteins in the bottom facet for each volcano plot. (**g**) Selected time-course measurements (log2FC of infected sample at given time-point vs uninfected) for host proteins regulated within the *wza* operon which is involved in colanic acid synthesis. Purple profiles show the protein’s expression pattern upon infection with wildtype T7 and black in Δ*0.7* infection. (**h**) Time-course measurements for phage T7 proteins classified by T7 gene class. The profiles for T7K (gp0.7) are indicated. The top left corner of each sub-graph shows the numbers and (gene product) names of phage proteins within each category that were successfully quantitatively monitored. (**i**) Extension of Fig. 1i, showing the per-phosphoprotein (S/T/Y) phosphorylation fraction across several sources of information on the human phosphoproteome. T7K-dependent sites (purple) are the same as described above. All sites (TMT) refers to all phosphosites identified and quantified in the TMT experiment (regardless of T7K-dependency). “other study (human)” refers to a recent phosphoproteomics study from our group investigating phosphoregulation in human HEK293F cells after EGF stimulation^70^, which used the same phosphoenrichment strategy. “All sites” refers to all identified and quantified sites in this study, whereas “EGF-sensitive sites” are those reported by the paper to be significantly induced by EGF stimulation, which is known to rapidly induce a wave of downstream phosphosignalling in the human cell. “stringent meta-analysis (human)” refers to all phosphosites reported in a recent meta-analysis and curation of the human phosphoproteome^69^, and “database” refers to all human sites listed on the PhosphoSitePlus database^98^. The distributions of the different sets were compared to the T7K-dependent set using unpaired two-sided two-sample Wilcoxon signed-rank tests (**** meaning p-value ≤ 0.0001, *** meaning p-value ≤ 0.001; from left to right p = 0.49108, <2e-16, <2e-16, <2e-16, 0.00047). Numbers indicate the number of phosphoproteins considered in each distribution. The boxplot shows the data distribution, with the box spanning the interquartile range (25th and 75th percentile) and the median indicated at the line. Whiskers extend to the smallest and largest values within 1.5 × IQR, while outliers are not plotted as they are encompassed by the corresponding violin plot.

To study T7K dynamics, we measured phosphopeptide and protein abundance in *E. coli* K-12 during WT and Δ*0.7* T7 phage infections. As the T7 infection cycle is rapid (around 17 min to cell lysis) and because the kinase autophosphorylates and loses activity within 6 min^25,37^, we sampled before infection and at 1, 5 and 10 min after infection (Fig. 1e,f and Extended Data Fig. 2). Although no upregulated phosphopeptides were detected at 1 min, consistent with minimal T7K and phage protein expression, massive phosphorylation was detected within 5 min (Extended Data Fig. 2a–c), yielding 15,399 phosphopeptides that uniquely mapped to 2,093 host and 33 phage proteins (Fig. 1f and Supplementary Table 3). Most unique phosphopeptides (14,663) were T7K dependent, targeting around 70% of the expressed host and phage proteomes (Supplementary Table 3). Many host proteins exhibited high phosphorylation densities (fraction of detectable Ser/Thr/Tyr residues modified), showcasing the processivity and promiscuity of T7K (Fig. 1f). This includes all 11 known substrates^1–3,25–27^, which were among the most abundant proteins in the cell (Fig. 1g). Consistent with T7K acting in the cytoplasm, cell envelope and membrane proteins were depleted of phosphorylation (Supplementary Table 3). Modified host sites were significantly highly surface accessible (Extended Data Fig. 2d), indicating that T7K acts predominantly on mature, folded proteins. In contrast to extensive host protein phosphorylation, only ten host proteins significantly changed in abundance in the absence of the kinase (Extended Data Fig. 2e,f). This included downregulation of members of the colanic acid biosynthesis pathway^38^, which protects against phage infection^39,40^ (Extended Data Fig. 2g).

In terms of the T7 phage, late proteins had lower phosphorylation densities compared with early or middle proteins^41^ (Fig. 1f), reflecting temporal kinase autoinhibition^25,37^. Although early T7 proteins were heavily phosphorylated, their sequences have been selected for significantly fewer surface-accessible Ser/Thr residues (Fig. 1h), highlighting the evolutionary pressure of T7K hyperpromiscuity on the T7 genome. Tyr residues exhibited low surface accessibility for all phage proteins. Consistent with early T7K shutdown, only 15 unique phosphopeptides increased significantly between 5 and 10 min after infection (Extended Data Fig. 2c). Beyond the known Ser227 site^25^, we quantified additional phosphosites on T7K (Tyr122, Tyr150 and Thr346), but could not confidently quantify the inhibitory Ser216 site (Supplementary Table 3). In contrast to protein phosphorylation, phage protein abundance increased between 5 and 10 min, confirming infection progression (Extended Data Fig. 2h and Supplementary Table 3). Consistent with slightly slower infection kinetics reported for the kinase-deficient T7 (ref. ^29^), infection with Δ*0.7* T7 phages yielded lower middle and late protein abundances, particularly at 5 min (Extended Data Fig. 2h).

Together, we identified a wave of host and phage proteome phosphorylation within the first 5 min of T7 infection, driven by its dual-specificity T7K. This single kinase’s activity exceeds the collective reported output of the human kinome (more than 500) over the years^42^ (Fig. 1i and Extended Data Fig. 2i).

## T7K prefers DNA-binding substrates

**Fig. 2:**
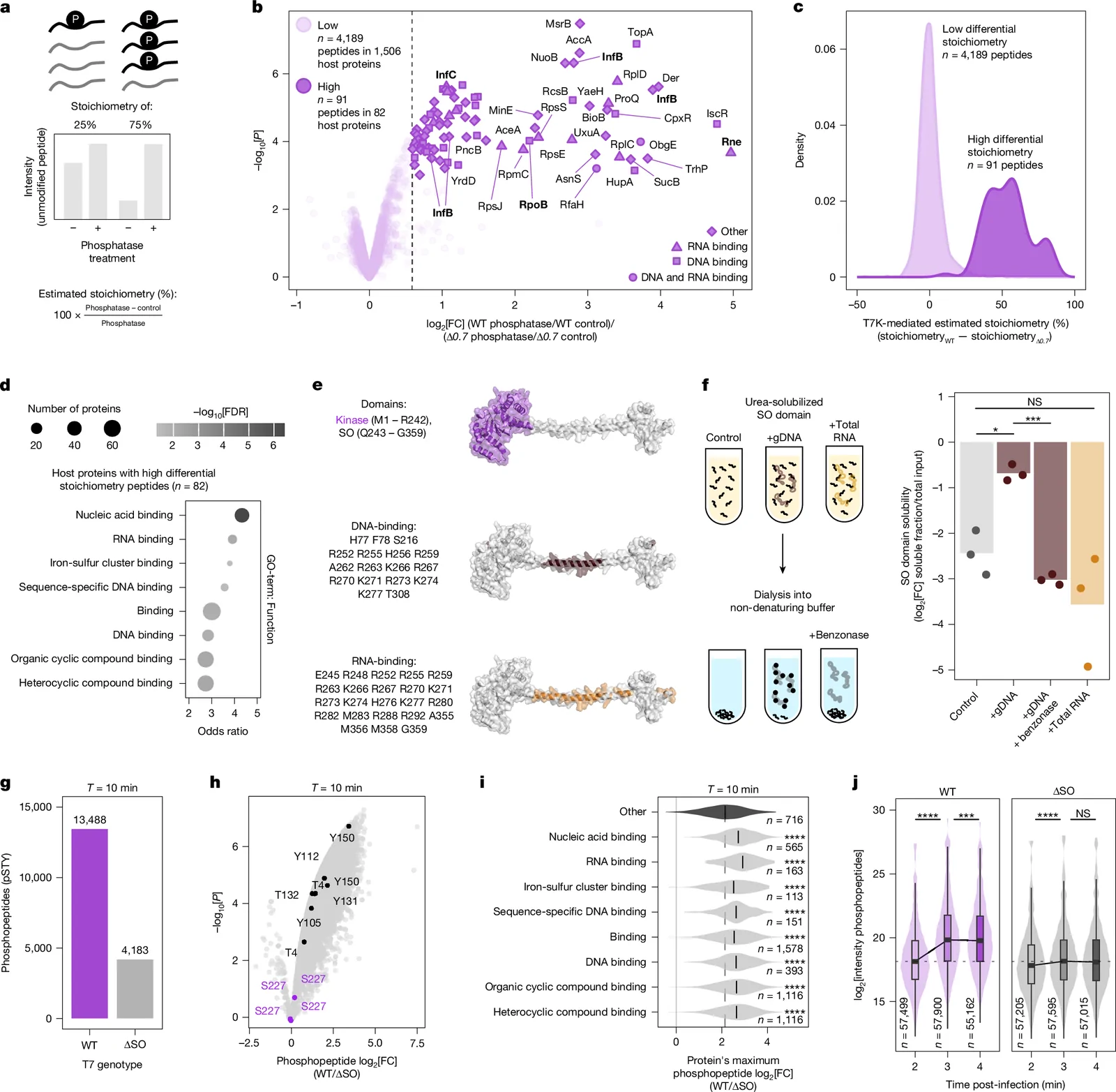
T7K hyperactivity targets DNA-binding proteins. **a**, The experimental workflow for estimating phosphorylation-site stoichiometry. **b**–**d**, Peptide phosphatase treatment in WT versus Δ*0.7* infected cells (*T* = 10 min, MOI = 5, *n* = 3 biological replicates) reveals high phosphorylation stoichiometry for specific host proteins. **b**, Volcano plots. **c**, Stoichiometry distributions. Only unique host peptides representing phosphosites identified in the time course (Fig. 1e,f) and with robust phosphatase/control ratios (Extended Data Fig. 3a) were assessed. Dark and light purple shading indicates peptides with a high (moderated unpaired two-sided *t*-test (limma), adjusted *P* < 0.05, fold change (FC) > 1.5) or low differential stoichiometry, respectively. The labels in **b** show proteins with high differential stoichiometry. Bold font indicates known substrates; the shapes represent annotation. **d**, GO-term enrichment of high-stoichiometry substrates from **b** (odds ratios > 1; >10 proteins), using unique host proteins as background. **e**, AlphaFold3 full-length T7K prediction using GraphBind identified a DNA/RNA-binding helix within the SO domain. **f**, The T7K SO domain binds to DNA but not to RNA. Urea-solubilized recombinant SO folded only with gDNA. Relative SO-domain solubility during in vitro refolding measured using TMT-based MS. *n* = 3 biological replicates. Statistical analysis was performed using two-sided unpaired *t*-tests; from top to bottom, *P* = 0.25, 0.00013, 0.016. **g**,**h**, Loss of the SO domain reduces T7K-mediated hyperphosphorylation, but not in autophosphorylation sites. **g**, Total unique Ser/Thr/Tyr phosphopeptides from WT and ΔSO infections (MOI = 5, *T* = 10 min, *n* = 3 biological replicates) as determined using label-free MS/MS. **h**, Limma analysis (moderated unpaired two-sided *t*-test, unadjusted *P* values) of TMT phosphoproteomics comparing phosphopeptides in WT and ΔSO infections at 10 min. *n* = 3 biological replicates. Ser227 is a previously reported T7K autophosphorylation site, with other detected T7K sites labelled. **i**,**j**, Loss of the SO domain accelerates inactivation of T7K activity. **i**, Maximum phosphopeptide log_2_[FC] per protein across GO terms (the cross bars indicate the median values; the dotted line indicates the median of ‘other’ proteins). Two-sided unpaired *t*-tests against other; all *P* < 2 × 10^−16^. **j**, Summed TMT reporter ion intensities (per phosphopeptide) monitoring WT and ΔSO kinetics. *n* = 3 biological replicates. The dotted line shows the median log_2_[summed intensity] at 2 min of WT infection. Statistical analysis was performed using two-sided unpaired *t*-tests; from left to right, *P* < 2 × 10^−16^, *P* = 2.51 × 10^−4^, *P* < 2 × 10^−16^, *P* = 0.458. The box plots and significance are as defined in Fig. 1h.

**Extended Data Fig. 3:**
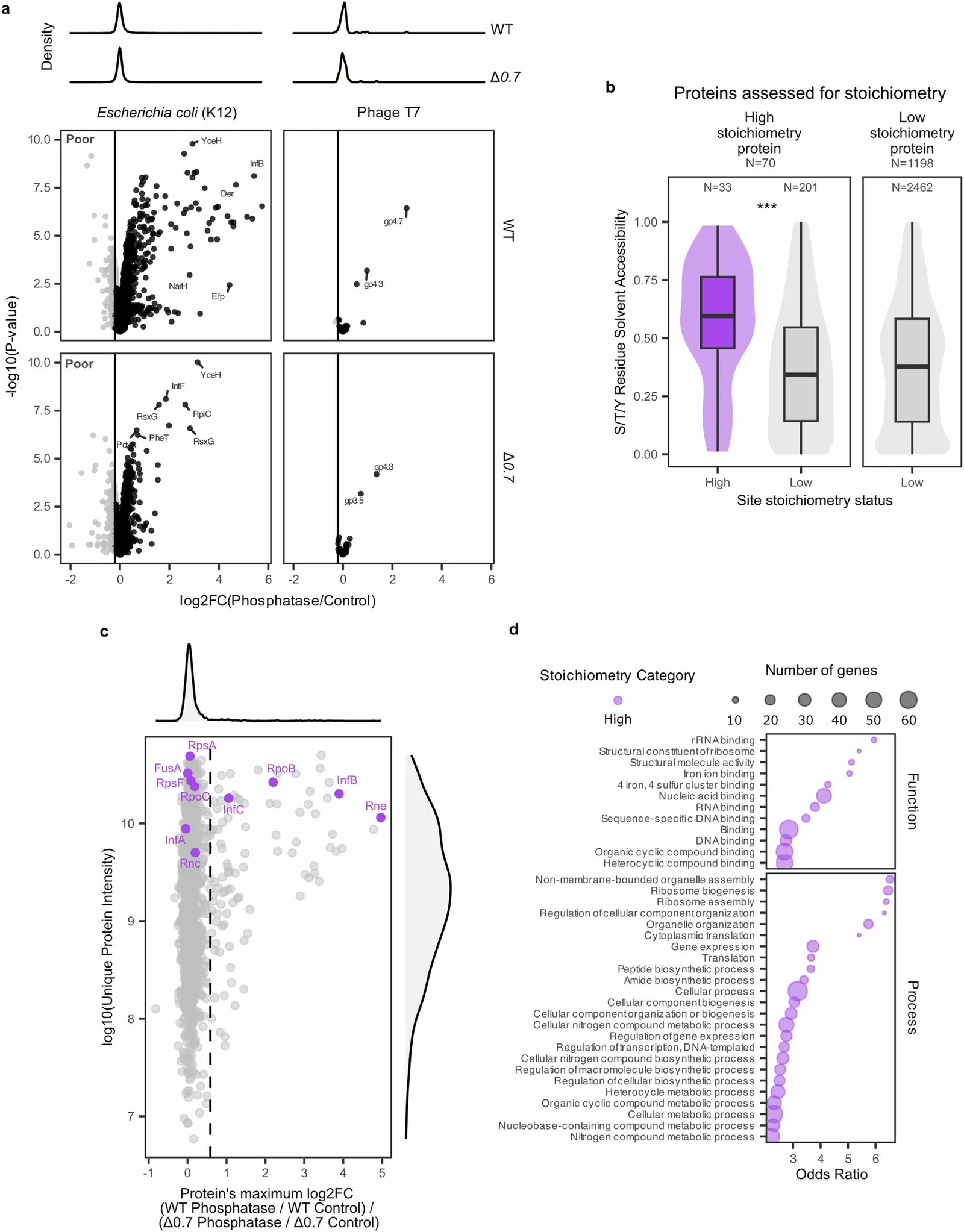
Estimating phosphorylation stoichiometries. (**a**) The Phosphatase/Control ratios of peptides across host and phage proteins considered for the estimation of phosphorylation stoichiometries (N = 4,371 peptides). These were (non-phosphorylated) peptides with residues previously identified to be phosphorylated in the TMT time-course phosphoproteomics dataset (Fig. 1e). Stoichiometries were estimated using three biological replicate samples of the T = 10 min infection time-points, and as such only phosphosites on peptides representing proteins that were not found to significantly differ between WT T7 and Δ*0.7* infection at 10 min in the protein expression analysis (Extended Data Fig. 4b) were considered. We excluded “Poor” Phosphatase/Control ratios (grey dots) from downstream differential stoichiometry analyses using limma (Fig. 2b) when the Phosphatase/Control ratio was either significantly down-regulated (moderated unpaired two-sided t-test (limma), adjusted p-value < 0.05, log2 fold-change (FC) < 0) or the log2FC was low (logFC < −0.2, no significance requirement). The log2FC = −0.2 position is indicated. This represented the minority of ratios (see corresponding density histogram for each sample, top of the graph), but removed peptides that had clear evidence of confounding technical factors (as the addition of phosphatase should not result in a decrease in signal, in theory). Both ratios were categorized as poor if they were found to be poor in at least one of the conditions (wildtype T7 or Δ*0.7*). Several peptides are labelled with the name of the protein they belong to, namely those that would be considered significantly high stoichiometries within their respective samples (adjusted p-value < 0.05, fold-changes (FC) > 1.5). (**b**) High stoichiometry sites are more surface accessible within protein structures. The residue surface accessibility values for S/T/Y residues were calculated from AlphaFold predictions for phosphoproteins that had quality phosphatase ratios calculated and used in Fig. 2b. Only proteins with peptide sequences distinctly encompassing a single phosphosite were considered. Distributions were compared using a two-sided, unpaired t-test, with *** meaning p-value ≤ 0.001 (p = 0.00045). N indicates the number of residues considered in each distribution. Boxplots show the data distribution, with the box spanning the interquartile range (25th and 75th percentile) and the median indicated at the line. Whiskers extend to the smallest and largest values within 1.5 × IQR, while outliers are not plotted as they are encompassed by the corresponding violin plot. (**c**) The maximum differential stoichiometry log2FC values for each protein compared to its abundance (unique intensity) (N = 1,538 proteins), where maximum refers to the highest log2FC obtained for any phosphopeptide mapping to the protein with quality ratios calculated from (a). Previously reported host substrates are indicated in purple and labelled with their protein names. (**d**) Full results of GO-term analysis on high stoichiometry phosphoproteins, which includes the data presented in Fig. 2d. Enrichment analysis was performed on *E. coli* proteins with peptides showing evidence of having **“**high” differential stoichiometry (see Fig. 2b). All GO-terms have a false discovery rate ≤ 0.05 (Benjamini-Hochberg corrected, using the STRING-db R package^96^), and the expressed *E. coli* proteome was used as background (all proteins identified by unique peptides in the proteomic experiment monitoring wildtype T7 and Δ*0.7* infections).

Phosphorylation regulates signalling and protein fate, but completely altering or abolishing protein function requires near-stoichiometric modification. Given T7K’s hyperpromiscuity, we investigated whether any site reached stoichiometric levels. Measuring stoichiometry is difficult because ionization efficiencies differ between modified and unmodified peptides. To circumvent this, we treated samples with phosphatase so that the relative intensity increase of unmodified peptides could capture the initial phosphorylation occupancy (Fig. 2a). Comparing unmodified peptide intensities from T7 WT and Δ*0.7* infections (10 min post-infection) yielded robust phosphatase/control ratios for 4,355 peptides, of which 4,280 represented host proteins (Extended Data Fig. 3a and Supplementary Table 4). Peptides with significantly increased phosphatase/control ratios in the WT over Δ*0.7* T7 infections (Fig. 2b) revealed phosphorylation with high stoichiometry of 82 host proteins, including 4 out of 11 known substrates, at accessible residues (Extended Data Fig. 3b and Supplementary Table 4). Low-abundance peptides are harder to measure, introducing false negatives (Extended Data Fig. 3c). Converting these ratios to stoichiometry values confirmed highly stoichiometric phosphorylation events (Fig. 2c), and Gene Ontology (GO)-term enrichment analysis identified specific classes involved in nucleic acid, DNA and (r)RNA binding (Fig. 2d and Extended Data Fig. 3d). Most previously reported T7K substrates are (r)RNA binding, with the only known DNA-binding substrates being RNA polymerase subunits. Among the highly stoichiometrically phosphorylated transcription factors was RcsB, which activates colanic acid biosynthesis and influences biofilm production^38,43^. This aligns with host protein abundance changes (Extended Data Fig. 2g) and T7K-mediated silencing of this bacterial defence against phage infection^39,40^.

**Extended Data Fig. 4:**
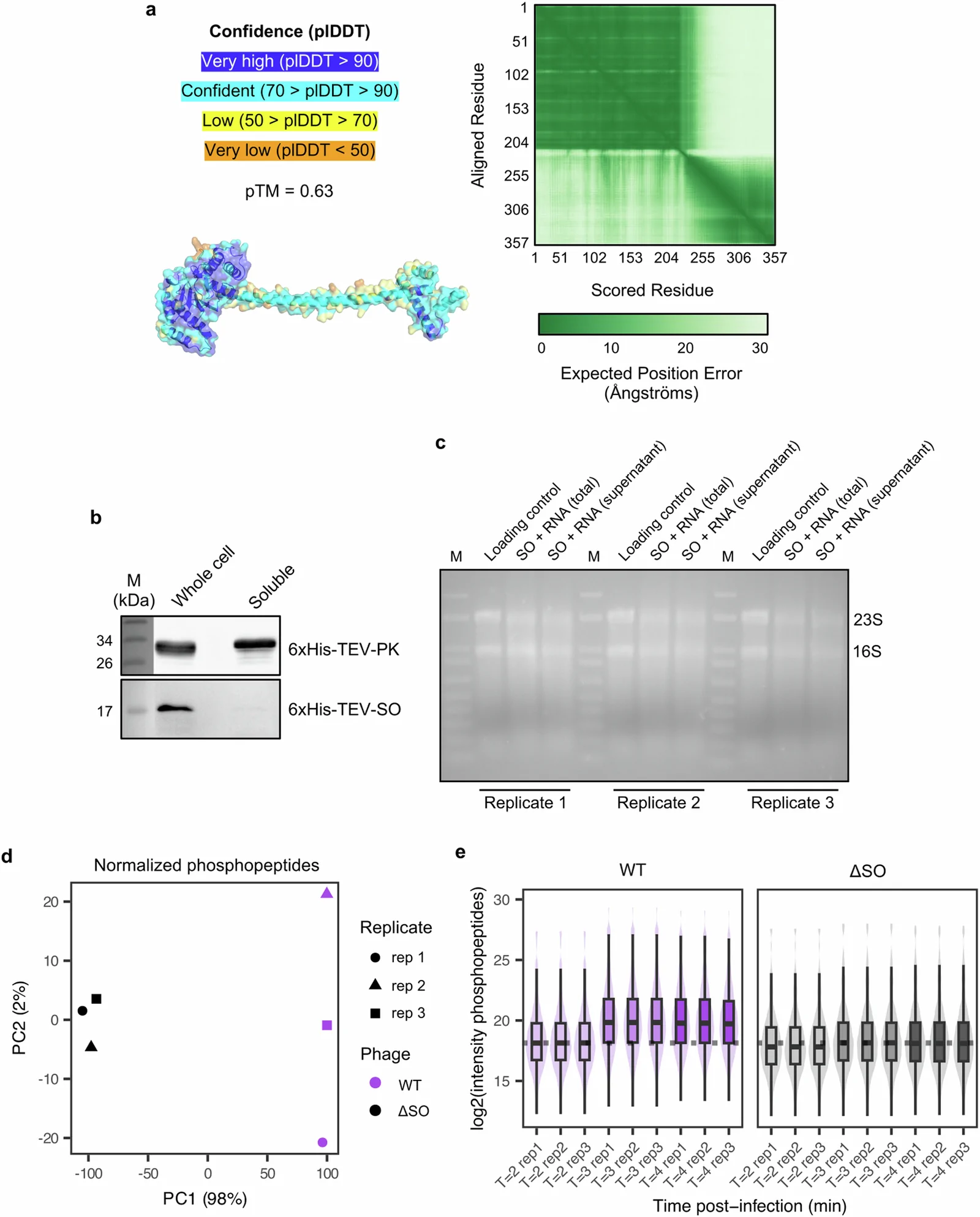
AlphaFold structural prediction, purification of T7K domains with refolding assay controls, and ΔSO infections. (**a**) Prediction confidence scores for the top-ranked AlphaFold3^105^ structural prediction of T7K. Residues are coloured by their residue-level predicted local distance difference test (plDDT) score confidence category, with the structure illustrated from N- to C-terminus, matching the depictions of T7K in Fig. 2e. The residue-residue alignment confidence (PAE) matrix and predicted template modelling (pTM) score are also shown. (**b**) Western Blot analysis of 6xHis-TEV-PK and 6xHis-TEV-SO before and after cell fractionation. Proteins were expressed in *E. coli* BL21-AI cells as 6xHis-TEV-PK and 6xHis-TEV-SO. Whole cell lysates or soluble cell extracts were loaded on 15% polyacrylamide and proteins resolved by SDS-PAGE, then transferred to PVDF. Membranes were probed with anti-His antibodies (Invitrogen) to detect proteins of interest. One of two independent replicates is shown. (**c**) Assessment of RNA integrity after overnight dialysis during in vitro refolding of 6xHis-TEV-SO. Samples from refolding reactions (total and supernatant) in the presence of RNA were analysed by electrophoresis on 1.5% agarose and ethidium bromide staining. Equivalent amounts of the RNA stock sample were loaded as controls. RNA integrity was assessed by comparing 23S and 16S bands from refolding reactions to controls. M, molecular weight marker. (**d**) Principal component analysis (showing top-2 principal components) of samples based on vsn-normalized^93^ phosphopeptides with full quantification in the TMT-multiplexed phosphoproteomics dataset comparing wildtype T7 and ΔSO T = 10 min infections across three biological replicates (Fig. 2h). (**e**) Distributions of individual biological replicates (plotted combined in Fig. 2j) of summed TMT reporter ion intensities per phosphopeptide derived from a quantitative TMT-based phosphoproteomics monitoring 2, 3 and 4 min of WT and ΔSO infections. N = 3 independent biological replicates. Boxplots show the data distribution, with the box spanning the interquartile range (25th and 75th percentile) and the median indicated at the line. Whiskers extend to the smallest and largest values within 1.5 × IQR, while outliers are not plotted as they are encompassed by the corresponding violin plot. The dotted line shows the median log2(summed intensity) for 2 min of WT infection across all three replicates.

Given the high number of stoichiometrically phosphorylated nucleic-acid-binding substrates (42 out of 82), we reasoned that T7K may bind to nucleic acids to focus its otherwise non-specific activity on these proteins. Feeding an AlphaFold3 model of full-length T7K (Extended Data Fig. 4a) into GraphBind led to the prediction that the C-terminal domain (residues Gln243–Gly359) contains an extended α-helix, enriched in basic residues capable of binding to RNA and/or DNA (Fig. 2e). Although dispensable for kinase activity^5,25^, this domain can shut off host transcription independently^32^, a process that was previously proposed to be mediated through tight DNA binding^5^, similarly to eukaryotic protamines.

To test T7K’s DNA/RNA-binding ability, we sought to purify the protein. Consistent with its known toxicity^25^, full-length T7K failed to express, even with a tight inducible expression system. However, we individually expressed the protein kinase (PK) domain (residues 1–242) and C-terminal SO domain (residues 243–359). While the PK domain was purified, the SO domain was insoluble (Extended Data Fig. 4b). We hypothesized that SO folding requires nucleic acids, and DNase I treatment during purification caused precipitation. Consequently, we isolated the SO domain from inclusion bodies for in vitro refolding. Urea-solubilized protein was refolded by slow dialysis into native conditions with or without *E. coli* genomic DNA (gDNA) or total RNA (Fig. 2f and Extended Data Fig. 4c). Analysis using quantitative mass spectrometry (MS) showed that gDNA, but not RNA, significantly increased SO solubility, whereas post-dialysis benzonase treatment reversed this effect (Fig. 2f). Thus, DNA stabilizes the SO domain, probably through direct interactions with its positively charged α-helix.

To further examine the SO domain function, we measured the phosphoproteome after infection with a T7 phage carrying a truncated T7K (ΔSO). Deletion of the SO domain reduced overall T7K-mediated phosphorylation (around 31% of phosphorylated peptides compared with the WT) (Fig. 2g and Supplementary Table 4), despite sampling at 10 min, when T7K is already autoinhibited (Extended Data Fig. 2c). Overall, the ΔSO mutant led to significantly less phosphorylation than the WT across nearly all proteins (Fig. 2h and Extended Data Fig. 4d). GO terms enriched in stoichiometric substrates for the WT (Fig. 2d) exhibited an even more pronounced reduction (Fig. 2i), consistent with the SO domain directing kinase preference to these targets through its DNA-binding abilities.

We hypothesized that deleting the SO domain reduces overall phosphorylation because the kinase autoinhibition is accelerated through autophosphorylation. Although the autoinhibitory Ser216 site was undetectable using tandem mass tag (TMT)-based MS, a prominent adjacent autophosphorylation site, Ser227 (ref. ^25^), showed no change between WT and ΔSO infections (Fig. 2h and Supplementary Table 4), implying equivalent kinase activity. To resolve the kinetics of phosphorylation of WT and ΔSO T7K, we monitored the phosphoproteome earlier in infection. While both phages yielded similar phosphorylation at 2 min, ΔSO-induced phosphorylation plateaued earlier compared with that induced by the WT (Fig. 2j and Extended Data Fig. 4e).

Together, these results reveal a dual role for the SO domain: directing kinase preference towards nucleic-acid-binding substrates, and sustaining kinase activity by delaying autoinhibition.

## T7K weakens activity of defence systems

Despite its broad specificity, T7K stoichiometrically phosphorylates nucleic-acid-binding proteins, thereby probably inactivating them. We wondered why T7 would do so given its reliance on intact host functions (for example, translation) for its infection cycle. Prompted by T7K-mediated phosphorylation muting the Rcs response^38,43^ (Fig. 2b and Extended Data Fig. 2g), a broad-spectrum antiphage defence^39,40^, we hypothesized that T7K targets nucleic-acid-binding proteins to broadly deactivate bacterial defence systems. Most known bacterial defence systems sense or target DNA, RNA and/or their bound proteins^7,16,44,45^. Such systems are scarce in *E. coli* K-12 (ref. ^46^), a historical host for phage biology, and other laboratory strains chosen for high DNA transformation efficiency, which would explain why this phenotype was overlooked.

**Fig. 3:**
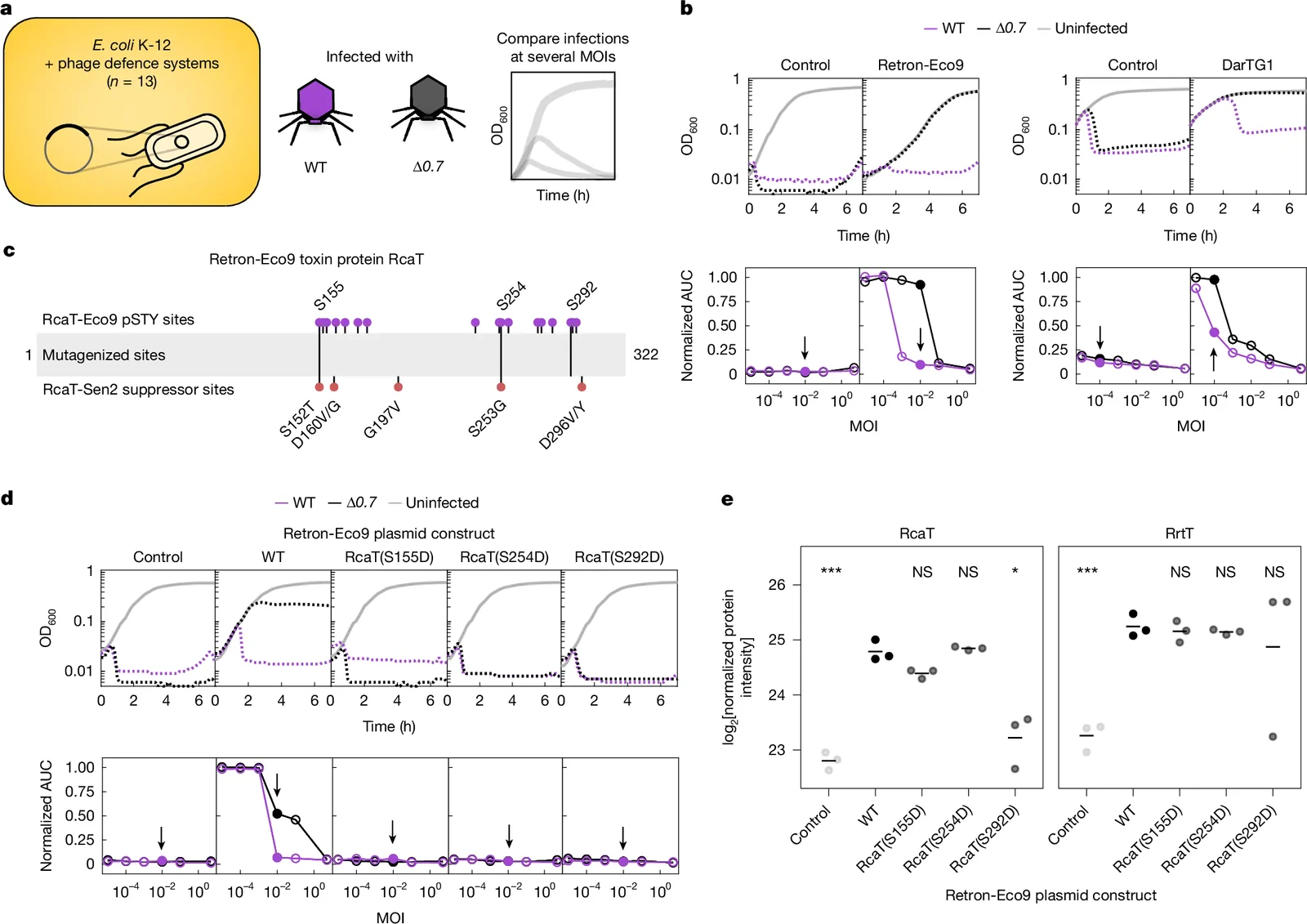
T7K weakens the DNA-related defence systems Retron-Eco9 and DarTG1 through phosphorylation. **a**, Screen of bacterial defence systems for T7K sensitivity. *E. coli* K-12 transformed with corresponding plasmids were infected at multiple MOIs with WT or Δ*0.7* phages; bacterial growth and population lysis were monitored over more than 6 h (the full results are shown in Extended Data Fig. 5a,b). **b**, Retron-Eco9 and DarTG1 defend against T7 and are T7K sensitive. For each system, a representative growth curve at a selected MOI distinguishing WT from the *0.7*-knockout mutant, and the area under the curve (AUC) normalized to the uninfected control (normalized AUC) over all tested MOIs is shown. The normalized AUC metric summarizes growth curves across MOIs and reflects phage efficiency and bacterial defence strength; more effective phages (bypassing defence systems) lead to a decrease in the normalized AUC at lower MOIs. **c**, Retron-Eco9-encoded RcaT is heavily phosphorylated during T7 infection. Phosphoproteomics analysis was performed 5 min after infection. *n* = 2 biological replicates. Class I phosphosites (*n* = 17 unique, PTMProphet score ≥ 0.75) along full-length RcaT are indicated by purple circles; sites tested as phosphomimetic mutants (**d**) are named on top. The red circles indicate aligned residues previously identified to be critical for function in homologous Retron-Sen2 RcaT^33^. **d**,**e**, Single phosphomimetic mutants in Retron-Eco9 RcaT abolish phage defence activity (**d**) without affecting protein stability in most cases (**e**). **d**, Representative growth curves and normalized AUC plots for *E. coli* K-12 transformed with the various plasmid alleles presented as in **b**. *n* = 1 biological replicate; the other replicate is shown in Extended Data Fig. 5f. **e**, The protein stability of mutant proteins was assessed by quantitative proteomics. *n* = 3 biological replicates. Statistical analysis was performed using two-sided unpaired *t*-tests comparing expression in each construct with those in the WT Retron-Eco9 construct; from left to right: *P* = 0.00018, 0.051, 0.65, 0.02, 0.00062, 0.62, 0.5, 0.7. Source data

**Extended Data Fig. 5:**
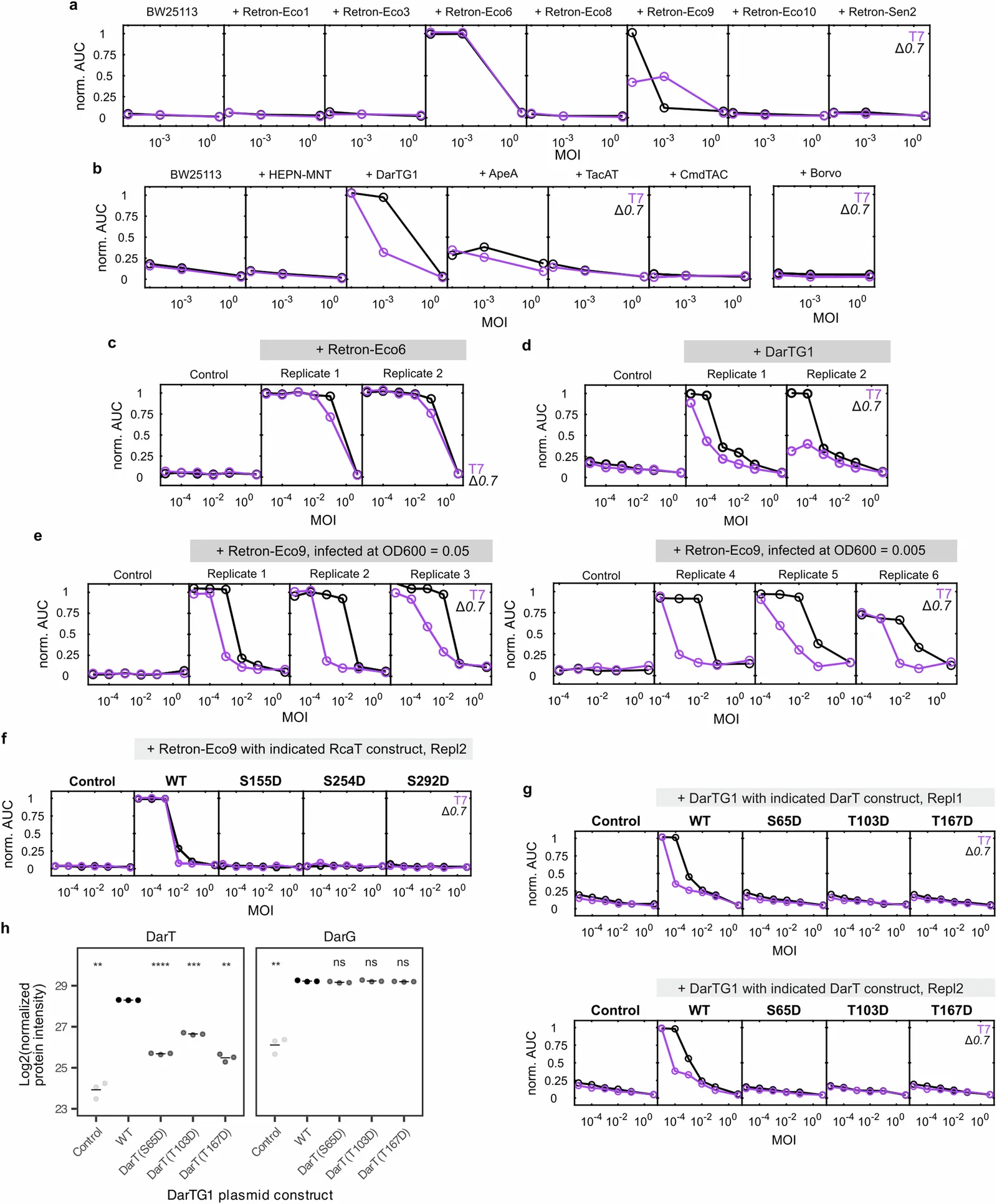
T7K weakens the activity of Retron-Eco9 and DarTG1. (**a**) Defence against T7 WT and Δ*0.7* by msDNA-containing retrons under native promoters. Normalized area under the curve (norm. AUC), normalized to the uninfected sample for all growth curves of the control BW25113 +/− defence systems infected with T7 (purple lines) or Δ*0.7* (black lines) at the indicated MOIs (empty circles). Matched growth curves are shown in Supplementary Fig. 2. **b**) Defence against T7 by DNA/RNA-binding systems (left-side plots) and one CHAT-protease domain-containing system (Borvo; right-side plot). (**c-e**) Norm. AUC graphs for all growth curves of the control BW25113 with empty plasmid or a Retron-Eco6 plasmid (**c**), a DarTG1 plasmid (**d**) and a Retron-Eco9 plasmid (**e**). All biological replicates shown - for DarTG1 and Eco9 biological replicates 1 and 2, respectively, are the same as in Fig. 3b. For Eco9, infecting at an earlier growth stage (OD600 = 0.005 vs. 0.05, measured with a pathlength = 1 cm) made the effect size of T7K-sensitivity more consistent. (**f**) Biological replicate of Fig. 3d. Normalized area under the curve (norm. AUC) for all growth curves of BW25113 with empty plasmid, Retron-Eco9 WT, or Retron-Eco9 RcaT mutants S155D, S245D, or S292D, infected with T7 (purple lines) or Δ*0.7* (black lines) at the indicated MOIs (circles), normalized to the uninfected sample. (**g**) Norm. AUC for all growth curves of BW25113 with empty plasmid or DarTG1 WT, or DarTG1 DarT mutants S65D, T103D, or T167D, infected with T7 (purple lines) or Δ*0.7* (black lines) at the indicated MOIs (circles), normalized to the uninfected sample. (**h**) Expression of the DarTG1 proteins DarT and DarG was investigated in *E. coli* K-12 transformed with either an empty plasmid (Control), DarTG1 in its wildtype form (WT), or with three phosphomimetic mutations of the DarT protein, in the absence of phage infection. The results of two-sided, unpaired t-tests comparing expression levels in each construct to those in the WT DarTG1 construct are shown, with ns meaning non-significant (p-value > 0.05), * meaning p-value ≤ 0.05, ** meaning p-value < 0.01 and *** meaning p-value ≤ 0.001 (from left to right: p = 0.00267, 2e-5, 0.00024, 0.00145, 0.0049, 0.1077, 0.8887, 0.2843). N = 3 biologically independent experiments. Source data

To examine whether T7K impacts phage defence systems, we selected 12 systems with different DNA/RNA links and one without (Supplementary Table 5). Seven were retrons, which contain multicopy single-stranded DNA (msDNA) or DNA–RNA hybrids as a structural part of an antitoxin that keeps the cognate (toxic) effectors inactive, and sense infection to trigger abortive infection^33,47–49^. We expressed these defence systems under their native or arabinose-inducible (pBAD) promoters in *E. coli* K-12 (BW25113), and infected the cells with T7 WT or Δ*0.7* at different multiplicities of infection (MOI), while monitoring bacterial growth (Fig. 3a, Extended Data Fig. 5 and Supplementary Fig. 2). T7 infection led to culture collapse within 1 h, even at the lowest MOI, due to T7’s rapid infection cycle (17 min) and burst size (around 180 virions per cycle)^50^. Three systems (Retron-Eco6, Retron-Eco9 and DarTG1) defended against T7 at least at the lowest MOI, confirming previous reports^33,48^. Retron-Eco9 and DarTG1 were counteracted by T7K. All of the other systems did not defend against both WT and Δ*0.7* T7 (Extended Data Fig. 5a,b). To better resolve the T7K anti-defence effect, we tested the three defending systems at a higher MOI range. Whereas Retron-Eco6 showed a negligible difference between WT and Δ*0.7* infection (Extended Data Fig. 5c), T7K strongly counteracted Retron-Eco9 and, to some degree, DarTG1 (Fig. 3b and Extended Data Fig. 5d,e).

To investigate whether T7K inactivates these defence systems, we profiled their phosphorylation during infection. Retron-Eco9 consists of reverse transcriptase RrtT and its cognate msDNA, which together counteract the toxic RcaT effector^33^. Although the exact mechanism of RcaT toxicity remains unclear, structural insights into homologous retrons exist and possible cellular targets have been proposed^51^. *E. coli* K-12 carrying natively expressed Retron-Eco9 exhibited 17 unique, confidently localized phosphosites on RcaT (Fig. 3c and Supplementary Table 6) 5 min after infection with WT T7. Two phosphosites, Ser155 and Ser254, aligned with residues that are critical for RcaT’s function in the homologous Retron-Sen2 (Fig. 3c). We designed phosphomimetic mutants (Ser to Asp) for these sites, alongside Ser181 and Ser292, which were near other functional residues in RcaT-Sen2. Despite failing to obtain the S181D mutant, all other three constructed alleles abolished Retron-Eco9 defence against T7 (Fig. 3d and Extended Data Fig. 5f). S155D and S254D compromised defence without affecting protein abundance, whereas S292D presumably caused protein destabilization (Fig. 3e). This implies that phosphorylation deactivates RcaT by reducing its activity or stability, thereby rendering the Retron-Eco9 sensitive to T7K anti-defence.

DarTG1 is a toxin–antitoxin system of which the toxin DarT ADP-ribosylates thymidines and guanosines on phage DNA to inhibit phage replication^34^. To probe T7K’s role on DarTG1, we used the same phosphoproteomics and phosphomimetic mutant analysis. DarT and DarG were heavily phosphorylated after T7 infection, exhibiting 19 and 18 phosphosites, respectively (Supplementary Table 6). We constructed three phosphomimetic DarT mutants: T103D, adjacent to the Glu152 active site that is essential for ADP-ribosylation in the structure, and S65D and T167D, bordering conserved protein regions. All three mutants abolished DarTG1 defence (Extended Data Fig. 5g). However, they also reduced DarT, but not DarG, abundance (Extended Data Fig. 5h), probably because they impacted its folding and/or stability. Although this precludes firm conclusions on whether phosphorylation directly alters DarT activity, phosphorylation of folded DarT at these sites during infection could still compromise its stability and defence.

In summary, T7K phosphorylates DNA-binding bacterial defence systems at or close to functionally relevant sites to disable their defence.

## T7K broadly counteracts phage defence

**Extended Data Fig. 6:**
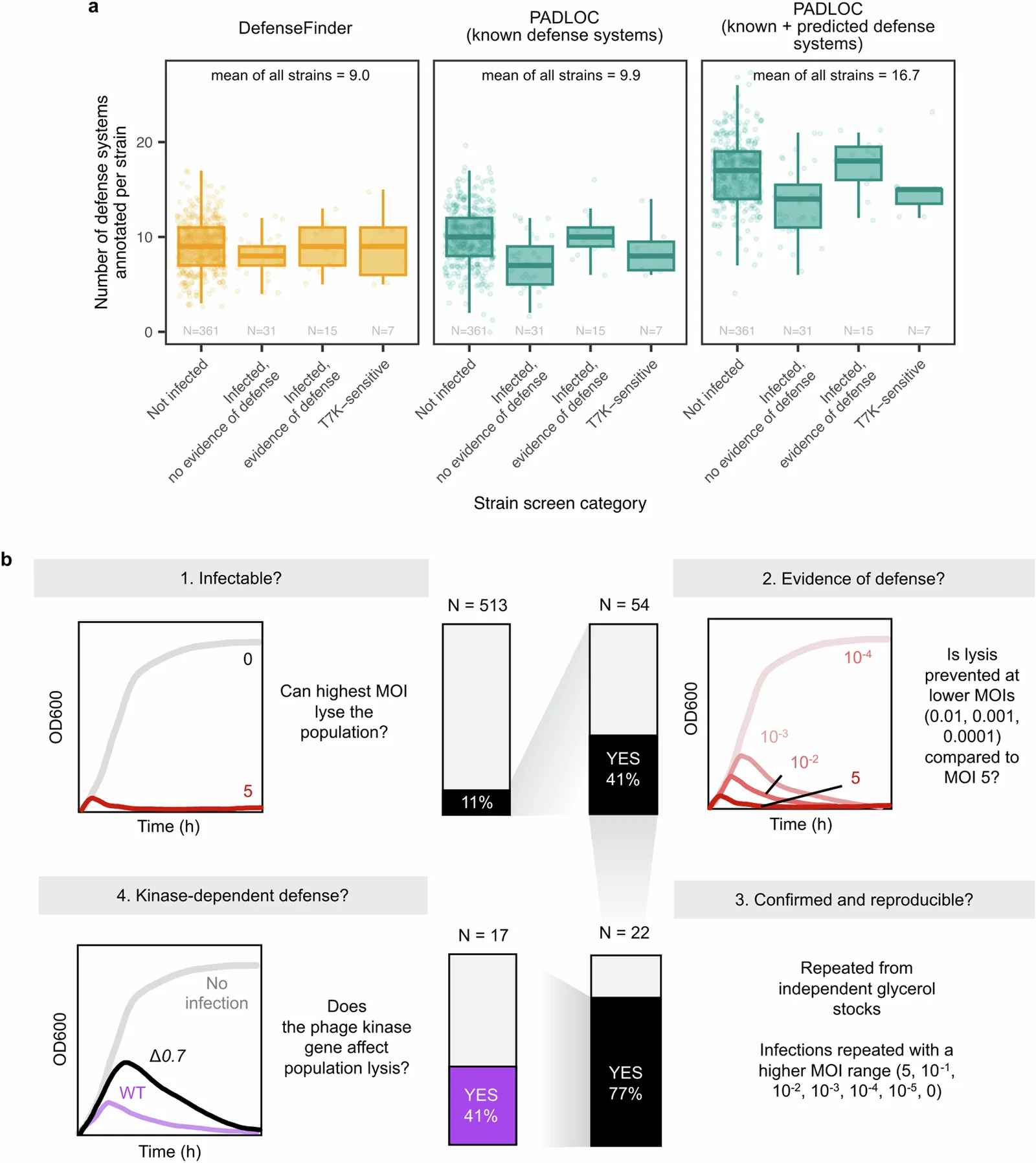
Overview of natural isolate library screen. (**a**) The number of defence systems detected within *E. coli* strains in the natural isolate collection which have sequenced genomes (N = 414), stratified according to the categories described in the following panel. Note: strains are categorized by their phenotypes in the initial screen (Supplementary Fig. 3), except for the fourth category which is made up of the 7 strains with validated T7K-sensitive defence, reported in Fig. 4b. Strains were annotated by the PADLOC^113^ and DefenseFinder^6^ tools. PADLOC also annotates phage defence candidate systems, with the prefix “PDC-”, which are not yet all experimentally validated to confer defence, so two distributions of the number of defence systems per strain for this tool are shown. The mean of the entire group is shown above the plot, and the mean and N (number of strains) for the subgroups are indicated on the boxplot. Boxplots are shown as in Extended Data Fig. 2i. Boxplots show the data distribution, with the box spanning the interquartile range (25th and 75th percentile) and the median indicated at the line. Whiskers extend to the smallest and largest values within 1.5 × IQR, while outliers are not plotted as they are encompassed by the underlying data points. (**b**) Schematic showing the categorization of strains in the natural isolate screen during the screen for strains that are sensitive to the presence of the T7K during infection by T7 (as in Supplementary Figs. 3 and 4). N indicates the number of strains passing each filter, for example 54 strains were infectable by T7, and 22 T7-infectable strains had evidence of some existing defence against T7 infection. Results from these 22 strains (including those suggesting kinase-sensitive and kinase-insensitive defence) were then validated from independent single glycerol stocks and using a higher range of MOIs. Strains that passed this validation are the strains shown in Fig. 4b,c.

Most of the >150 phage defence systems identified lately have been validated by being expressed from plasmids (15–30 copies per cell) in heterologous hosts^11,49,52^. As T7K needs to phosphorylate proteins with high stoichiometry to deactivate them, we reasoned that its anti-defence role may be more pronounced when defence systems are expressed at native levels. To test T7K under physiologically relevant conditions, we infected a library of 513 *E. coli* natural isolates, comprising laboratory, commensal and pathogenic strains^53^. Annotating known defence systems in these isolates revealed an average of 9.0 or 9.9 systems per isolate, depending on the tool used (Extended Data Fig. 6a and Supplementary Table 7).

**Fig. 4:**
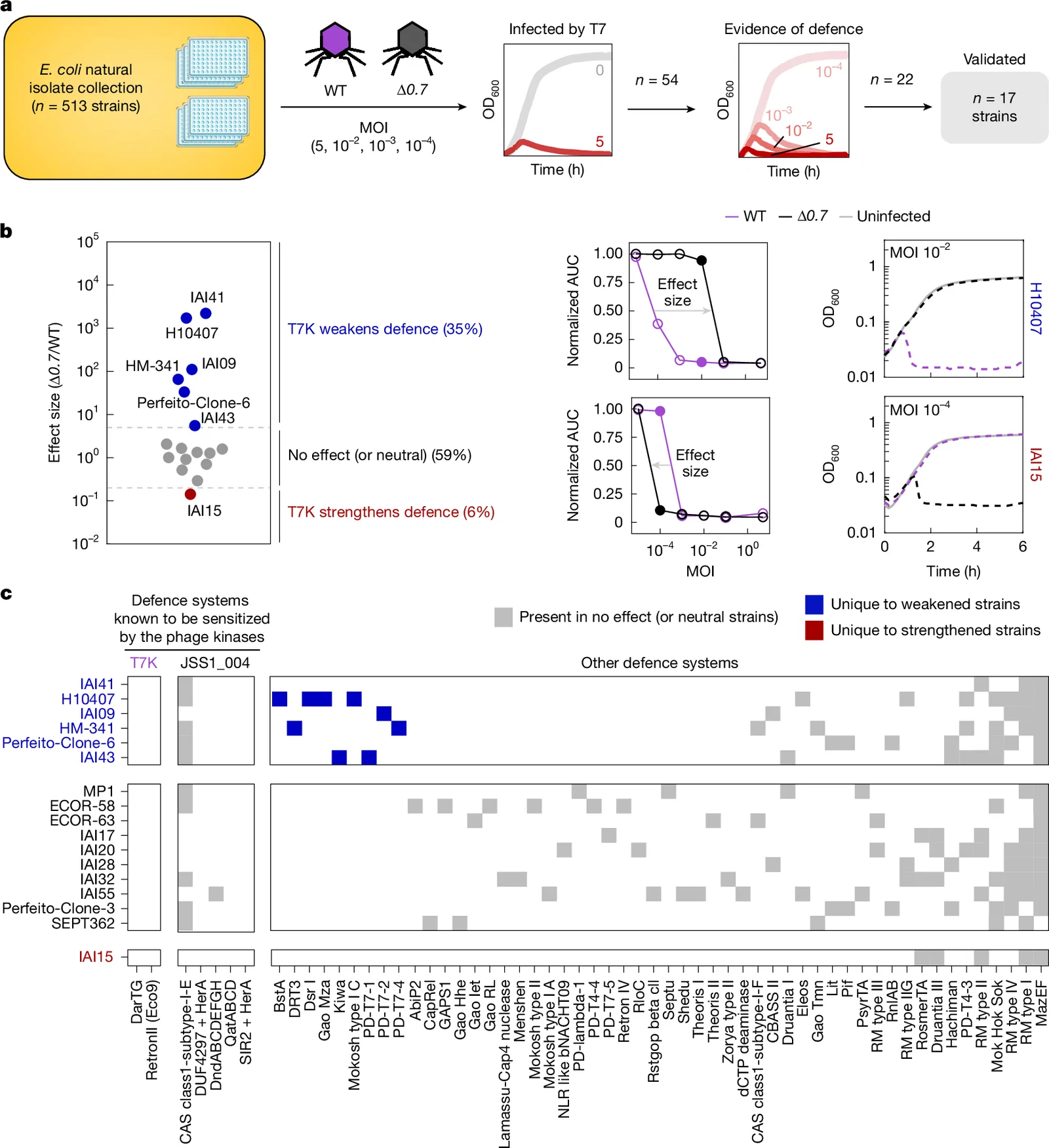
T7K helps T7 to infect diverse natural *E. coli* isolates, pointing to a broad anti-defence role. **a**, The screen design to test for dependency on T7K during liquid infection assays of the ECOREF *E. coli* natural isolate library^53^ with WT and Δ*0.7* T7. Infectivity and evidence of defence were assessed in the screen (one replicate), and strains that passed were further validated using an extensive MOI and at least two biological replicates. **b**, A substantial fraction of strains that are infectable by T7 exhibit defence that is sensitive to T7K. The effect size of sensitivity to T7K during infection for those strains that were infectable and had evidence of defence was validated by independent assays. Effect sizes were estimated as the ratio (Δ*0.7*/WT) of the interpolated MOI at 50% AUC (Methods). Strains were categorized as containing defence systems that were weakened by T7K (blue, effect size ≥ 5), strengthened by T7K (red, effect size ≤ 0.2) or not affected (grey) (all replicates are shown in Extended Data Fig. 7a). An example growth curve at a single MOI and the corresponding full normalized AUC graph are shown for a case of weakened and strengthened T7 defence. **c**, T7K-sensitive natural isolates carry different defence systems, which are potential targets of T7K. DefenseFinder^6^ defence system profiles of validated strains grouped by their T7K-sensitivity categorization as defined in **b**. Defence systems are grouped on the basis of (1) whether we identified them to be T7K-dependent; (2) whether they are sensitive to a homologous phage protein kinase JSS1_004 (ref. ^23^); or (3) otherwise. The colour indicates whether the system was uniquely found in weakened or strengthened strains, or whether it was also found in the no-effect strains. Source data

**Extended Data Fig. 7:**
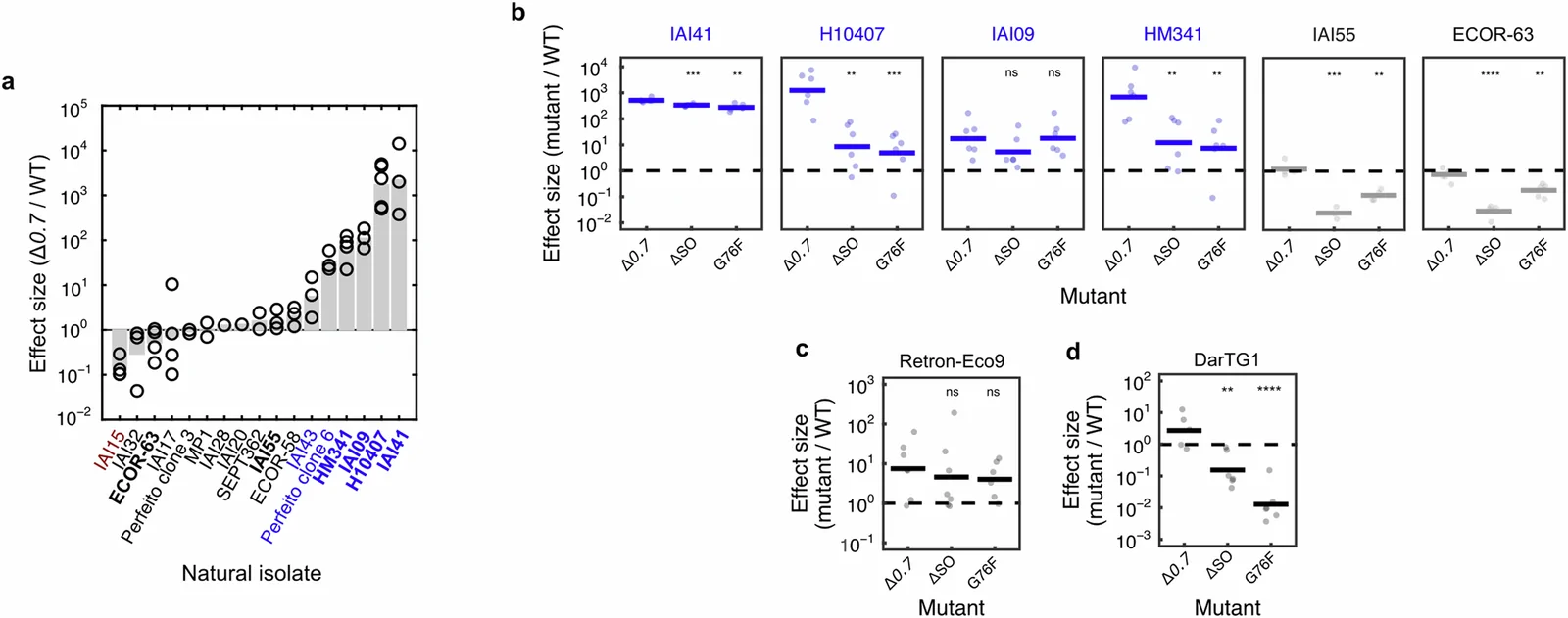
Infection assays on natural isolates and using partial T7K mutants. (**a**) Mean log10(effect sizes) (grey bars) and replicate values (black circles) (n = 3 or more individual biological replicates, see Supplementary Fig. 4a), estimated for validated strains across biological replicates (see Methods) comparing Δ*0.7* to WT T7 fitness. Effect sizes were calculated from the normalized AUC curves shown in Supplementary Fig. 4a. Outlier replicates without defence (one biological replicate for H10407 and HM-341 each, see Supplementary Fig. 4a), or with nearly full defence (two biological replicates for IAI20 and IAI28 each, see Supplementary Fig. 4a) were excluded from the analysis. If the lowest MOI did not allow defence against a specific phage, the effect size was estimated assuming that a 10-fold lower MOI would allow defence against T7, which may even underestimate the actual effect size. Bolded strains show those investigated further in panel b, and colours represent T7K-sensitivity status as in Fig. 4b. (**b**) Mean log10(effect sizes) (crossbars) and replicate values (dots) (n = 4 or more individual biological replicates, see Supplementary Fig. 5a) estimated for selected natural isolates testing fitness of the expanded T7 mutant panel (Δ*0.7*, ΔSO and G76F catalytic dead) against WT T7. For these experiments, effect sizes were calculated using an expanded MOI range (5, 1, 0.5, 10^−1^ serially diluted 1:10 down to 10^−8^). Blue strains are those determined to be highly T7K-sensitive, and black strains are control, i.e. T7K-neutral strains. If the highest MOI did not lyse the population (mutants for IAI41), the effect size was estimated assuming that a 10-fold higher MOI would lyse the population. If the lowest MOI did not allow defence against a specific phage, the effect size was estimated assuming that a 10-fold lower MOI would allow defence against T7. The results of unpaired t-tests comparing log10(effect sizes) from partial mutants (ΔSO and G76F) to the full deletion (Δ*0.7*) are shown, with ns meaning non-significant (p-value > 0.05), ** meaning p-value ≤ 0.01, *** meaning p-value ≤ 0.001 and **** meaning p-value ≤ 0.0001 (from left to right: IAI41 - 0.00071, 0.0015; H10407 - 0.001, 0.0005; IAI09 - 0.19, 0.97; HM341 - 0.0067, 0.0044; ECOR-63 - 6.5e-6, 0.001; IAI55 - 0.00041, 0.0018). Effect sizes were estimated from the normalized AUC curves shown in Supplementary Fig. 5a. (**c**) Same as for panel b but showing the results of infection assays on BW25113 expressing Retron-Eco9 (n = 7 individual biological replicates; from left to right - p = 0.62, 0.42) or (**d**) DarTG1 (n = 6 individual biological replicates; from left to right - p = 0.0039, 6.1e-5). Effect sizes were estimated from the normalized AUC curves shown in Supplementary Fig. 5b for Retron-Eco9 and 5c for DarTG1. Source data

We infected the *E. coli* isolates during exponential growth with WT and Δ*0.7* T7 at four MOIs (5, 10^−2^, 10^−3^ and 10^−4^), alongside an uninfected control for each strain. We classified three features: (1) T7 susceptibility (at MOI = 5); (2) T7 resistance (lysis prevented at MOI < 5); and (3) T7K-sensitive defence (response differences between WT T7 and Δ*0.7*) (Fig. 4a and Extended Data Fig. 6b). Out of 513 strains (Methods), 54 (10.5%) were susceptible to T7 infection (Fig. 4a, Extended Data Fig. 6b and Supplementary Fig. 3). This low infection rate was expected, as T7 infects only ‘rough’ *E. coli* strains lacking *O*-antigen^54^; however, we cannot exclude that some uninfected strains carry systems providing complete defence. Among the 54 infectable strains, 22 displayed some defence against T7 (Fig. 4a and Supplementary Fig. 3). Evaluating these 22 strains at a higher MOI resolution led us to exclude five with inconsistent phenotypes across biological replicates, mostly due to low or no infection (Fig. 4a and Supplementary Fig. 3). From the 17 validated strains, T7K reproducibly weakened the defence of 6 strains, strengthened 1 strain and had negligible effects in 10 strains (Fig. 4b, Extended Data Fig. 7a and Supplementary Fig. 4). The effect size of T7K-mediated defence weakening spanned three orders of magnitude, exceeding 1,000-fold in two genetically diverse strains (H10407, IAI41) (Fig. 4b and Extended Data Fig. 7a), pointing to different defence systems being counteracted. Indeed, the defence systems harboured by these strains shared no single common system absent from the ten no-effect/neutral strains (Fig. 4c and Supplementary Table 7). Notably, hit strains lacked the T7K-sensitive systems identified here (Retron-Eco9, DarTG), others functionally linked to T7K (Retron-Eco2)^55^ or those recently described to be counteracted by the JSS1 phage protein kinase, which has some homology to T7K (Dnd, QatABCD, SIR2–HerA, DUF4297–HerA, CRISPR type I-E)^23^. This suggests that T7K inactivates a broad swath of diverse unidentified defence systems. It also implies that defence systems can evolve to evade T7K anti-defence (for example, Dnd is present in the neutral strain IAI55; CRISPR type I-E is present in most strains) or even sense T7K to trigger activation, possibly explaining why IAI15 defends better against WT T7 than against Δ*0.7* T7. This sensing may happen directly through phosphorylation or indirectly through T7K-induced host transcription shutoff, as recently proposed for the ShosTA toxin–antitoxin system^56^.

As T7K acted against both overexpressed defence systems and natural isolates, we assessed whether the SO domain^24,32,57^ also had a role in this (Extended Data Fig. 7b,c and Supplementary Fig. 5a,b). The SO domain seemed to have a direct role, independent of the kinase activity (G76F mutant), in defence inactivation of at least some of the defence systems (those in H10407 and HM-341). This role remains to be further elucidated, but may be linked to its reported role on RNA-polymerase inhibition^58^. We also noticed that the two partial mutants (catalytic or SO domain) were fitter than the full T7K deletion when infecting neutral strains (ECOR-63, IAI55). Although we cannot fully explain this (possibly due to slightly different genetic backgrounds of the different phages, for example, *cmk* marker in full deletion), this precluded us from rigorously assessing the role of individual domains in systems with small effect sizes, such as DarTG1 (Extended Data Fig. 7d and Supplementary Fig. 5c).

Overall our natural isolate screen (Fig. 4), targeted experiments (Fig. 3) and other studies of homologous phage kinases^23^ point to a broad anti-defence role for T7K against diverse bacterial immunity systems. T7K uses its C-terminal DNA-binding domain to direct its promiscuous kinase activity towards nucleic acid-binding proteins, which are common in bacterial immunity systems.

## T7K homologues are promiscuous kinases

**Fig. 5:**
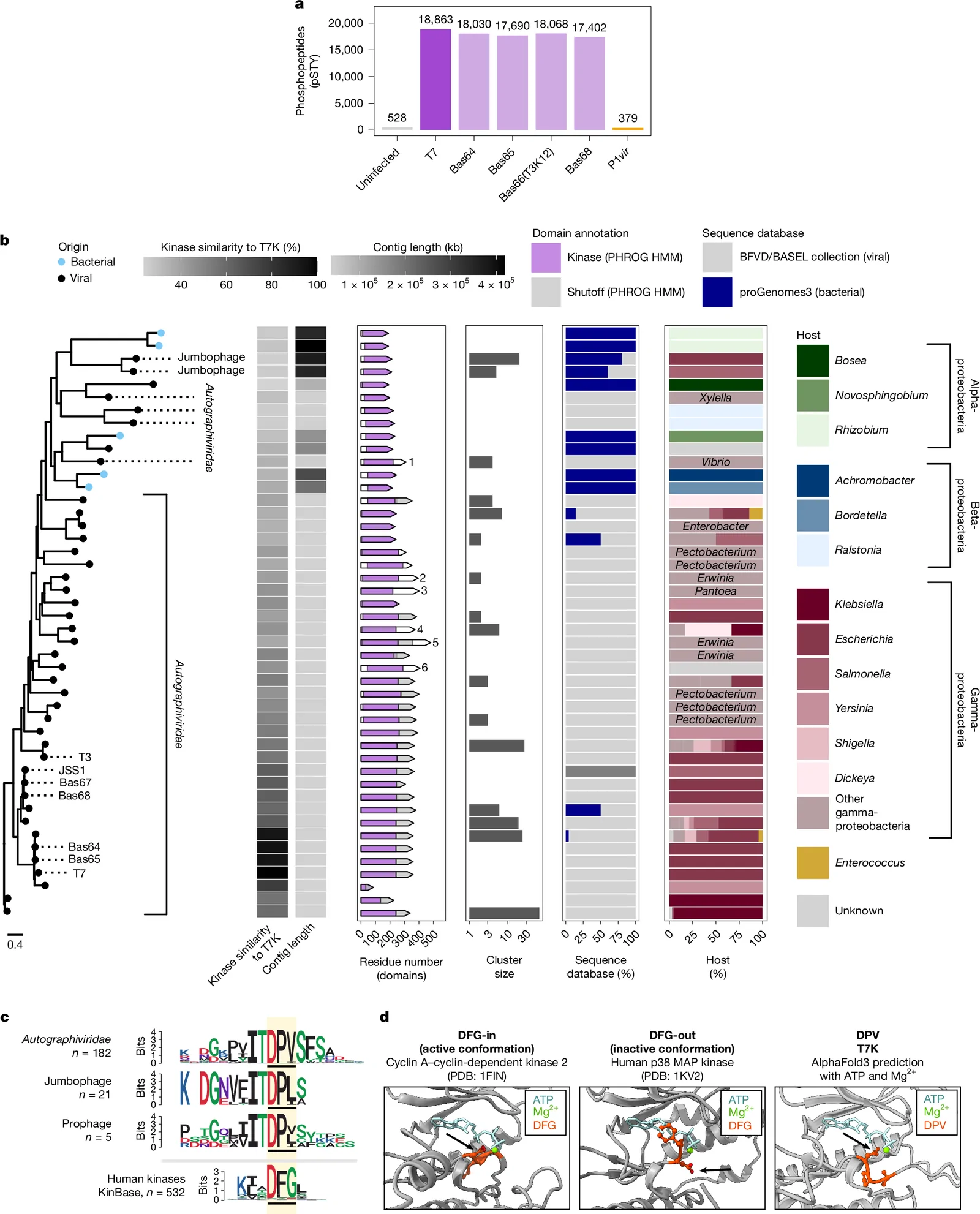
Conserved hyperactivity and sequence features of T7K homologues. **a**, Hyperactivity is a conserved, unique feature of T7K homologues. The total number of unique Ser/Thr/Tyr phosphopeptides (*n* = 3 biological replicates phosphoenrichment, label-free MS/MS) of uninfected or phage-infected *E. coli* K12 is shown. Light purple indicates *Autographiviridae* infections from the BASEL collection^59^ (encoding T7K homologues); orange indicates P1*vir* (encodes unrelated Doc kinase). Bas64, Bas65 and Bas66(T3K12) were sampled at 10 min, Bas68 at 5 min and P1*vir* at 45 min. **b**, T7K homologues are common among *Autographiviridae*, but are also found in putative prophages and jumbophages with distinct domain architectures and host range. A phylogenetic tree of clustered kinase domains from viral/bacterial sequence databases is shown, reduced to 46 representatives at 80% sequence identity (all hits are shown in Supplementary Table 8). Jumbophage labels represent hits including *Salmonella* phage Munch and *Escherichia* phage G17. The blue tips represent putative prophage homologues originating from bacterial genomes where a prophage-like region lacking bacterial marker genes but containing a T7K-like protein (Extended Data Fig. 9c) is bordered by several bacterial marker genes. The labels 1–6 next to the gene arrows represent putative homologues with divergent C termini that were further structurally investigated (Extended Data Fig. 9b). **c**, T7K homologues contain distinct, DFG-like motifs. Sequence motif logos show residue conservation around the kinase active site in identified T7K homologues from *Autographiviridae*, jumbophages or prophages. The DPV motif corresponds to T7K residues 213–215 (UniProt: P00513). Human kinase domain conservation (KinBase)^71^ is shown as a reference. **d**, The T7K DPV motif aligns with the DFG motif structural position in the kinase active site and mimics its active-state conformation. Magnified views of the ATP-binding site after aligning DFG-in and DFG-out structures (PDB: 1FIN, 1KV2) to the AlphaFold3 T7K-Mg^2+^-ATP model (Extended Data Fig. 9d). DFG/DPV residues are shown in orange with side chains; the arrows indicate the Asp position.

**Extended Data Fig. 8:**
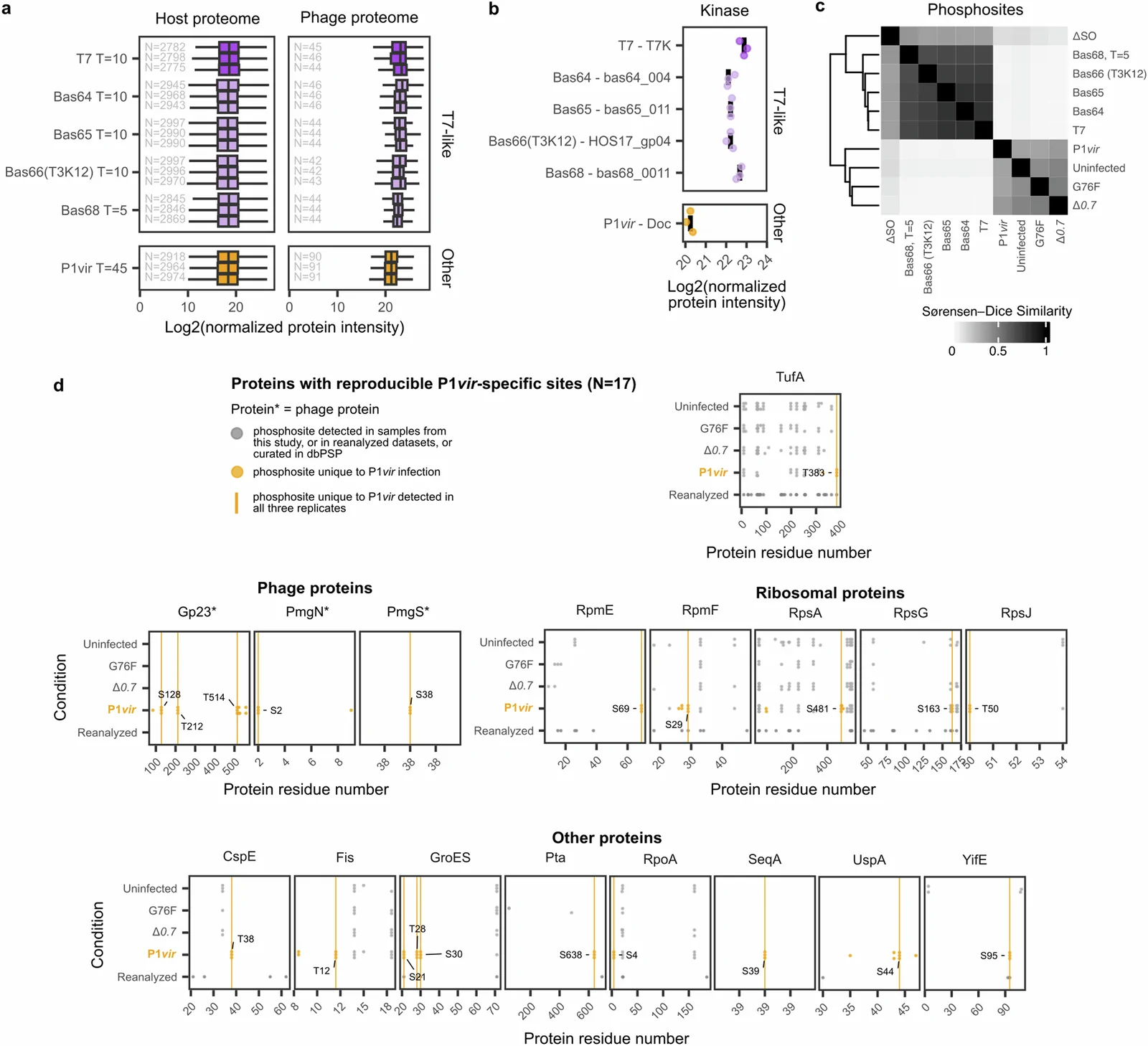
Proteomics and phosphoproteomics of phages encoding diverse kinases. Protein expression analysis (label-free data-independent analysis) was performed on samples from Fig. 5a to (**a**) confirm progression of phage infection cycle and (**b**) confirm expression of kinases at the sampled time-points. For both panels, the results from three independent biological replicates are shown. Boxplots show the data distribution, with the box spanning the interquartile range (25th and 75th percentile) and the median indicated at the line. Whiskers extend to the smallest and largest values within 1.5 × IQR, while outliers are not plotted. N= indicates the number of underlying proteins (with at least one unique peptide) quantified in each replicate. In (b), the gene name and/or locus tag of the relevant kinase/s is indicated next to the virus name. (**c**) Similarity matrix of different infections based on identified phosphosites. Sites were derived from phosphopeptides reported in Fig. 1b (Δ*0.7*, G76F), Fig. 2g (ΔSO) and Fig. 5a (uninfected, T7, BASEL phages, P1*vir*), each from three biological replicates. Rows and columns represent individual infections. The Sørensen-Dice coefficient quantifies how similar two sets are, emphasizing the proportion of shared elements relative to their combined size, where similarity values range from 0 (no overlap; white) to 1 (identical; black). Rows are hierarchically clustered using average linkage based on their dissimilarity, i.e. 1 - Dice similarity, with the resulting dendrogram shown. (**d**) Proteins uniquely and reproducibly phosphorylated during P1*vir* infection. Each dot is an identification of a phosphopeptide at the indicated site in one of three independent biological replicates from label-free data-dependent analysis of *E. coli* K12 after infection with the indicated phages. The same datasets as detailed above in panel c were considered, except sites from WT and ΔSO T7 infections which were neither considered nor plotted. Note: all phosphopeptides are considered regardless of uniqueness within the FASTA database to enable visualization of the known Doc kinase site at TufA−383 (represented by a peptide sequence shared between TufA and TufB). Sites in the dbPSP database and those from the reanalyzed phosphoproteomics datasets in Extended Data Fig. 1a are also shown but are not considered when assessing uniqueness to P1*vir* infection. Orange dots indicate sites that only come up in P1*vir*, with a vertical orange line indicating the further subset of these sites which were identified in all three replicates. Proteins with an asterisk are phage proteins.

To investigate whether hyperpromiscuous kinase activity is common in phages, we searched for protein kinases within the BASEL collection of *E. coli* lytic phages^59^. We studied four phages of the *Autographiviridae* family that encode T7K homologues (Bas64, Bas65, Bas66(T3K12) and Bas68) and P1*vir*, which carries an unrelated kinase^60^. We sampled a single timepoint during *E. coli* K-12 infection: 10 min for the T7-like phages (at 5 min for Bas68 owing to its faster infection) and at 45 min for P1*vir*. Expression proteomics confirmed infection, detecting most phage proteins, including the kinases (Extended Data Fig. 8a,b). Phosphoproteomics analysis revealed extensive phosphorylation triggered by all *Autographiviridae* phages, mirroring T7 with T7K, but not by P1*vir* (Fig. 5a, Extended Data Fig. 8c and Supplementary Table 8). Among these phages, the Bas68 kinase closely resembles JSS1_004 from *Salmonella* phage JSS1 (95% sequence identity within the kinase domain) (Fig. 5b), which was reported to be unspecific^23^, albeit to a lower degree (around 20-fold lower phosphorylation) than what we observed for T7K. Given their high similarity and the kinase hyperpromiscuity across all tested *Autographiviridiae*, the reported activity difference for T7K and JSS1_004 probably arises from the more sensitive phosphoproteomics approach used here.

Conversely, phosphoproteomics analysis of P1*vir* infection revealed that not all phage kinases are hyperpromiscuous. Despite its low expression during infection (Extended Data Fig. 8b), we reproducibly detected the single known phosphorylation site of the unrelated-to-T7K Doc kinase (TufA-T383)^60^. P1*vir* infection produced a phosphoproteome profile distinct from uninfected samples, T7 infection and T7 mutants (Extended Data Fig. 8c), with 20 additional sites across 17 proteins identified reproducibly and uniquely during P1*vir* infection (Extended Data Fig. 8d and Supplementary Table 8), including three phage proteins. These sites may represent undescribed Doc substrates or a P1*vir-*specific host response.

In summary, while T7K hyperpromiscuity and hyperactivity are conserved across *Autographiviridae* homologues, these are not general features of phage-mediated kinase signalling.

## T7K homologues possess a unique motif

To investigate the evolutionary origins and diversity of T7K, we searched for putative homologues in viral and bacterial genome databases. We found 190 viral (BFVD^61^, BASEL collection^59^) and 32 bacterial proteins (progenomes3 (ref. ^62^)) with substantial similarity to the T7K kinase domain (Supplementary Table 8). We clustered hits at 80% sequence similarity to remove redundancy, yielding 46 clusters (Fig. 5b).

**Extended Data Fig. 9:**
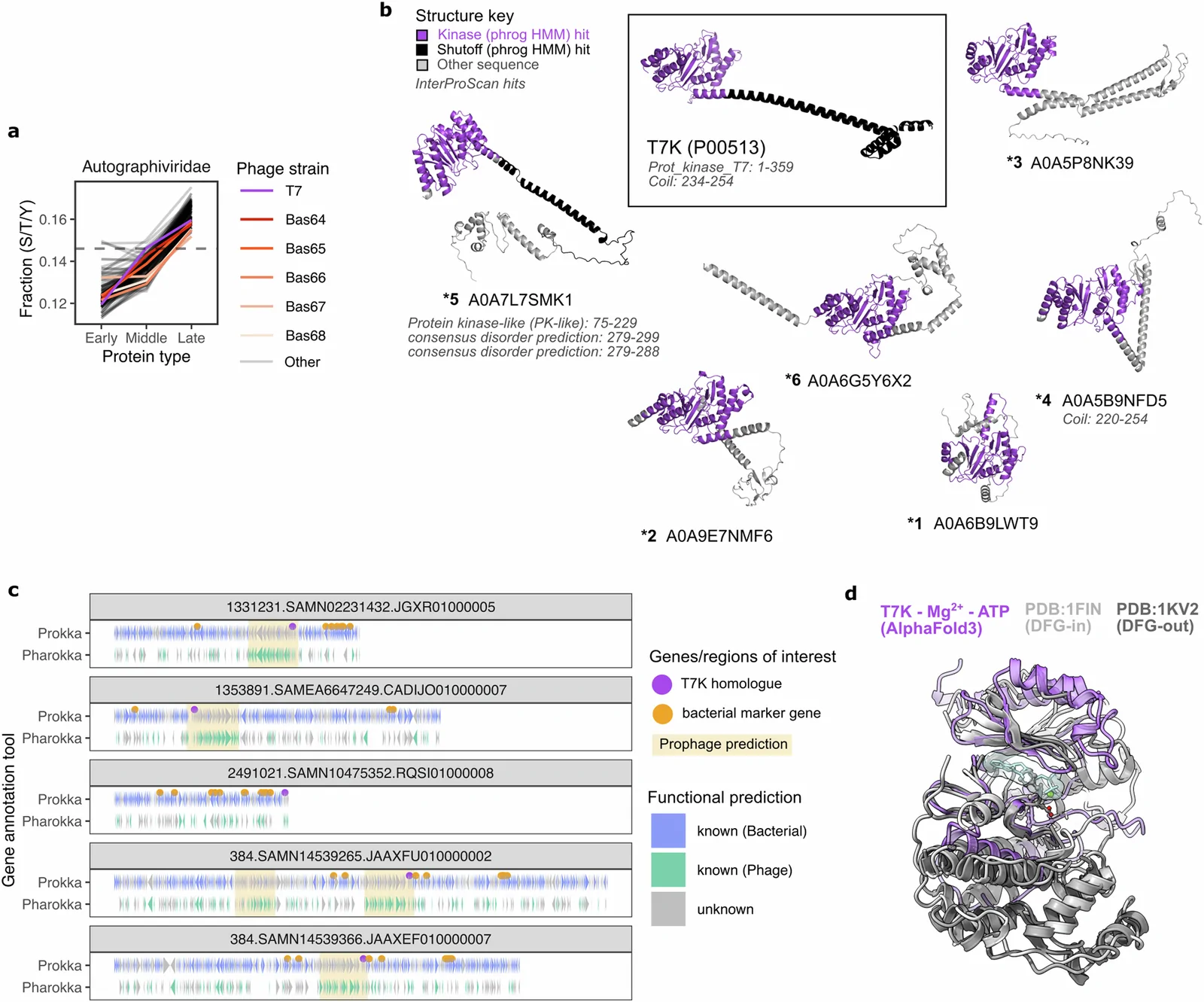
Features and quality control of T7K homologue search and analyses. (**a**) Cumulative fraction of serine (S), threonine (T) and tyrosine (Y) in early, middle and late genes over all *Autographiviridae* genomes with a T7-like kinase. We defined the boundaries of early, middle and late genes according to the boundary genes in Dunn et al.^41^ and transferred the information to other *Autographiviridae* by identifying homologues of key genes (gp1.3 and gp6.3) at T7 early/middle/late group boundaries using BLAST. (**b**) AlphaFold3 predictions for clade-representing homologues from Fig. 5b with non-canonical domain architectures. These proteins had kinase (phrog HMM) matches (purple residues) but also had a significant stretch of their sequence without hits to the shutoff (phrog HMM) (grey residues). T7K is also shown for reference in the box. A0A7L7SMK1 also contained a shutoff (phrog HMM) hit (black residues). Regions corresponding to hits from InterProScan5 are shown in italics below the protein name (UniProt accessions). The numeric labels next to the structures are also indicated next to the gene arrows in Fig. 5b. (**c**) Putative prophage regions carrying a T7K homologue. ORF-calling and functional predictions were carried out using both a bacteria-focused genome annotation tool (Prokka^122^) and phage-focused genome annotation tool (Pharokka^123^). Regions in yellow correspond to predicted prophage regions called by PHASTEST. The purple dot highlights the locus encoding the T7K homologue; orange dots highlight bacterial marker genes identified with gtdb-tk. (**d**) Global structural alignment of T7K kinase and DFG-in and DFG-out motif structures. The AlphaFold3 prediction for T7K in complex with Mg^2+^ and ATP (purple, only kinase domain shown) was aligned with experimental structures representing DFG-in (PDB:1FIN; light grey) and DFG-out conformation (PDB:1KV2; dark grey). The same perspective is shown as in the Fig. 5d insets. Only the ATP (blue) and Mg^2+^ (green) molecules in the AlphaFold prediction are shown.

Most viral entities with a putative T7K homologue belonged to *Autographiviridae* (T7 family), and had similar genome sizes to T7 (Fig. 5b and Supplementary Table 8). This group contained the experimentally validated BASEL phages (Bas64, Bas65, Bas66(T3K12) and Bas68), which were among the closest T7K homologues. Early proteins in nearly all *Autographiviridae* phages with T7K homologues were depleted in Ser/Thr/Tyr residues (Extended Data Fig. 9a), suggesting that kinase promiscuity exerts positive selection on these genomes. *Autographiviridae* variants mostly retained the T7K domain architecture, with the SO domain following the kinase domain. We found no alternative domains in the few variants with divergent C-terminal sequences (labelled 1–6 in Fig. 5b and Extended Data Fig. 9b). More distant variants included two stemming from jumbophages (approximately 400 kb genome size), including proteins from lytic *Salmonella* phage Munch and *Escherichia* phage G17. Both phages frequently appear in bacterial genome assemblies owing to contamination or unresolved aspects of their lytic cycle^63^ explaining why most members of these two clusters were detected in the progenomes3 database rather than BFVD (Fig. 5b). After filtering probable viral contamination from the bacterial database, we only sporadically detected T7K homologues within bacterial contigs (*n* = 5) (Fig. 5b (light blue leaves)). These represented single instances across phylogenetically diverse hosts, mostly within predicted prophages (*n* = 4) (Extended Data Fig. 9c). Both the jumbophage and the putative bacterial-origin kinases have diverged from the T7K, and lost the SO domain (Fig. 5b); their function and promiscuity remain to be elucidated.

In most eukaryotic kinases, catalytic activity is partially governed by a conserved DFG motif. T7K homologues retained a distinct, highly conserved DPV motif in *Autographiviridae* homologues; the valine is absent or less conserved in jumbophage and prophage-encoded T7K homologues (Fig. 5c). The canonical DFG motif is part of the kinase activation loop and adopts two major conformations, DFG-in and DFG-out^64^ (Fig. 5d). In the DFG-in state, the aspartate coordinates with Mg^2+^ to position ATP for hydrolysis. Conversely, in the DFG-out state the rotated phenylalanine displaces the aspartate from the Mg^2+^, rendering the kinase catalytically inactive (Fig. 5d). The DPV motif occupies the same structural location in the AlphaFold3-modelled T7K structure as DFG residues in ATP-binding sites of experimentally resolved kinase structures (Fig. 5d and Extended Data Fig. 9d), but lacks the phenylalanine that displaces ATP-binding in the DFG-out conformation. To understand how this change affects T7K, we compared the T7K AlphaFold prediction with X-ray structures representing DFG-in and DFG-out conformations (Fig. 5d). These comparisons support that the T7K and its binding pocket are more similar to the DFG-in conformation. They also indicated that the DPV aspartate maintains its geometry for a productive coordination with the Mg^2+^–ATP complex (Fig. 5d (black arrow)), and that the proline has potential to support this arrangement and maintain a favourable aspartate–Mg^2+^–ATP interaction, enabling higher kinase activity. Notably, the two reported T7K autophosphorylation sites^25^ occur immediately downstream of the DPV motif; Ser216 (reported inhibitory site) is conserved in all variants and Ser218 is prevalent in the *Autographiviridae* variants (Fig. 5c), positioning them to affect the activation loop. Overall, it seems plausible that the DPV motif locks T7K in a constitutively active DFG-in-like form, while autophosphorylation deactivates this highly processive kinase.

## Discussion

Here we show that T7K is a hyperpromiscuous, dual-specificity kinase that is capable of phosphorylating any protein of the infected host without any sequence motif preference. Its substrate range exceeds that of any known kinase, including all human kinases combined. T7K probably achieves this activity through its DFG-like DPV motif, which maintains the active site in a constitutively on conformation. Through its DNA-binding C-terminal SO domain, this loose-cannon kinase achieves stoichiometric phosphorylation of DNA- and RNA-binding proteins within its short window of activity—the latter group probably being targeted owing to transcription and translation coupling in prokaryotes. The phage uses this highly stoichiometric phosphorylation to deactivate proteins that sense or attack its DNA, and possibly its RNA. To mitigate the dangers of uncontrolled phosphorylation, T7 inactivates the kinase within 6 min after infection through autophosphorylation^25,37^. This inactivation timing is regulated at least partially through the SO domain, probably through DNA interactions which reduce the pool of free-floating T7K. The degree to which the SO domain acts directly or through the kinase domain in defence inactivation remains to be elucidated. This highly promiscuous and processive kinase activity sets a paradigm of how phosphorylation can be used in living organisms to deactivate molecular machines. Notably, and despite its vast impact, the role of the T7K has remained unclear for almost 50 years. This is because earlier tools lacked sensitivity for identifying phosphorylated substrates^1–3,25–27^, and because T7 infection was studied in laboratory strains with limited phage-defence repertoires.

Close T7K homologues reside almost exclusively in phages—the few sporadic instances in bacterial genomes (prophage sites) require further verification to exclude genome assembly artefacts. This suggests that hyperpromiscuous phosphorylation could be an ancestral strategy incompatible with selective Ser/Thr/Tyr signalling in complex organisms. Future studies on more distant T7K homologues identified here, and their relation to known dual-specificity kinases in bacteria and eukaryotes, may clarify how such kinases became domesticated by tighter activity control and/or by altered specificity. Other prophage-encoded Ser/Thr kinases involved in phage defence such as Stk2 have also been suggested to phosphorylate a broader substrate clientele than currently appreciated^22^. Whether these kinases are promiscuous remains to be explored using sensitive phosphoproteomics methods. More generally, Ser/Thr/Tyr phosphorylation has been considered to be less prevalent in prokaryotic signalling based on the fewer Ser/Thr or Tyr kinases and phosphosites found in bacteria compared with in eukaryotes^65^. Our work shows that Ser/Thr/Tyr phosphorylation can be more pervasive in bacteria during phage infection than in eukaryotic cells.

We identified two DNA-binding/targeting defence systems that are sensitive to T7K, due to critical Ser/Thr surface residues. The decreased ability of T7 lacking the kinase (Δ*0.7*) to bypass several natural *E. coli* isolates suggests that T7K counteracts many additional defence systems. Recent studies corroborate our findings. Using barcoded mutant libraries, one study identified an important role for T7K when infecting strains carrying Retron-Eco2 (ref. ^55^), presumably because T7K deactivates this defence. Studies on *Salmonella* phage JSS1, which carries a kinase with 54% amino acid sequence identity to T7K (Fig. 5b), recently demonstrated that this kinase could bind to DNA and inactivate two DNA-targeting defence systems by phosphorylation at critical residues (Dnd, QatABCD)^23,57^. Involvement in deactivation for three further systems with clear links to DNA interaction was also provided. Although the reported extent of phosphorylation for this kinase was about 20-fold lower than that of T7K, our data on conserved hyperphosphorylation of close JSS1 homologues suggest that this kinase is as promiscuous as T7K. Exploring defence systems more systematically in the future will surely yield more T7K-sensitive systems. However, it is safe to say that this sensitivity will not be universal for diverse members of a defence system class, as members may evolve to avoid critical Ser/Thr/Tyr residues on their surface, or even adapt to use phosphorylation as a trigger, as indicated by one natural isolate that defended better when T7K was present.

Overall, T7K phosphorylation is one of the first general phage-encoded anti-defence mechanisms that act independently of the bacterial defence system’s catalytic activity. Another general mechanism comes from Ocr, encoded adjacently to T7K (gp0.3) in T7. Ocr blocks R/M systems^66^ and BREX^67^ through DNA mimicry^68^. Such general anti-defence mechanisms are bound to be more common, given the size of phage genomes and diversity of bacterial immunity. PTM enzymes, with their prevalence in (pro)phages and rapid responses^16^, are good candidates for future general anti-defence mechanisms. Whether they are as hyperpromiscuous as T7K remains to be explored, and sensitive proteomics methods will be key for resolving such questions.

## Methods

### Bacterial strains

All strains for the experiments were grown in lysogeny broth (LB) Lennox and the required antibiotics and incubated at 37 °C with shaking. *E. coli* K-12 (BW25113)^72^ was used for most biological analyses, and the BL21-AI or NEB5α strains were used for protein purification and cloning, respectively. The use of the ECOREF^53^ strain collection is described in detail in later sections. When culturing strains transformed with plasmids, the following antibiotic selection was used: 100 μg ml^−1^ spectinomycin, 25 µg ml^−1^ chloramphenicol and 100 μg ml^−1^ kanamycin.

### Phages

Phage T7 WT was acquired from DSMZ, T7Δ*0.7::cmk* (referred to as Δ*0.7* throughout the manuscript) was a gift from U. Qimron^73^. Our phage stocks were checked for the presence of the correct T7 genotype with the primers TAGCCAACACACTGAACGCT and CACCCGCAGATGACCTGTAA for T7 WT and TAGCCAACACACTGAACGCT and GCCAAAAGGCGCTCAAAGTT for T7Δ*0.7::cmk*. Moreover, we confirmed that the known deletion mutant H1 (ref. ^74^) did not occur in our stocks (using the primers TAGCCAACACACTGAACGCT, CACCCGCAGATGACCTGTAA and GCCAAAAGGCGCTCAAAGTT). Two mutants of T7, the catalytic dead G76F^35^ and a truncated kinase mutant ΔSO (kinase domain only, residues 1–242), were generated using in vitro methods for genome engineering and cell-free phage rebooting using proprietary methods developed by Invitris. Phages from the BASEL collection^59^ (Bas64, Bas65, Bas66(T3K12), Bas67 and Bas68) were a gift from A. Harms. Note that the infection curves of our Bas67 stock behaved significantly differently to the other BASEL *Autographiviridae*, so we excluded this phage from further analyses. Phage P1*vir* was single-plaque purified from a Typas laboratory stock. Annotated genomes were downloaded from the NCBI using the GenBank accessions as reported in ref. ^59^ and NC_047864.1 (reference genome used to represent T3/Bas66(T3K12)) and NC_005856.1 (reference genome used to represent P1*vir*).

Phages were propagated in the *E. coli* K-12 (BW25113) strain and cultured in LB, 5 mM CaCl_2_ and 10 mM MgCl_2_ at 37 °C with shaking. Lysates of overnight cultures infected with phage stocks were prepared by adding chloroform, vortexing and centrifuging (4,000*g*, 4 °C, 5 min) to remove debris. The lysate was transferred to fresh tubes, additional chloroform was added, and the samples were vortexed and centrifuged again. Clarified lysates were stored at 4 °C. Lysate titres were determined in duplicates using the double-agar overlay method^75^.

### Plasmids

For the study of recombinant T7K, the regions encompassing the N-terminal PK domain (residues 1–242) and the C-terminal SO domain of T7K (residues 243–359) were PCR-amplified from T7 gDNA using Q5 High-Fidelity DNA Polymerase (NEB), with primers designed to introduce a 6×His-TEV N-terminal fusion and carrying overhangs to plasmid pET28a.

Almost all plasmids used in the T7K screen (Supplementary Table 5) are in the pTU175 backbone, which carries the low-copy-number origin of replication pSC101, except for the Borvo and Retron-Eco8 defence systems, which were obtained from the lab of Rotem Sorek and kept in the pSG1-vector, expressed from their native promoter and with a p15a origin of replication^48,52^, and CmdTAC (PD-T4-9) defence system, which was obtained from the laboratory of M. Laub, and was kept on its original plasmid pCD1–PD-T4-9 under its native promoter^76^. Plasmids containing Retron-Eco1, Retron-Eco9 and Retron-Sen2 have been described previously^33^. For Retron-Eco3, Retron-Eco6 and Retron-Eco10, the retrons were amplified with their native promoters (500 bp upstream of the retron start) from strains from the ECOREF *E. coli* natural isolate library^53^ or from strain SC402 (Retron-Eco3), and cloned into pTU175. For the toxin–antitoxin system TacAT^77^, the protein-coding sequence was amplified from *Salmonella typhimurium* LT2 and cloned into the pTU175 backbone under the arabinose-inducible pBAD promoter. The HEPN-MNT^78^ and the ApeA^49^ defence systems were cloned into the pTU175 vector under the arabinose-inducible pBAD promoter (gift from I. Songailiene, V. Šikšnys laboratory). The DarTG1 defence system was cloned into the pTU175 vector under the arabinose-inducible pBAD promoter by M. LeRoux^79^.

Phosphomimetic mutant constructs of RcaT (in Retron-Eco9) and DarT (in DarTG1) were generated using the Q5 site-directed mutagenesis kit (NEB), using the primers described in Supplementary Table 6. Plasmid edits were confirmed by Sanger sequencing and PacBio whole-plasmid sequencing.

### Infection sampling (single timepoint)

To measure proteome and phosphoproteome changes in several phage infections (WT T7, T7 mutants, BASEL phages) of *E. coli* K-12 (BW25113), a single colony was picked and grown overnight in LB, 10 mM MgCl_2_ and 5 mM CaCl_2_ and, the next morning, was back diluted 1:1,000 into fresh LB, 10 mM MgCl_2_ and 5 mM CaCl_2_ (300 ml). The bacteria were cultured at 37 °C with shaking, and their optical density at 600 nm (OD_600_) was monitored over time until an OD_600_ of around 0.5 (1 cm pathlength). Phage stocks were added to a final MOI of 5 and infections were allowed to proceed at 37 °C with shaking. Uninfected samples were immediately placed on ice. To collect at the indicated timepoints, the flasks were immediately transferred to a pre-cooled centrifuge and spun (4,000*g*, 5 min, 4 °C). For each sample, the supernatant was removed and the cell pellet was resuspended in 1 ml ice-cold PBS, and then centrifuged once again (10,000*g*, 1 min, 4 °C). The supernatant was aspirated and the pellet was flash-frozen in liquid nitrogen. In total, three biological replicates were performed for each phage infection state (three independent colonies prepared on different days). The samples were then prepared for phosphoenrichment (see below), and matched protein expression measurements of the unenriched peptide digests also collected using data-independent analysis without further offline peptide fractionation (see below).

### Infection time-course sampling

To measure proteome and phosphoproteome changes in WT T7 and Δ*0.7* phage infections of *E. coli* K-12 (BW25113), samples were prepared as above but the culture was then split into six independent conical flasks and phage stock (either WT or Δ*0.7*, or no stock as the uninfected control) added to a final MOI of 5 and infections were allowed to proceed at 37 °C with shaking. Uninfected samples were immediately placed on ice, or for infections collected at 1, 5 or 10 min after infection for WT T7, or at 5 and 10 min for Δ*0.7*, the flasks were immediately transferred to a precooled centrifuge and spun (4,000*g*, 5 min, 4 °C). In total, three biological replicates were performed (time courses from three independent colonies prepared on different days).

To compare phosphoproteome changes in WT T7 and ΔSO phage infections, samples from each phage were collected as described above at 10 min after infection (to measure the effect of the loss of the SO domain at saturation), or at 2, 3 or 4 min after infection (to measure the effect before saturation), with three biological replicates performed for both experiments (time courses from three independent colonies prepared on different days for each experiment). To increase temporal resolution for the pre-saturation experiment, we collected the cell suspension directly into Falcon tubes prefilled with ice cold PBS.

### MS sample preparation (time course)

The lysis buffer was composed of 4 M guanidinium isothiocyanate, 50 mM HEPES, 5 mM TCEP, 20 mM chloroacetamide, 1% *N*-lauroylsarcosine, 5% isoamyl alcohol and 40% acetonitrile, with the pH adjusted to 8.5 using 10 M NaOH. For cell lysis, a buffer volume of approximately five times the cell pellet volume was added. The samples were homogenized by pipetting, incubated on a shaker at room temperature for 1 h, and centrifuged at 16,000*g* for 10 min to remove cell debris and nucleic acid aggregates. The protein concentration was determined by tryptophan fluorescence, as previously described^80^. The samples were transferred to MultiScreenHTS-HV 96-well filter plates with 0.45 µm PVDF membranes (Merck Millipore), and ice-cold acetonitrile was added to each well to induce protein precipitation, achieving a final concentration of 80% acetonitrile. After a 10 min incubation, the samples were centrifuged, and the supernatant was discarded. The protein precipitates were then washed twice with 200 µl of 80% acetonitrile and twice with 200 µl of 70% ethanol, with each wash followed by centrifugation at 1,000*g* for 2 min.

Next, a digestion buffer containing 50 mM triethylammonium bicarbonate buffer and trypsin (TPCK-treated, Thermo Fisher Scientific) was added to the protein precipitates. The trypsin-to-protein ratio was set to 1:25 (w/w), with a maximum final protein concentration of 10 µg µl^−1^. Tryptic digestion was performed overnight at room temperature with mild shaking (600 rpm). After digestion, the samples were dried using a vacuum concentrator.

For the parallel proteomics measurements, the full digest was TMT labelled (see below) and then subjected to offline high-pH fractionation (see below). For phosphoproteomics measurements phosphopeptide enrichment was performed (see below).

### Phosphopeptide enrichment

For phosphoproteomics studies (single infection timepoint for several phages, time courses comparing WT T7 with Δ*0.7* or with ΔSO infections, Retron-Eco9 and DarTG1 infections), phosphopeptide enrichment was performed^81^. Lyophilized peptides were resuspended in a loading and washing buffer composed of 80% acetonitrile and 0.07% TFA, sonicated and centrifuged at 16,000*g*. Phosphopeptide enrichment was carried out on the supernatant as described previously^82^ using the KingFisher Apex robot (Thermo Fisher Scientific) with 25 µl of Fe-NTA MagBeads (PureCube) per sample. After five washes with buffer A, phosphopeptides were eluted with 100 µl of 0.2% diethylamine in 50% acetonitrile, followed by lyophilisation.

The enriched phosphoproteomic time-course samples were TMT labelled (see below) and then subjected to offline porous graphitic carbon (PGC) fractionation. The enriched single timepoint infections and Retron-Eco9 and DarTG1 infection samples were not TMT-multiplexed and were subjected to MS analysis without further peptide fractionation.

### MS sample preparation (defence systems)

For phosphoproteomics studies, *E. coli* K-12 were transformed with plasmids encoding Retron-Eco9 (native expression) or DarTG1 (arabinose-inducible). Overnight cultures were started in LB, 10 mM MgCl_2_, 5 mM CaCl_2_, 100 μg ml^−1^ spectinomycin, and then from these a 50 ml day culture (1:2,000 back dilution) was started the next morning in fresh LB, 10 mM MgCl_2_, 5 mM CaCl_2_, 100 μg ml^−1^ spectinomycin. DarTG1 culture medium had 0.2% (w/v) arabinose for the induction of DarTG1 expression. Cells were grown until an OD_600_ of around 0.05 (Retron-Eco9) or about 0.6 (DarTG1) (pathlength = 1 cm), infected with WT T7 at an MOI of 5 and then collected after 5 min of infection, as described above in the ‘MS sample preparation (time course)’ and ‘Phosphopeptide enrichment’ sections. Two independent biological replicates were performed.

For experiments assessing phosphomutant protein stability (through abundance), cells transformed with either Retron-Eco9 or DarTG1 constructs (or their corresponding empty plasmids) were cultured as described above, but collected (without phage infection) at an OD_600_ of around 0.05 (Retron-Eco9) or 2 (DarTG1). Cells were collected by centrifugation (5 min, 4,000*g*, 4 °C), washed once with ice-cold PBS, centrifuged again and then resuspended in 5 pellet volumes of 2% (w/v) SDS. The samples were boiled for 5 min, and then subjected to five rounds of freeze–thaw before being processed for MS analysis (sample cleanup, proteolytic digestion, TMT-multiplexing, high pH fractionation; see the sections below). Three independent biological replicates were performed.

### MS sample clean-up and protein digestion

MS sample preparation and measurements were performed as described previously^83^. Protein digestion was performed using a modified SP3 protocol^84^. The samples were diluted to a final volume of 20 μl with 0.5% SDS and mixed with a paramagnetic bead slurry (10 μg beads (Sera-Mag Speed beads, Thermo Fisher Scientific) in 40 μl ethanol). The mixture was incubated at room temperature with shaking for 15 min. The beads bound to the proteins were washed 4 times with 70% ethanol. Proteins on beads were reduced, alkylated and digested using 0.2 μg trypsin, 0.2 μg LysC, 1.7 mM TCEP and 5 mM chloroacetamide in 100 mM HEPES, pH 8. After overnight incubation, the peptides were eluted from the beads, dried under vacuum, reconstituted in 10 μl of water and labelled with TMTpro, 18-plex reagents and dissolved in acetonitrile at 1:15 (peptide:TMT weight ratio) for 1 h at room temperature. The labelling reaction was quenched with 4 μl of 5% hydroxylamine and the conditions were pooled together. The pooled sample was desalted with solid-phase extraction after acidification with 0.1% formic acid. The samples were loaded onto the Waters OASIS HLB μelution plate (30 μm), washed twice with 0.05% formic acid and finally eluted in 100 μl of 80% acetonitrile containing 0.05% formic acid. The desalted peptides were dried under vacuum and dissolved for analysis using liquid chromatography coupled with MS (LC–MS).

### Expression and purification (6×His-TEV-PK)

The plasmid pET28a-6×His-TEV-PK was transformed into *E. coli* BL21-AI. Cells were grown at 37 °C in 1.2 l of ZYM-5052, supplemented with 100 μg ml^−1^ kanamycin (Sigma-Aldrich) and 0.05% arabinose (Sigma-Aldrich), and cultures were shifted to 30 °C when the OD_600_ reached around 0.8–0.9. Protein overproduction was carried out for a further 18 h. Cells were collected in the Beckman-Coulter JLA 8.1000 rotor (5,000 rpm, 30 min, 4 °C) and pellets were resuspended in 45 ml of binding buffer (20 mM Tris pH 7.5, 500 mM NaCl, 5 mM imidazole) supplemented with EDTA-free protease inhibitor (Roche), 100 μg ml^−1^ DNase I (Sigma-Aldrich) and 0.5 mg ml^−1^ lysozyme (Sigma-Aldrich). Cells were lysed in a C3 Emulsiflex high-pressure homogenizer (Avestin) and insoluble material was pelleted (80,000*g*, 20 min, 4 °C). The supernatant was passed through a 0.2 μm PVDF filter (Merck Millipore) and applied to 1 ml of pre-equilibrated Ni-NTA agarose beads (Qiagen) on a gravity-flow column. Beads were washed with 10 ml of binding buffer, then 10 ml of wash buffer (20 mM Tris pH 7.5, 500 mM NaCl, 60 mM imidazole) and the protein eluted in 5 ml of elution buffer (20 mM Tris pH 7.5, 500 mM NaCl, 1 M imidazole), collecting 500 μl aliquots. The fractions with the highest purity and yield were pooled and dialysed twice against 2 l of dialysis buffer (20 mM Tris pH 7.5, 500 mM NaCl). The protein was stored at −80 °C in 30% glycerol.

### Expression and purification (6×His-TEV-SO)

The plasmid pET28a-6×His-TEV-SO was transformed into *E. coli* BL21-AI. Cells were grown at 37 °C in 1.2 l of ZYM-5052 (ref. ^85^), supplemented with 100 μg ml^−1^ kanamycin (Sigma-Aldrich) and 0.05% arabinose (Sigma-Aldrich), and cultures were shifted to 30 °C when the OD_600_ reached about 0.8–0.9 (pathlength = 1 cm). Protein overproduction was carried out for a further 18 h. Cells were collected in the Beckman-Coulter JLA 8.1000 rotor (5,000 rpm, 30 min, 4 °C) and the pellets resuspended in 45 ml of lysis buffer (50 mM Tris pH 7.5, 5 mM EDTA) supplemented with EDTA-free protease inhibitor (Roche), 100 μg ml^−1^ DNase I (Sigma-Aldrich) and 0.5 mg ml^−1^ lysozyme (Sigma-Aldrich). Cells were lysed in a C3 Emulsiflex high-pressure homogenizer (Avestin) and insoluble material pelleted (80,000*g*, 20 min, 4 °C). The pellet was resuspended in 30 ml of membrane solubilization buffer (50 mM Tris pH 7.5, 2% Triton X-100) and samples were pelleted again (80,000*g*, 20 min, 4 °C). The pellet was then resuspended in high salt buffer (50 mM Tris pH 7.5, 1 M NaCl, 2% Triton X-100) and ultracentrifuged again (80,000*g*, 20 min, 4 °C). Inclusion bodies were resuspended in high-urea buffer (50 mM Tris pH 8.0, 7.6 M urea) and let solubilize at 4 °C for 18 h with gentle shaking. Insoluble material was removed by ultracentrifugation (80,000*g*, 20 min, 4 °C) and urea-solubilized protein was stored at −80 °C.

### T7K protein solubility (western blotting)

Lysates from cells expressing either 6×His-TEV-PK or 6×His-TEV-SO were mixed with SDS–PAGE loading buffer either directly after lysis (whole cell) or after removal of insoluble material by centrifugation at 16,000*g* and 4 °C for 45 min (soluble). The samples were boiled at 100 °C for 10 min, then loaded on 15% polyacrylamide for SDS–PAGE. Proteins were transferred to PVDF and membranes blocked in Tris-buffered saline buffer containing 0.5% Tween-20 (TBST) and 5% skimmed milk for 1 h at room temperature. Membranes were incubated with anti-His (Invitrogen, MA1-21315) at 1:2,000 overnight at 4 °C, then washed three times in TBST before incubation with goat anti-mouse HRP (Cytiva, NA931) at 1:10,000 for 1 h at room temperature. Membranes were washed again three times in TBST, then imaged on a iBright1500 system (Thermo Fisher Scientific).

### In vitro phosphorylation assay

To remove autoinhibitory phosphosites on T7K and enable the study of its activity in vitro, dephosphorylation of purified T7K kinase domain was performed. The reaction was performed in a total volume of 4.5 ml. Purified 6×His-TEV-PK was supplemented with 10 mM MnCl_2_ and 4,000 U of lambda protein phosphatase (NEB) and incubated at 30 °C. After 3 h, another 4,000 U of lambda protein phosphatase were added, and the sample was incubated at 30 °C for another 3 h. To remove lambda protein phosphatase, the mixture was applied to 500 μl of pre-equilibrated Ni-NTA agarose beads (Qiagen) on a gravity flow column. The beads were washed with 20 ml of binding buffer (20 mM Tris pH 7.5, 500 mM NaCl, 5 mM imidazole), then 6×His-TEV-PK eluted in 5 ml of elution buffer (20 mM Tris pH 7.5, 500 mM NaCl, 1 M imidazole), collecting 500 μl aliquots. The fractions with the highest purity and yield were pooled and dialysed twice against 2 l of dialysis buffer (20 mM Tris pH 7.5, 500 mM NaCl, 1 mM DTT, 1 mM EDTA). The protein was stored at −80 °C in 30% glycerol at a final concentration of 0.5 mg ml^−1^.

For the in vitro phosphorylation assay, 500 μg of *E. coli* K-12 tryptic digest per replicate were resuspended in a buffer containing 50 mM HEPES pH 7.5, 100 mM NaCl, 5 mM Mg-ATP and 1 mM TCEP. Subsequently, 5 μg of the purified (and dephosphorylated) kinase domain was added, and the reaction was incubated at 37 °C for 4 h. The peptides were next lyophilized before phosphopeptide enrichment and label-free LC–MS/MS. In total, three biological replicates were performed.

### Phosphorylation stoichiometry estimation

Tryptic peptides (40 µg for each of the three replicates) corresponding to either *E. coli* infected with T7 WT or Δ*0.7* (at *T* = 10 min after infection, three biological replicates) were subjected to alkaline phosphatase treatment (5 U of FastAP per sample; Thermo Fisher Scientific) in a volume of 50 µl of phosphatase buffer, at 37 °C for 20 h. As a control, the same amount of tryptic peptides was incubated in the same conditions without phosphatase. After incubation, the samples were lyophilized and desalted using t-C18 Sep-Pak columns (Waters) before TMT labelling and offline PGC fractionation.

### 6×His-TEV-SO in vitro refolding assay

gDNA was isolated from 50 ml of *E. coli* BW25113 overnight culture using the DNeasy Blood & Tissue Kit (Qiagen) and concentrated to about 0.8 mg ml^−1^ in water in a vacuum concentrator. Total *E. coli* RNA was obtained from Invitrogen. Refolding reactions were assembled as follows: gDNA (80 μg) or an equal volume of water (no DNA control) were mixed with 40 μg of urea-solubilized 6×His-TEV-SO in 25 mM Tris pH 8.0, 3.8 M urea, in a total volume of 200 μl. For refolding experiments in the presence of RNA, total RNA (40 µg) or an equal volume of water (no RNA control) were mixed with 20 μg of urea-solubilized 6×His-TEV-SO in 25 mM Tris pH 8.0, 4 M urea, 100 U ml^−1^ SUPERase RNase inhibitor (Invitrogen) in a total volume of 200 μl. Mixtures were transferred to 6–8 kDa molecular weight cut-off D-Tube Dialyzer Mini tubes (Merck-Millipore) and dialysed for 18 h at 4 °C with gentle stirring in refolding buffer (25 mM Bis-Tris pH 6.6, 500 mM NaCl, 1 mM MgCl_2_). Dialysed samples were collected into clean 1.5 ml microcentrifuge tubes and samples containing RNA supplemented with another 100 U ml^−1^ RNase inhibitor. Aliquots (15 μl) of total material (input fraction) were collected after dialysis, then the rest of the sample was centrifuged (16,000*g*, 45 min, 4 °C) and the supernatants (soluble fraction) were collected. Benzonase-treated controls were prepared as follows: 50 μl aliquots of soluble fraction from samples containing gDNA were supplemented with 125 U of benzonase (Merck-Millipore) and 10 mM MgCl_2_ and incubated at 37 °C for 4 h, then insoluble material was removed by centrifugation (16,000*g*, 45 min, 4 °C) and the supernatants were collected. In total, samples across three biological replicates were collected.

To assess integrity of RNA after overnight dialysis in refolding experiments, 20 µl of input and soluble fractions from samples containing RNA were mixed with 6× Purple loading dye (New England Biolabs), denatured at 70 °C for 3 min and immediately placed on ice, then resolved on 1.5% agarose in TAE buffer at 80 V. Aliquots of original RNA stock samples were included as integrity controls. RNA was imaged under UV light after ethidium bromide staining.

MS sample preparation of all fractions (input, soluble, benzonase-treated controls), including TMT-multiplexing was prepared as described above (Sample clean-up and proteolytic digestion). As there was some evidence of remnant protease from the genomic extraction kit, for protein digestion, the ‘nonspecific’ setting was used as protease with an allowance of maximum 2 missed cleavages requiring a minimum peptide length of 7 amino acids.

### TMT labelling

For the full proteome measurements, 10 µg of peptides per condition was labelled while, for the phosphoproteome, the totality of enriched phosphopeptides was used. Peptides were resuspended in 10 µl of 100 mM HEPES (pH 8.5), and 4 µl of TMTPro reagent at a concentration of 20 µg µl^−1^ in acetonitrile was added. The labelling reaction was allowed to proceed for 1 h at room temperature, and was then quenched by the addition of 5 µl of 5% hydroxylamine for 15 min. Labelled peptides belonging to the same experiment were subsequently pooled and lyophilized.

Before fractionation, the phosphopeptides were resuspended in 50 µl of 10% TFA and desalted using in-house C18 stage tips^86^ packed with 1 mg of ReproSil-Pur 120 C18-AQ 5 µm material (Dr. Maisch) above a C18 resin plug (AttractSPE Disks Bio, C18, Affinisep); in the case of the full proteome samples, the peptides were desalted using the Sep-Pak t-C18 column (Waters).

### PGC-LC offline peptide fractionation

The samples were resuspended in 18 µl of buffer A (0.05% TFA in MS-grade water supplemented with 2% acetonitrile), with an injection volume set to 16 µl. Peptide separation was conducted using a Hypercarb column (100 mm length, 1.0 mm inner diameter, 3 µm particle size, Thermo Fisher Scientific) at 50 °C, with a flow rate of 75 µl min^−1^, on an Ultimate 3000 Liquid Chromatography system (Thermo Fisher Scientific). After a 1 min post-injection delay, a linear gradient separation was applied, increasing from 13% buffer B (0.05% TFA in acetonitrile) to 42% buffer B over 47 and 95 min for the phosphoproteome and full proteome samples, respectively. This was followed by an increase to 80% buffer B within 5 min. The column was then washed with 80% buffer B for 5 min before re-equilibration with 100% buffer A for another 5 min. Fractions were collected from 4.5 to 52.5 min and from 4.5 to 100.5 min at 2-min intervals, yielding 24 fractions and 48 fractions for the phosphoproteome and full proteome samples, respectively. These were then pooled into 12 or 24 fractions by combining each fraction with its corresponding *n* + 12 and *n* + 24 fraction for the phosphoproteome and full-proteome samples, respectively. After pooling, the fractions were dried using a vacuum concentrator prior to LC–MS/MS analysis.

### High-pH offline peptide fractionation

The samples were fractionated using a reversed-phase C18 system running under high-pH conditions, as described previously^83^. In brief, this consisted of an 85 min gradient (mobile phase A: 20 mM ammonium formate (pH 10); mobile phase B: acetonitrile) at a flow rate of 0.1 ml min^−1^, starting at 0% B, followed by a linear increase to 35% B from 2 min to 60 min, with a subsequent increase to 85% B up to 62 min, held at 85% B until 68 min, followed by a linear decrease to 0% B up to 70 min, and finally held at this level until the end of the run. Fractions were collected every 2 min from 12 min to 70 min, and fractions were concatenated to make a total of 6 fractions (Retron-Eco9, DarTG1 expression analysis) or 24 fractions (phage infection time-course expression analysis) by combining each fraction with its corresponding *n* + 6 and *n* + 24 fraction.

### Data-dependent LC–MS/MS

Before injection, all of the samples were resuspended in a loading buffer composed of 1% TFA, 50 mM citric acid and 2% acetonitrile in MS-grade water. LC separation was performed on the UltiMate 3000 RSLCnano system (Thermo Fisher Scientific). Peptides were initially trapped on a cartridge (Precolumn: C18 PepMap 100, 5 μm, 300 μm inner diameter × 5 mm, 100 Å) before being separated on an analytical column (Waters nanoEase HSS C18 T3, 75 μm × 25 cm, 1.8 μm, 100 Å). Solvent A was composed of 0.1% formic acid with 3% DMSO in LC–MS-grade water, while solvent B contained 0.1% formic acid with 3% DMSO in LC–MS-grade acetonitrile. Peptides were loaded onto the trapping cartridge at 30 μl min^−1^ with solvent A for 5 min, then eluted at a constant flow rate of 300 nl min^−1^ using a linear gradient of buffer B, followed by an increase to 40% buffer B, a wash at 80% buffer B for 4 min and re-equilibration to the initial conditions. The linear gradient corresponded to an increase from 5% to 25% B in the case of label-free peptides, and from 7% to 27% B in the case of TMT-labelled peptides.

The LC system was coupled to a Fusion Lumos Tribrid, an Exploris 480 mass or a Q-Exactive Plus mass spectrometer (Thermo Fisher Scientific), operated in positive-ion mode. The mass spectrometers were operated in data-dependent acquisition mode with a maximum duty cycle time of 3 s or a top 20 method, selecting precursors with charge states 2–7 and a minimum intensity of 2 × 10^5^ for subsequent HCD fragmentation. Peptide isolation was performed using the quadrupole with 0.7 *m*/*z* or 1.4 *m*/*z* isolation windows in the case of TMT-labelled and label-free samples, respectively. MS/MS spectra were acquired in profile mode using the Orbitrap, with a maximum injection time of 100 ms and an AGC target of 1 × 10^5^ charges.

### Data-independent LC–MS/MS

For protein expression analysis of BASEL phage infection samples, peptides were resuspended in a loading buffer composed of 1% TFA and 2% acetonitrile in MS-grade water and then separated using the Vanquish Neo UHPLC system (Thermo Fisher Scientific) operated in trap-and-elute mode. The LC systems was equipped with a trapping cartridge (Precolumn; PepMap Neo C18, 5 μm, 300 μm inner diameter × 5 mm, 100 Å) and an analytical column (Ionopticks AUR3-25075C18-XT, 25 cm × 75 μm inner diameter, 1.7 μm C18). Solvent A was 0.1% formic acid supplemented with 3% DMSO in LC–MS-grade water and solvent B was 0.1% formic acid supplemented with 3% DMSO in LC–MS-grade acetonitrile. Peptides were loaded onto the trapping cartridge and separated on the analytical column using a linear gradient of 5–26% B (flow rate of 300 nl min^−1^) for 29.7 min, followed by an increase to 40% B within 3 min before washing at 85% B for 5 min and re-equilibration to initial conditions (total MS acquisition time of 40 min). The LC system was coupled to an Orbitrap Astral mass spectrometer (Thermo Fisher Scientific) operated in data-independent acquisition mode. The instrument was operated in positive-ion mode with a spray voltage of 1.8 kV and a capillary temperature of 280 °C. Full-scan MS spectra were acquired in the Orbitrap at a resolution of 240,000 with a mass range of 430–680 *m*/*z*, an AGC target of 5 × 10^6^ charges and a maximum injection time of 5 ms. DIA spectra were acquired in the Astral mass analyser with 2 *m*/*z* windows between 430 and 680 *m*/*z*. MS2 scan range was set to 150–2,000 *m*/*z*, the normalized collision energy to 25 and the default charge state to 2+. The normalized AGC target was set to 500% and the maximum injection time to 5 ms.

### LC–MS/MS raw data processing

For all proteomic analyses, a combined protein sequence database was constructed consisting of the Swissprot *E. coli* strain K-12 database (4,401 entries, UP000000625) supplemented with a common protein contaminants database (118 entries). For analyses involving phage T7 infections, this was further supplemented with the Swissprot phage T7 proteome (UP000000840, 57 entries). For analyses involving the mutant phages, the FASTA including the full length T7K sequence was used. For analyses involving *E. coli* transformed with either defence-system-containing plasmid, the database was also supplemented with the proteins encoded on each plasmid—DarTG1 (5 entries) and Retron-Eco9 (5 entries). For the SO-domain solubility analysis, the Swissprot *E. coli* strain K-12 database was supplemented with contaminants and additionally supplemented with the sequence for the recombinant T7K SO-domain protein. For experiments involving BASEL phages (Bas64, Bas65, Bas68 and P1*vir*), the *E. coli* database was supplemented with protein FASTAs downloaded from the NCBI (GenBank: MZ501110.1, MZ501081.1, MZ501078.1, MZ501055.1 and NC_005856.1, respectively). The proteome produced by the T3 reference genome GenBank NC_047864.1 was used for Bas66(T3K12).

For data-dependent analyses, raw files were converted to mzmL files using MSConvert (v3.0.24260-9bf2070) from Proteowizard^87^, using peak picking and keeping the 1,000 most intense peaks per spectrum. Files were then searched using MSFragger (v.4.0)^88^ in Fragpipe (v.21) using the appropriate database for the given phage infection (see above). The default MSFragger phospho (for label-free) or TMT16-phospho (for proteomics and phosphoproteomics) workflow was used, with a few modifications: oxidation on methionine (maximum 2 occurrences), phosphorylation on Ser/Thr/Tyr (maximum three occurrences) and, for TMT analyses, peptide N-terminal TMT16 labelling (maximum 1 occurrence) set as variable modifications. A total of up to five variable modifications was allowed per peptide. Lysine TMT16 labelling (for TMT analyses) and cysteine carbamidomethylation were set as fixed modifications. Percolator (v.3.6.4)^89^ was used for PSM validation and PTMProphet (TPP v.6.3.2)^90^ was used to determine site localization. An FDR cut-off of 1% was used. For the TMT quantification, Philosopher (v.5.1.0)^91^ was used to extract MS1 and TMT intensities.

For data-independent analyses, raw files were directly inputted into DIA-NN^92^ (v.2.1.0). Samples were processed based on the identity of the infecting phage (uninfected, WT T7 or BASEL phage). Spectral libraries were generated directly from the inputted FASTA using the deep learning-based spectra, retention time and ion mobility prediction mode, using trypsin/P protease specificity. DIA-NN was run with defaults, except using a precursor and fragment ion *m*/*z* range of 430–680 and 200–1,800, respectively, up to two missed cleavages allowed. Furthermore, cysteine carbamidomethylation (+57.021464 Da, UniMod:4) was set as a fixed modification, and methionine oxidation (+15.994915 Da, UniMod:35) as a variable modification, with up to two variable modifications per peptide. Quantification was performed using Legacy (direct) mode summarizing at the protein group level and using the --ids-to-names mode, with the match-between-runs feature. The protein group quantification matrices generated by DIA-NN were used for downstream statistical analyses.

### MS data analysis and statistics (general)

For the summarization of information at the phosphosite level, the highest localization score for each site across redundant peptide spectrum matches (PSMs) was considered. Class I phosphosites were those that were uniquely mapping (only one protein in the FASTA database) with a PTMProphet^90^ phosphorylation localization probability of ≥0.75. The summarization of quantitative information at the (phospho)peptide levels (for phosphoproteomics and stoichiometry experiments) was performed by summing the TMT intensities of all the PSMs assigned to that specific (phospho)peptide.

For TMT analyses, the PSM tables produced by FragPipe were filtered to retain PSMs with a purity value of >0.5 with full quantification (the signal in all used TMT channels). For all experiments, contaminant proteins were filtered out. For phosphoproteomics experiments, all phospho-modified peptides were considered; for the stoichiometry experiment, only unique peptides were considered; for protein expression analysis, only proteins with at least one unique peptide were considered. Differential expression analysis was performed using the summed TMT reporter ion intensities (summarized at the modified peptide level) for phosphoproteomics and stoichiometry experiments, or using the protein intensities from MSFragger output for other protein expression experiments. Vsn-normalization^93^ and differential expression analysis were used as implemented in the limma package (v.3.58.1)^94^ in R (v.4.3.3). For the time courses, and for WT versus ΔSO 10 min infection TMT experiments, vsn-normalization was performed grouped by sample type (phage genotype + timepoint) for phosphoproteomics; or, for proteomics, grouped by the protein’s species origin (host proteins normalized together over all times, phage proteins normalized by timepoint). Limma was used to compare samples to the uninfected timepoints, to compare genotypes at matched timepoints, and/or to compare timepoints across a specific phage infection. The model design included the phage genotype and timepoint status for phosphoproteomics, or considering sample type (phage genotype + timepoint) and replicate status for proteomics. The linear model was fitted using limma’s lmFit() function and a moderated *t*-statistic was computed using limma’s eBayes() function. *P* values were adjusted for multiple testing using the Benjamini–Hochberg procedure implemented in limma. Proteins were considered to be statistically significantly changed if they had adjusted *P* < 0.05 and absolute log_2_[FC] > 1.5. Phosphopeptides were considered to be statistically changed if they had adjusted *P* < 0.05 and an absolute log_2_[FC] > 1.

For the Retron-Eco9 and DarTG1 construct expression experiments, vsn-normalization was performed without grouping of samples/proteins. For the T7K SO domain solubility analysis, no vsn-normalization was performed. In both experiments, statistical significance was assessed using unpaired *t*-tests, using the stat_compare_means() function of the ggpubr R package.

For analysis of protein expression in BASEL phages from DIA data, protein groups with at least one unique peptide were considered, with only successful quantifications (no zero or NA values) considered further for normalization and plotting. vsn-normalization was performed without grouping of samples/proteins.

For the analysis of the TMT-multiplexed WT versus ΔSO kinetics (2, 3 and 4 min after infection) experiment, no normalization was performed. TMT reporter ion intensities were summed at the phosphopeptide (modified peptide) level for peptides with quantification in all three replicates of a given phage infection.

### MS analysis and statistics (stoichiometry)

For the stoichiometry analysis, only peptides that spanned residues previously found to be phosphorylated in the time-course phosphoproteomics experiment were considered. The following PSMs were not considered for downstream analysis: (1) PSMs without complete TMT labelling (that is, without peptide N-terminal labelling); (2) PSMs modified with Ser/Thr/Tyr phosphorylation or M oxidation variable modifications; (3) PSMs mapping to proteins identified as differentially abundant in the time-course protein expression analysis (adjusted *P *< 0.05, FC of <0.5 or >1.5 at *T* = 10 min, WT T7 versus Δ*0.7*).

Two limma analyses were performed, and both analysed summed peptide intensities that were vsn-normalized without any sample/protein grouping. The first was performed to calculate and assess the quality of the phosphatase/control ratios for each phage genotype (WT T7, Δ*0.7*) and each of three biological replicates by inputting summed vsn-normalized TMT reporter intensities and comparing phosphatase versus control samples. It considered the sample (WT–phosphatase, WT–control, Δ*0.7*–phosphatase, Δ*0.7*–control) and replicate status in the model design. Differential analysis with limma was performed as described above. We considered some peptides to have poor phosphatase/control ratios when they were found in this analysis to be either significantly downregulated (limma, adjusted *P* < 0.05, log_2_[FC] < 0) or the log_2_[FC] was low (log_2_[FC] < −0.2, no significance requirement). These were excluded from the subsequent analysis. The estimated stoichiometry value for each genotype was calculated from these ratios using the following calculation: $100\times (1-1/{2}^{{\log }_{2}[{\rm{FC}}]})$. The T7K-mediated stoichiometry was calculated by subtracting the estimated stoichiometry of the Δ*0.7* from the WT T7 sample.

The second limma analysis was performed to determine differential stoichiometry, that is, stoichiometry deposited by the T7K. It was assessed by inputting manually calculated phosphatase/control ratios for each genotype and replicate for the peptides that had quality phosphatase/control ratios (see above) in both samples (*T* = 10 WT and *T* = 10 Δ*0.7*). These were manually calculated as: 2^summed vsn-normalized phosphatase intensity^/2^summed vsn-normalized control intensity^. The limma model was constructed to consider the phage genotype (WT T7 or Δ*0.7*) and replicate status. Peptides were considered to be of high differential stoichiometry if they had adjusted *P *< 0.05 and log_2_[FC] > 1.5.

### GO analysis

To categorize phosphoproteins, goslim_prokaryotic_ribbon annotations were downloaded from QuickGO^95^ and annotated onto proteins. Proteins were assigned one of four representative GOSlim terms using the following ordered scheme: (1) transcription, translation (GOSlim terms: ribosome biogenesis, translation, regulation of DNA-templated transcription, DNA-templated transcription); (2) metabolic processes (GOSlim terms: metabolic process, primary metabolic process, lipid metabolic process, generation of precursor metabolites and energy, amino acid metabolic process, DNA metabolic process, small molecule biosynthetic process); (3) other (GOSlim terms: cell wall organization or biogenesis, detoxification, protein folding, transport, response to stimulus); or (4) unannotated, if it could not be mapped to a GOSlim.

GO-term analysis was performed using the STRINGdb R package (v.2.14.3)^96^, initialized with the following settings: species = 511145, version = 12.0, score threshold = 400. The get_enrichment() command (with defaults) was applied. For the non-phosphorylated proteome subset, host proteins (≥1 unique peptide identified in the parallel proteomics measurement) that were not found to have any uniquely mapping T7K-dependent phosphopeptides were compared to all detected host proteins (≥1 uniquely mapped peptide identified in the parallel proteomics measurement) as background. For the stoichiometry analysis, host proteins mapping to high differential stoichiometry peptides were considered, comparing to all detected host proteins (≥1 uniquely mapped peptide identified in the same experiment) as background.

### Analyses with published phosphoproteomes

Phosphosites for *E. coli* (K-12) were downloaded from the dbPSP2.0 (ref. ^97^) in June 2024. Mass spectra from several previously published phosphoproteomics studies of *E. coli* (PRIDE submissions PXD008369, PXD008921 and PXD008289) were reanalysed as described above, with IDs harmonized using the same FASTA database.

The fraction of phosphorylated Ser, Thr and Tyr residues in different datasets was calculated per phosphorylated protein as the number of phosphorylated Ser, Thr and Tyr residues divided by the total number of Ser, Thr and Tyr residues present in the protein’s sequence. For the T7 dataset, either (1) all detected sites from the TMT time-course; or (2) only sites sensitive to T7K were considered. The phosphorylation fraction was also calculated for the human proteome based on (1) all phosphosites reported in the PhosphoSitePlus database^98^; (2) a comprehensive collection human phosphosites measured by MS curated in a recent stringent meta-analysis^69^; and (3) a single experimental dataset (using the same phosphoenrichment protocol as performed in this study) comprising phosphosites that were sensitive to EGF stimulation in HEK293F cells^70^. All distributions were compared to the distribution of T7-sensitive set of proteins using unpaired two-sample Wilcoxon signed-rank tests, implemented in the stat_compare_means() function of the ggpubr R package.

### T7K homology search

To find putative homologues to the T7 kinase, we queried both viral and bacterial sequence databases using HMMs built from PHROG 2828 (ref. ^99^) (encompassing UniProt entry P00513 of T7K) augmented with the JSS1 kinase sequence. To distinguish the T7K kinase-domain and the shutoff-domain, we subset the PHROG 2828 alignment (generated using mafft^100^, v.7.505) into the parts corresponding to both domains (based on known domain architecture in T7 kinase), built an HMM for each and searched with both HMMs individually. To conduct the search, we used pyhmmer^101^ (v.0.10.15) and searched the databases against representative sequences of BFVD^61^ as well as against genomes from BASEL phage collection^59^ (to which we added the JSS1 genome) and all genomes from progenomes3 (ref. ^62^). All HMM hits were filtered using an *E* value of 10^−10^. We built a phylogeny (using fasttree^102^, v.2.2) on a multiple-sequence alignment of all identified T7K kinase domains. We did not consider hits coming from metagenome-assembled genomes as well as low-quality assemblies containing >400 contigs. To account for likely viral contamination, we reassigned contigs originally coming from progenomes3 to be of viral origin if they were longer than 40 kb and contained no bacterial marker gene (bacterial marker genes were identified using gtdb-tk^103^ (v.2.1.1). Out of 32 total contigs with putative T7K homologues found in progenomes3, we re-assigned 27 contigs as likely viral contaminants in this way. The remaining 5 contigs are all exceptionally long and contain multiple bacterial marker genes (Extended Data Fig. 9c). For those contigs, we ran PHASTEST^104^ (through the web API) to pinpoint probable prophage regions. We ran InterProScan (through the online interface) to identify protein domains on all hits. AlphaFold3 (ref. ^105^) predictions for several clade representatives with non-canonical domain configurations were also generated using the AlphaFold webserver.

### Sequence motif analysis

For the analysis of T7K kinase substrate activity motifs, the ten amino acid residues surrounding each side of a phosphorylated residue (only from sites with PTMProphet localization score ≥ 0.75) were extracted and used to generate sequence logo plots using the ggseqlogo R package (v.0.2)^106^.

For the analysis of kinase active site motifs sequence variability, we extracted residues corresponding to the active sites of both viral and human kinases and subsequently generated sequence logo plots using the ggseqlogo R package (v.0.2)^106^ of the active sites and surrounding residues. Human kinase domain sequences for DFG motif generation were obtained by downloading the human kinome domains curated in the KinBase resource (http://kinase.com/web/current/kinbase/).

### Protein structure analysis

Phage T7 protein structures were predicted by subjecting the Swissprot phage T7 proteome (UP000000840) sequences to AlphaFold3^105^ modelling through the AlphaFold Server. The PyMOL Molecular Graphics System (v.3.03 Schrödinger) was used to calculate the relative per-residue solvent accessible surface area on the top ranked model of each protein using the get_sasa_relative command. ChimeraX (v.1.10)^107^ was used to globally align (matchmaker) the AlphaFold3 prediction of T7K + Mg^2+^ + ATP with the Protein Data Bank (PDB) experimental structures 1FIN^108^ and 1KV2 (ref. ^109^).

FoldSeek^110^ structural homology searches were performed through the FoldSeek webserver, using the top-ranked AlphaFold3 (ref. ^105^) models generated for the full-length T7K and for T7K kinase-domain only (residues 1–242), and searching all available databases in 3Di/AA mode.

For the analysis of the T7K AlphaFold3 protein structure, PyMOL was first used to convert from .cif to .pdb format. This .pdb file was submitted to the GraphBind server^111^, with all ligand-specific options (including DNA and RNA) considered. Residues reported to be DNA or RNA binding are those that passed GraphBind’s internal binary thresholds.

### Plasmid-based infection assays

To test a wide range of phage MOIs, serial dilutions of phage lysates (10 μl final volume) were added in round-bottom 96-well plates. We based MOI calculations on the assumption that an optical density of 1 corresponds to 1 × 10^9^ bacterial cells. Plates containing the phages were pre-warmed at 37 °C for 1 h before adding the cells. *E. coli* BW25113 strains carrying plasmids encoding defence systems (with native promoters or with the inducible pBAD promoter; Supplementary Table 5), or a control plasmid, were diluted 1:2,000 from overnight cultures and grown at 37 °C. Strains with a plasmid with a native promoter were grown until OD_600_ = 0.05 (1 cm pathlength); strains with a plasmid with an inducible pBAD promoter were grown until OD_600_ = 0.6 (1 cm pathlength) in arabinose-containing medium (0.2% l-arabinose) as the pBAD promoter is catabolite repressed in LB during exponential growth. Note that, for Retron-Eco9 (Fig. 3a and Extended Data Fig. 5e), infection at OD_600_ = 0.05 (1 cm pathlength) led to inconsistent results, possibly because the culture entered stationary phase before the phage managed to collapse the bacterial population. We therefore started to dilute the cells 1:10 when they reached OD_600_ = 0.05, infecting the cells at OD_600_ = 0.005, which resulted in consistent phenotypes and effect sizes. Then, 90 μl of culture was added to the 96-well plates that contained phages and the plates were sealed with breathable membranes (Breathe-Easy). We kept several LB-filled wells in each experiment for background subtraction and to check for potential contamination. The plates were then incubated with constant shaking at 37 °C in a microplate reader, and the OD_600_ was measured every 10 min to acquire growth curves (either in a Biotek Synergy HT or Biotek synergy H1). Note that the OD_600_ values presented throughout all figures are from a microplate reader and were not pathlength corrected.

### Infection assays with natural isolates

The natural isolate strain collection (536 unique strains, 558 strains in total; Supplementary Table 7) is organized on 6 different 96-well plates. We reorganized some strains so that each plate contained at least two empty wells to check for cross-contamination and to use for background subtraction. The whole collection was screened in four batches.

Phage lysate stocks were prepared to account for final MOIs of 5, 0.01, 0.001, 0.0001 and 0, determined for the reference strain BW25113, and assuming that an OD_600_ of 1 corresponds to 10^9^ bacterial cells. We diluted the overnight cultures 1:2,000 and grew the strains at 37 °C and 800 rpm in deep 96-well plates until the first strains reached OD_600_ = 0.1 (1 cm pathlength). Note that the strains grow at different rates, which makes it impossible to infect them all at the exact same OD_600_ = 0.1 (1 cm pathlength). Thus, the exact phage to bacteria ratio will vary for each strain, but we tried to account for that by applying a range of MOIs. We then pipetted 90 µl of the bacterial cultures into 9 different round-bottomed 96-well plates, and added 10 µl of phage lysates on top (a single MOI per plate). Plates were sealed with breathable membranes (Breathe-Easy) and incubated without lids in a humidity-saturated incubator (Cytomat 2, Thermo Fisher Scientific) with continuous shaking. Plates were measured every 45 min in a Filtermax F5 multimode plate reader (Molecular Devices).

Out of the 558 strains, the uninfected control (MOI = 0) did not grow for six strains (ECOR-70, MG1655, ATCC 8739, S17 plate1, DH1, DH1 (E381)), so they were removed from the analysis. Strains with sequences that did not match the expected genotype were also removed from the analysis (all strains with a/b/c/x as a suffix in their name; Supplementary Table 7); note that 17 strains are present in replicates (same strain names) within the collection and we added the plate number behind the name to distinguish these in the analysis (Supplementary Fig. 3). One strain (NR-2654) appeared twice on the same plate, so we added _1 and _2 to the name, respectively. Three strains (IAI01, S17 and NR-2654) had replicates with different strain IDs (NT numbers), and we counted them only once among the unique strains. All replicates fell in the same categories (uninfected, infected without evidence of defence, infected with evidence of defence), except for three (MP1, ECOR-09, ECOR-10). For these strains, we removed the replicate from the uninfected category, as MP1 was validated from an independent glycerol stock to be infected (Supplementary Fig. 3), and ECOR-09 and ECOR-10 were slowly growing in the replicate that showed infection (Supplementary Fig. 3), so the other replicate was probably contaminated. For the analysis, we therefore assessed 513 unique strains (529 strains in total). Four other strains (BL21(DE3), DH5α, ECOR-09 plate 5, ECOR-10 plate 5) grew very slowly and the integration time for the AUC calculation (see below) was therefore increased from 6 to 10 h (Supplementary Fig. 3). The whole collection was screened in one replicate, and all 22 strains showing infection and evidence of defence (Supplementary Fig. 3c) were repeated from an independent glycerol stock and with higher MOI resolution (validations) in at least two biological replicates, that is, starting from two independent colonies on a plate (Supplementary Fig. 4). For the phage infection experiments comparing the different T7K mutants (Δ*0.7*, ΔSO, G76F) in natural isolate strains, we used an expanded MOI range (5, 1, 0.5, 10^−1^, 10^−2^, 10^−3^, 10^−4^, 10^−5^, 10^−6^, 10^−7^, 10^−8^ and 0).

### Infection assay data processing

All data from plate reader assays were processed and plotted using MATLAB R2017b (The MathWorks). The data were background-subtracted with one background value (minimum measured value across each plate and timepoints) and plotted over time. For the natural-isolate screen, we subtracted the background at each timepoint with the minimum measured value across all plates of a batch at the timepoint. The AUC was determined under the linear growth curve from 0 h to 6 h of measurement using the function trapz. AUCs were normalized to the AUC of the uninfected control (MOI = 0) of the same strain (normalized AUC). To estimate the effect size for a biological replicate of a strain, we interpolated the MOI at an AUC of 0.5 (0.75 for ECOR-63 in Supplementary Fig. 5, as curves did not readily reach 0.5) on the log_10_ scale for both T7 WT and mutants, using the MATLAB function interp1. We then took the inverse logarithm base 10 of the difference (Δ*0.7* minus WT). As the effect size strongly depends on well-matched phage titres of T7 WT and Δ*0.7*, we considered only a mean effect size of >5-fold or <0.2-fold as a hit (Fig. 4b and Extended Data Fig. 7a).

### Bacterial isolate genome analyses

Genomic nucleotide sequences for the majority of the *E. coli* natural isolates were downloaded from the ECOREF^53^ resource’s website (https://evocellnet.github.io/ecoref/). The genomes of several strains (IAI09, IAI41, Perfeito-Clone-6, H10407, HM-341) were additionally sequenced using long-read sequencing. Overnight cultures of strains grown at 37 °C with shaking, were used for DNA extraction with the DNA mini-prep kit (ZymoBIOMICS). For library preparation, 1.2 μg of DNA for each sample was taken into fragmentation using Megaruptor 3.0 (Diagenode) with a speed of 31 and a final volume of 100 μl. Then, 1× clean-up with SMRTBell beads (PacBio) was performed. All recovered material was used as an input for library preparation using SMRTBell 3.0 chemistry (PacBio) and following the manufacturer’s instructions. Final libraries were quantified by Qubit and Femto Pulse system was used to assess the fragment distribution. Libraries were then pooled equimolarly and loaded in the Sequel IIe instrument at 100 pM with 30 h video time. The resulting PacBio reads were assembled into high-quality, complete genomes using Flye^112^ (v.2.9-b1768) using standard parameters.

To annotate known bacterial anti-phage defence systems in natural isolate strain genomes, nucleotide sequences were either (1) inputted directly into the PADLOC Python program (v.2.0.0, using the default settings)^113^ using PADLOC-DB v.2.0.0; or (2) first translated into a protein FASTA file using the Prodigal Python package (v.2.6.3, using the default settings)^114^, and then inputted into the DefenseFinder python package (v.1.2.2, using the default settings)^6^ using DefenseFinder models (v.1.2.3)^115^ and CasFinder models (v.3.1.0)^116^.

### Reporting summary

Further information on research design is available in the Nature Portfolio Reporting Summary linked to this article.

## Online content

Any methods, additional references, Nature Portfolio reporting summaries, source data, extended data, supplementary information, acknowledgements, peer review information; details of author contributions and competing interests; and statements of data and code availability are available at https://doi.org/10.1038/s41586-026-10934-5.

## Supplementary information

Supplementary Information Supplementary Figs. 1–5.

Reporting Summary

Supplementary Tables Supplementary Table 1: phosphoproteome of phage infections (single timepoint). Supplementary Table 2: analyses of T7K structural and sequence homology. Supplementary Table 3: quantitative phosphoproteome and proteome analysis of phage infection (timecourse). Supplementary Table 4: estimation of phosphorylation stoichiometry. Supplementary Table 5: plasmid-based screen of T7K interaction with phage defence systems. Supplementary Table 6: Retron-Eco9 and DarTG1 analyses. Supplementary Table 7: natural isolate library screen details and defence system characteristics. Supplementary Table 8: analyses of diverse phage kinases

Peer Review File

## Source data

Source Data Fig. 3

Source Data Fig. 4

Source Data Extended Data Fig. 5

Source Data Extended Data Fig. 7

## Data Availability

All raw MS files, search parameters and search outputs were deposited to the ProteomeXchange Consortium through the PRIDE partner repository under dataset identifier PXD058408. Previously published phosphoproteomics datasets reanalysed here are available from PRIDE (PXD008369, PXD008921 and PXD008289). Human phosphoproteome comparison datasets are available from their original publications^69,70^. This study also used the following public databases: PhosphoSitePlus (https://www.phosphosite.org), dbPSP 2.0 (http://dbpsp.biocuckoo.cn/), PHROG (https://phrogs.lmge.uca.fr/), BFVD (https://bfvd.steineggerlab.workers.dev/), proGenomes3 (https://progenomes.embl.de/), Protein Data Bank (1FIN and 1KV2), KinBase (http://kinase.com/web/current/kinbase/), QuickGO (https://www.ebi.ac.uk/QuickGO/), STRING v.12.0 (https://string-db.org), the Genome Taxonomy Database (https://gtdb.ecogenomic.org), PADLOC-DB v.2.0.0 (https://github.com/padlocbio/padloc-db), DefenseFinder models v.1.2.3 and CasFinder models v.3.1.0 (https://github.com/mdmparis/defense-finder-models). The assembled long-read sequencing genomes have been uploaded to the ECOREF website (https://evocellnet.github.io/ecoref/download/) and are also available at Figshare^117^ (https://figshare.com/articles/dataset/E_coli_genomes/28007045). AlphaFold3 structural predictions of the phage T7 proteome, T7K kinase domains and selected homologues are available at Zenodo^118^ (https://doi.org/10.5281/zenodo.14497210). Raw and source data for all infection assays are provided alongside custom code at Zenodo (https://doi.org/10.5281/zenodo.20727692)^119^, and raw gel images for Extended Data Fig. 4b,c are provided in Supplementary Fig. 1. Source data are provided with this paper.

## Code availability

Code used for bioinformatic analyses throughout the Article is downloadable at Zenodo (https://doi.org/10.5281/zenodo.20727692)^119^.
