# Supplementary material for: Pervasive phosphorylation by phage T7 kinase disarms bacterial defences
Source: Nature. 2026 Aug 19;658(8135):454–64. doi: 10.1038/s41586-026-10934-5 (PMC13645617; doi:10.1038/s41586-026-10934-5)
Supplement: Supplementary file 1 [file 41586_2026_10934_MOESM1_ESM.pdf]

nature portfolio

https://doi.org/10.1038/s41586-026-10934-5

Supplementary information

Pervasive phosphorylation by phage T7 kinase disarms bacterial defences

Supplementary Figures

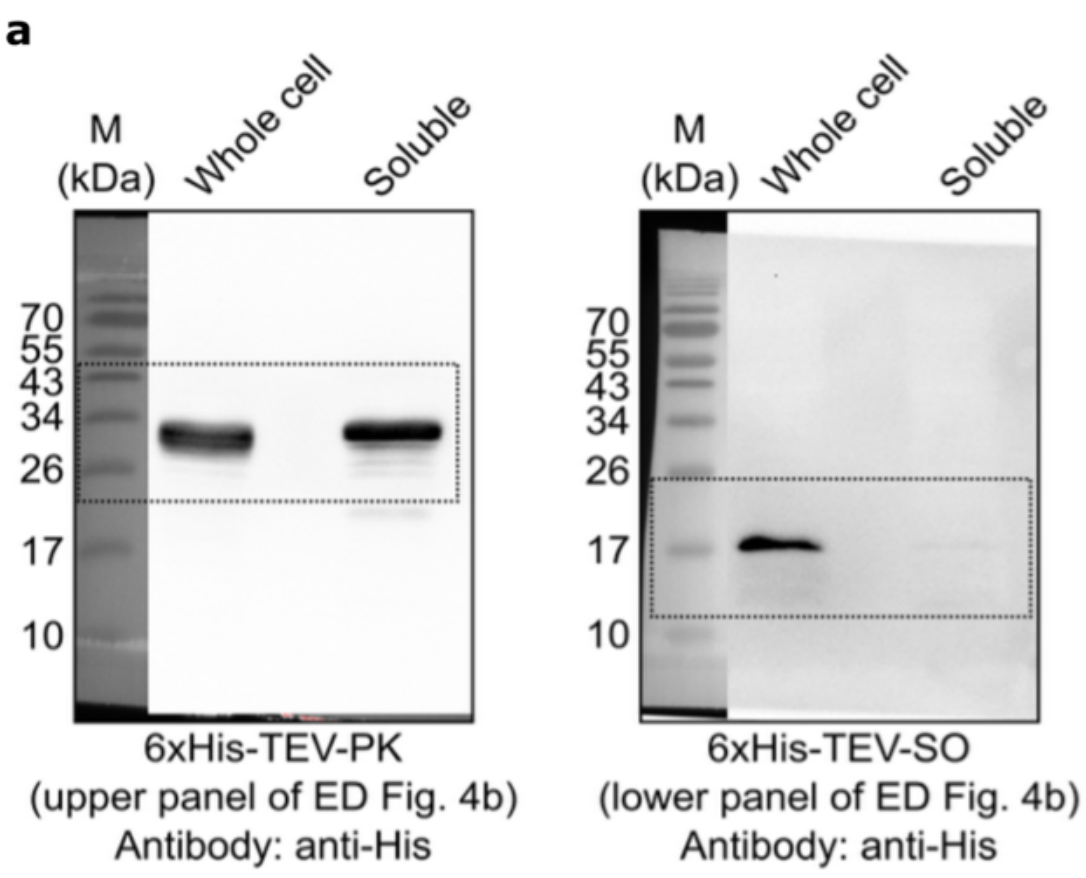

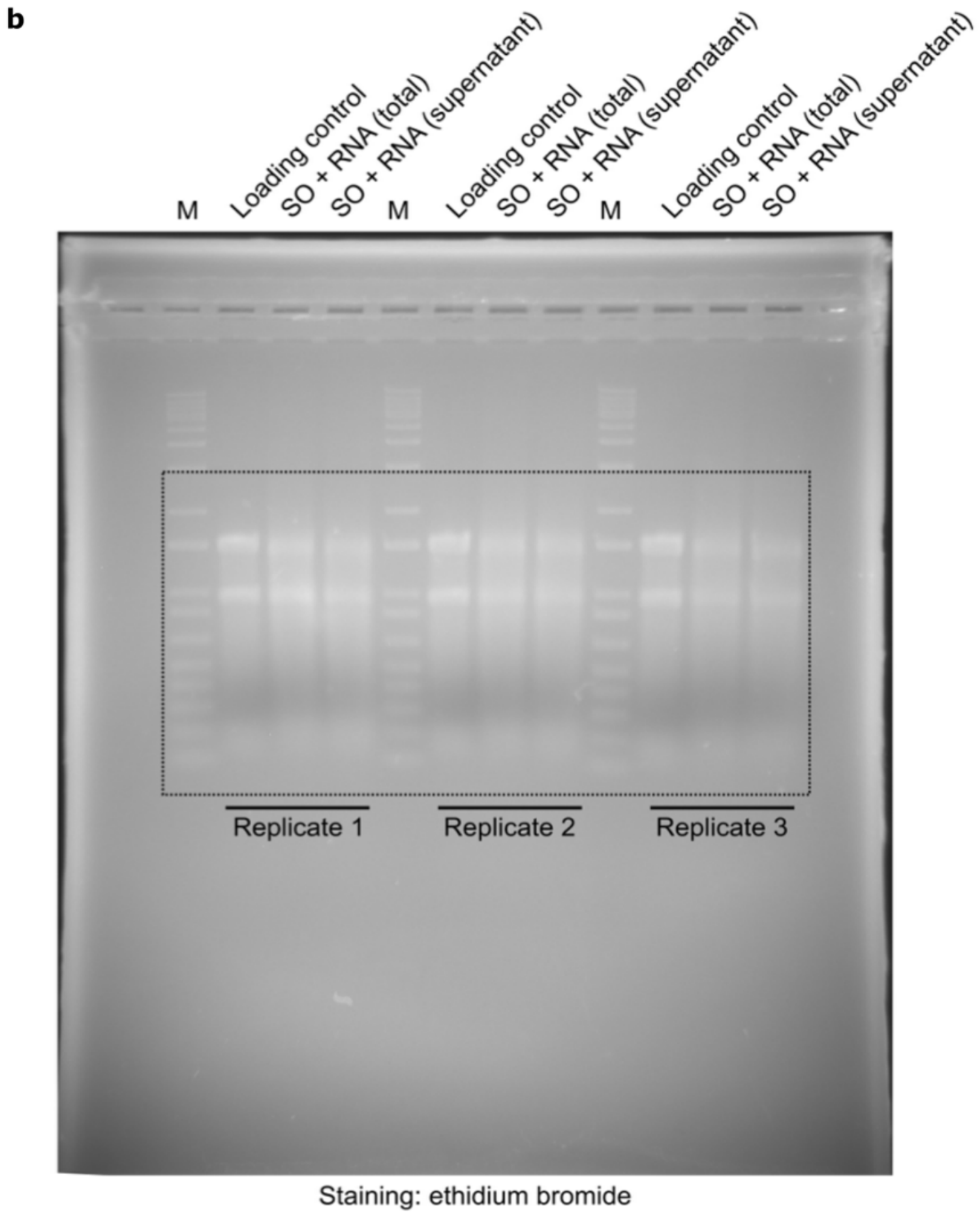

Supplementary Figure 1: Raw gel images for protein purification and refolding assay control experiments. (a) Labelled raw uncut images supporting Extended Data Figure 4b (purification of T7K domains) and **(b)** supporting Extended Data Figure 4c (refolding assay controls). Dotted areas denote blot and gel images used in the manuscript.

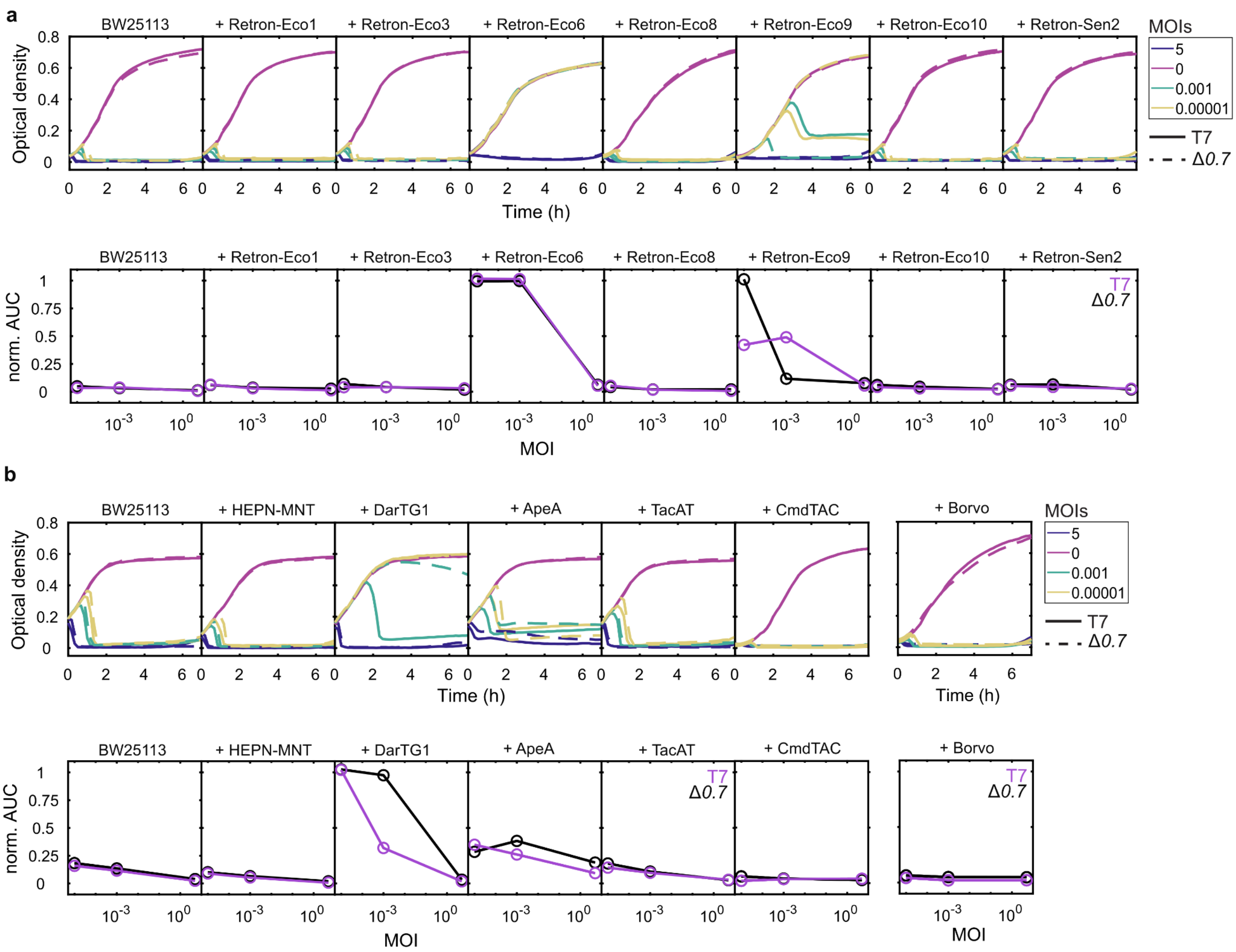

Supplementary Figure 2: Primary data for plasmid-based infection assay screen. (a) Defense against T7 WT and Δ0.7 by msDNA-containing retrons under native promoters. Upper panel: bacterial growth of E. coli BW25113 +/- the indicated defense systems over the course of T7 infection measured by optical density (600 nm) in a microplate reader. Lower panel: Normalized area under the curve (norm. AUC), normalized to the uninfected sample for all growth curves of the control BW25113 +/- defense systems infected with T7 (purple lines) or Δ0.7 (black lines) at the indicated MOIs (empty circles). Data is the same as in the upper panel. **(b)** Defense against T7 by DNA/RNA-binding systems (left-side plots) and one CHAT-protease domain-containing system (Borvo; right-side plot). Upper and lower panels as in (a).

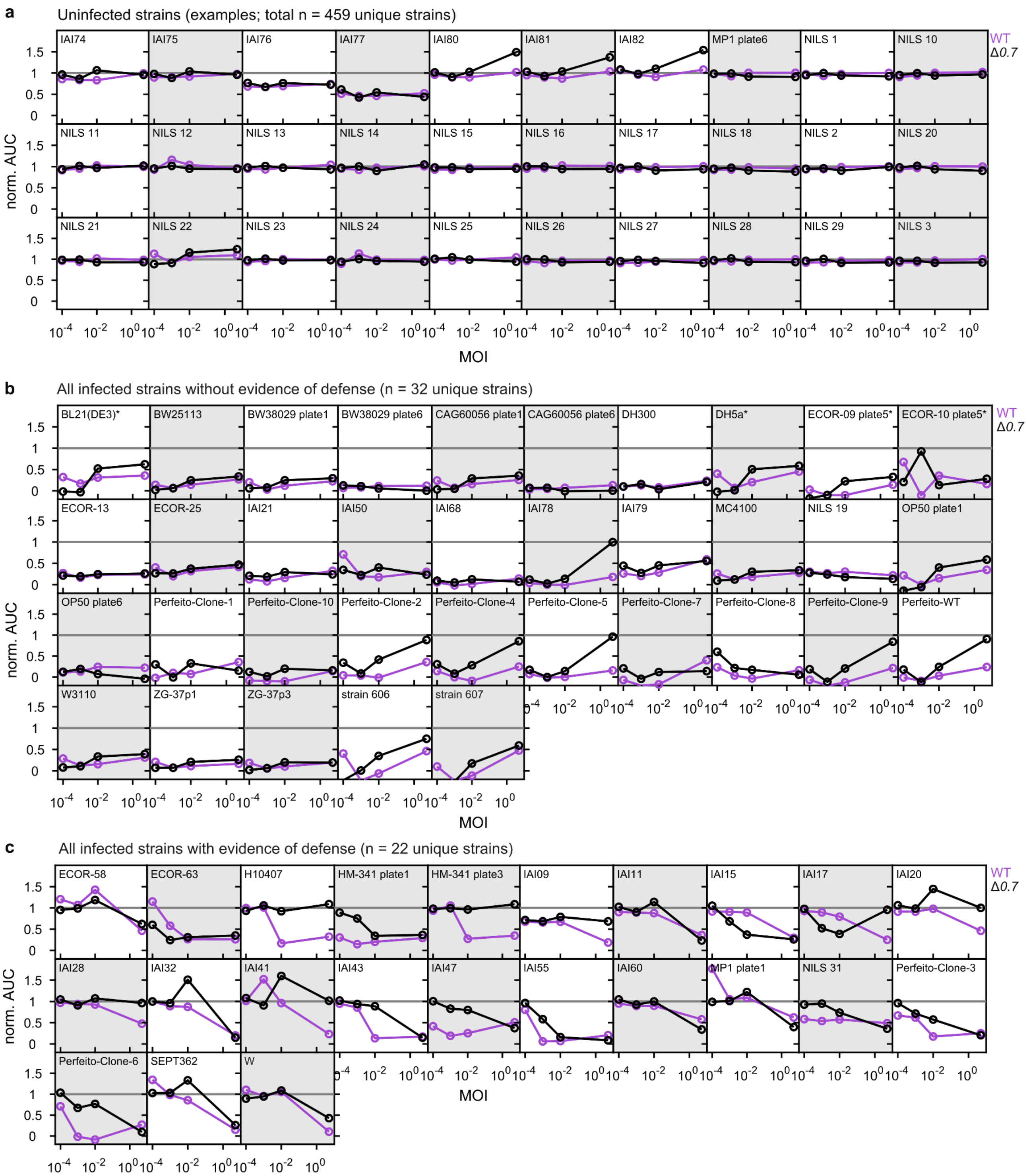

Supplementary Figure 3: Primary data for natural isolate strain infection assay screen. Natural isolates are grouped into three categories: uninfected, infected without evidence of defense, and infected with evidence of defense. **(a)** Normalized area under the curve (norm. AUC) over multiplicity of infection (MOI) plot for 30 example strains that were not infected by T7 WT or Δ*0.7*. Their norm. AUC is constant over MOIs with a value of close to 1, meaning they grow as well as the uninfected control at all MOIs. Values may be consistently but slightly below 1 (e.g. strain IAI77) due to a technical reading issue in the uninfected control. **(b)** Norm. AUC over MOI plot for all strains that got infected by T7 WT or Δ*0.7*, but did not show any evidence of defense. Their norm. AUC is constant over all MOIs with a value close to 0, meaning that they do not grow at all, except in the uninfected control. For strains labeled with *, the integration time for norm. AUC was increased from 6h to 10 h, as they were growing very slowly. Values clearly above 0 are either due to technical problems or the development of resistance at later time-points. Strains present in replicates in the library have the same background color which alternates between white and grey. **(c)** Norm. AUC over MOI plot for all strains that were infected by T7 WT or Δ*0.7*, and had evidence of defense. Their norm. AUC decreases

with increasing MOI, in either T7 WT or Δ*0.7*, meaning that they grew like the uninfected control at lower MOIs, but collapsed at the higher MOIs. All strains were infected at 5 MOIs (5, 10^{-2}, 10^{-3}, 10^{-4}, and 0) with T7 WT and Δ*0.7*. Strains present in replicates in the library have the same background color which alternates between white and grey.

a Validations of strains with evidence of defense in screen, kept for effect size analysis

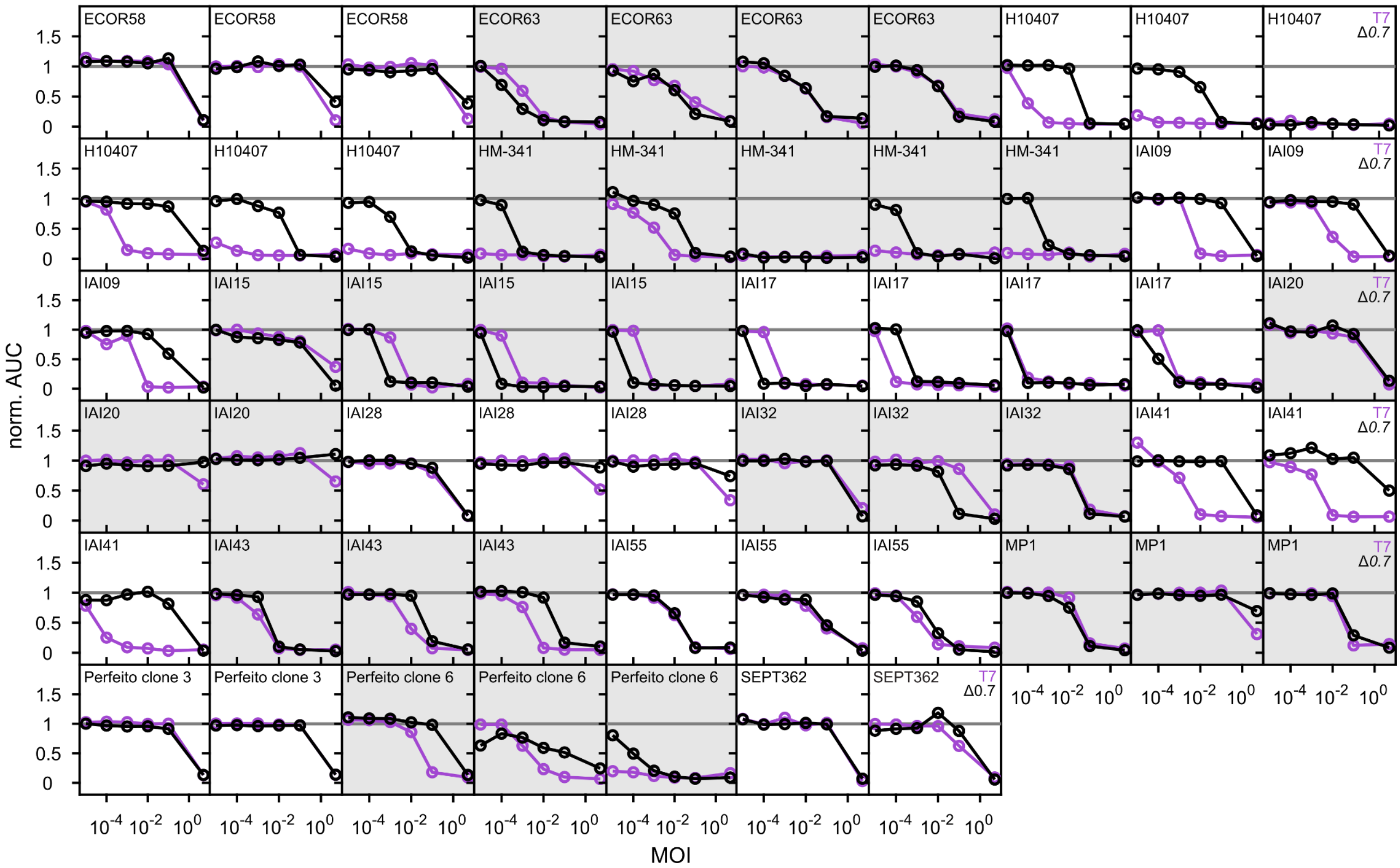

b Validations of strains with evidence of defense in screen, removed from effect size analysis

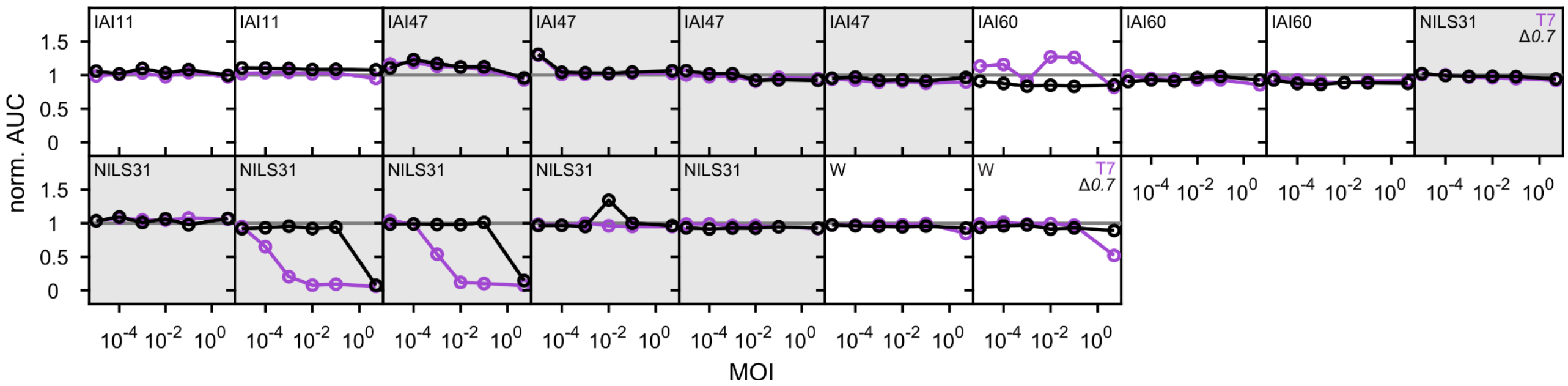

Supplementary Figure 4: Primary data for validations of natural isolate strains that were infected by T7, but showed evidence of defense in the screen. (a) Normalized area under the curve (norm. AUC) over multiplicity of infection (MOI) plots for strains that were infected by T7, but showed evidence of defense (Supplementary Fig. 3c). All strains originate from individual glycerol stocks and were infected at seven MOIs (5, 10^{-1}, 10^{-2}, 10^{-3}, 10^{-4}, 10^{-5}, and 0) with T7 WT and Δ*0.7* in at least two biological replicates. Replicates have the same background color which alternates between white and grey. **(b)** Norm. AUC over MOI plots for strains that got infected with evidence of defense (Supplementary Fig. 3c) and were removed from the downstream effect size analysis, as we could not reproducibly validate the evidence of defense seen in the screen. Strains were treated and data are represented as described in (a).

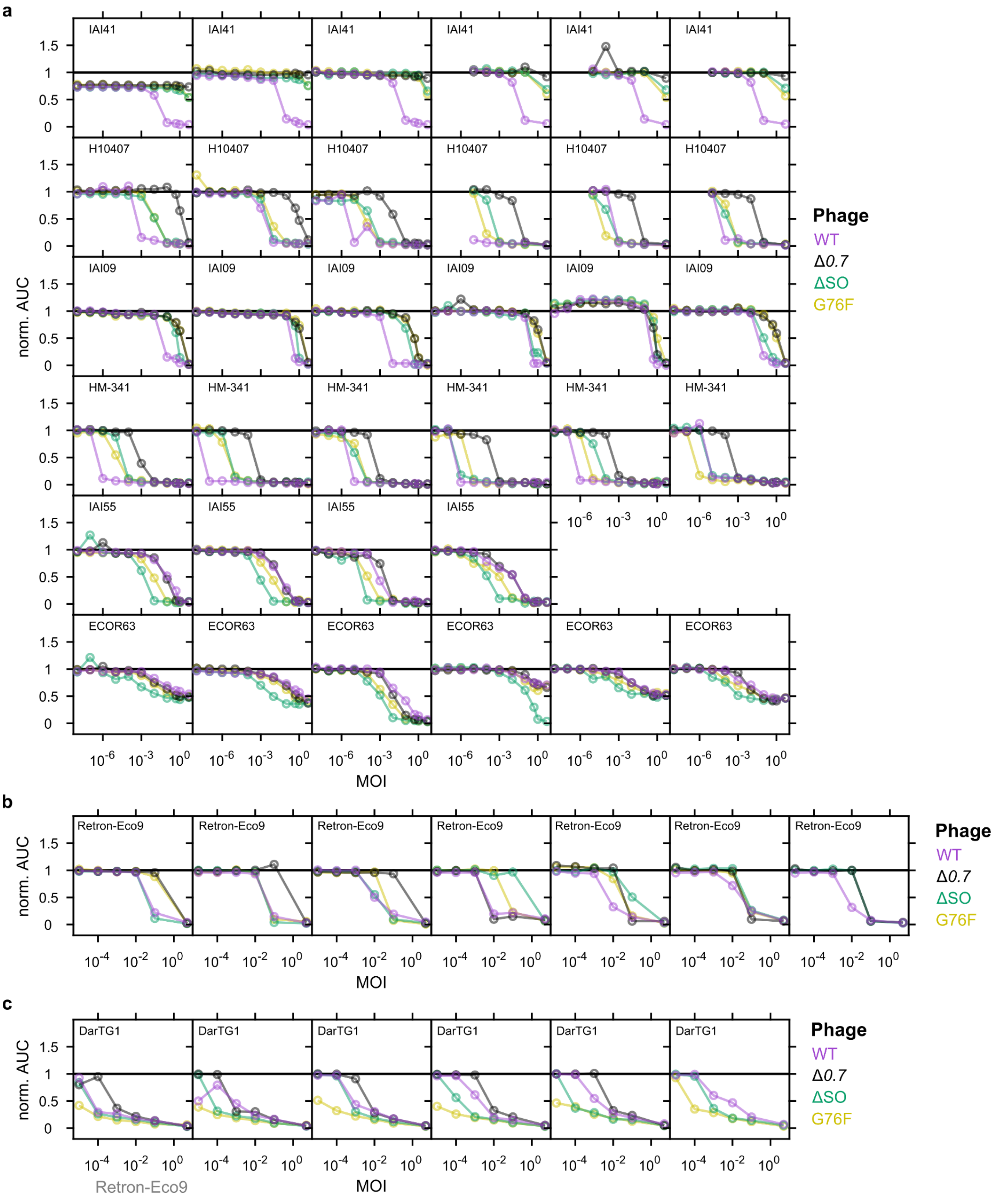

Supplementary Figure 5: Primary data for infection assays using the expanded panel of T7K mutant phages. (a) Normalized area under the curve (norm. AUC) over multiplicity of infection (MOI) plots for four natural isolate strains with evidence of strong sensitivity to T7K (IAI41, H10407, IAI09 and HM341), and two control T7K-neutral strains (ECOR-63 and IAI55) challenged with four phages (WT T7 - purple, Δ*0.7* - black, ΔSO - green, and G76F - yellow). Strains were infected at expanded MOI range (5, 1, 0.5, 10^{-1}, 10^{-2}, 10^{-3}, 10^{-4}, 10^{-5}, 10^{-6}, 10^{-7}, 10^{-8} and 0). Four to seven biological replicates are shown and these were used to calculate the effect sizes shown in Extended Data Fig. 7b. For IAI55, two out of six replicates were excluded because the population did not lyse at any of the tested MOIs and effect sizes could therefore not be estimated. **(b)** The same as in panel a, but showing the results of plasmid-based assays investigating phage fitness against Retron-Eco9 in BW25113 (effect sizes shown in Extended Data Fig. 7c) or **(c)** against DarTG1 in BW25113 (effect sizes shown in Extended Data Fig. 7d). Data was excluded when curves showed an inconsistent up-and-down trend (one replicate of G76F for Retron-Eco9 and one replicate of Δ*0.7* for DarTG1).
