# Supplementary material for: Pervasive phosphorylation by phage T7 kinase disarms bacterial defences
Source: Nature. 2026 Aug 19;658(8135):454–64. doi: 10.1038/s41586-026-10934-5 (PMC13645617; doi:10.1038/s41586-026-10934-5)
Supplement: Supplementary file 2 [file 41586_2026_10934_MOESM2_ESM.pdf]

nature portfolio

Corresponding author(s): Nassos Typas and Mikhail Savitski

Last updated by author(s): Jul 13, 2026

Reporting Summary

Nature Portfolio wishes to improve the reproducibility of the work that we publish. This form provides structure for consistency and transparency in reporting. For further information on Nature Portfolio policies, see our Editorial Policies and the Editorial Policy Checklist.

Statistics

For all statistical analyses, confirm that the following items are present in the figure legend, table legend, main text, or Methods section.

n/a	Confirmed	
☐	☒	The exact sample size (*n*) for each experimental group/condition, given as a discrete number and unit of measurement
☐	☒	A statement on whether measurements were taken from distinct samples or whether the same sample was measured repeatedly
☐	☒	The statistical test(s) used AND whether they are one- or two-sided<br>*Only common tests should be described solely by name; describe more complex techniques in the Methods section.*
☐	☒	A description of all covariates tested
☐	☒	A description of any assumptions or corrections, such as tests of normality and adjustment for multiple comparisons
☐	☒	A full description of the statistical parameters including central tendency (e.g. means) or other basic estimates (e.g. regression coefficient) AND variation (e.g. standard deviation) or associated estimates of uncertainty (e.g. confidence intervals)
☐	☒	For null hypothesis testing, the test statistic (e.g. *F*, *t*, *r*) with confidence intervals, effect sizes, degrees of freedom and *P* value noted<br>*Give P values as exact values whenever suitable.*
☒	☐	For Bayesian analysis, information on the choice of priors and Markov chain Monte Carlo settings
☒	☐	For hierarchical and complex designs, identification of the appropriate level for tests and full reporting of outcomes
☒	☐	Estimates of effect sizes (e.g. Cohen's *d*, Pearson's *r*), indicating how they were calculated

Our web collection on statistics for biologists contains articles on many of the points above.

Software and code

Policy information about availability of computer code

Data collection	No custom code was used for data collection. Mass spectrometry data were acquired using Thermo Fisher instrument control software (Fusion Lumos, Exploris 480, Q-Exactive Plus, and Orbitrap Astral). Bacterial growth/infection assays were recorded on BioTek Synergy HT/H1, Filtermax F5, and Molecular Devices plate readers using their respective acquisition software.
Data analysis	Data-dependent MS raw files were processed with MSConvert (v3.0.24260-9bf2070 in ProteoWizard), searched with MSFragger v4.0 in FragPipe v21 with Percolator v3.6.4, Philosopher 5.1.0 and PTMProphet (TPP v6.3.2), and data-independent MS data with DIA-NN v2.1.0. Statistical analysis used R v4.3.3 with limma v3.58.1, STRINGdb v2.14.3, and ggseqlogo v0.2, and plate-reader data were analysed in MATLAB R2017b.<br>Homology and phylogenetics used pyhmmer v0.10.15, MAFFT v7.505, FastTree v2.2, GTDB-Tk v2.1.1, and InterProScan, BLAST, and PHASTEST via their respective webservers.<br>Structural analysis used AlphaFold3, PyMOL v3.03, ChimeraX v1.10, FoldSeek, and GraphBind. Genome assembly and defense-system annotation used Flye v2.9-b1768, Prodigal v2.6.3, PADLOC v2.0.0, and DefenseFinder v1.2.2.<br>Custom code to reproduce analyses is available at Zenodo (https://doi.org/10.5281/zenodo.20727692).

For manuscripts utilizing custom algorithms or software that are central to the research but not yet described in published literature, software must be made available to editors and reviewers. We strongly encourage code deposition in a community repository (e.g. GitHub). See the Nature Portfolio guidelines for submitting code & software for further information.

Data

Policy information about [availability of data](#)

All manuscripts must include a [data availability statement](#). This statement should provide the following information, where applicable:

- Accession codes, unique identifiers, or web links for publicly available datasets
- A description of any restrictions on data availability
- For clinical datasets or third party data, please ensure that the statement adheres to our [policy](#)

All raw mass spectrometry files, search parameters, and search outputs were deposited to the ProteomeXchange Consortium via the PRIDE partner repository with the dataset identifier PXD058408. Previously published phosphoproteomics datasets reanalysed here are available from PRIDE under PXD008369, PXD008921 and PXD008289. Human phosphoproteome comparison datasets are available from their original publications.
This study also made use of the following public databases: PhosphoSitePlus (https://www.phosphosite.org), dbPSP 2.0 (http://dbpsp.biocuckoo.cn/), PHROG (https://phrogs.lmge.uca.fr/), BFVD (https://bfvd.steineggerlab.workers.dev/), proGenomes3 (https://progenomes.embl.de/), the Protein Data Bank (structures 1FIN and 1KV2; https://www.rcsb.org), KinBase (http://kinase.com/web/current/kinbase/), QuickGO (https://www.ebi.ac.uk/QuickGO/), STRING v12.0 (https://string-db.org), the Genome Taxonomy Database (https://gtdb.ecogenomic.org), PADLOC-DB v2.0.0 (https://github.com/padlocbio/padloc-db), DefenseFinder models v1.2.3 and CasFinder models v3.1.0 (https://github.com/mdmparis/defense-finder-models).
The assembled long-read sequencing genomes have been uploaded to the ECOREF website https://evocellnet.github.io/ecoref/download/, and are also available at https://figshare.com/articles/dataset/E_coli_genomes/28007045.
AlphaFold3 structural predictions of the phage T7 proteome, T7K kinase domains and selected homologues are downloadable at https://doi.org/10.5281/zenodo.14497210.
Source data for all infection assays are provided alongside custom code via Zenodo (https://doi.org/10.5281/zenodo.20727692), and raw gel images for Extended Data Fig. 4b and c in Supplementary Fig. 1.

Research involving human participants, their data, or biological material

Policy information about studies with [human participants or human data](#). See also policy information about [sex, gender (identity/presentation), and sexual orientation](#) and [race, ethnicity and racism](#).

Reporting on sex and gender	*Use the terms sex (biological attribute) and gender (shaped by social and cultural circumstances) carefully in order to avoid confusing both terms. Indicate if findings apply to only one sex or gender; describe whether sex and gender were considered in study design; whether sex and/or gender was determined based on self-reporting or assigned and methods used. Provide in the source data disaggregated sex and gender data, where this information has been collected, and if consent has been obtained for sharing of individual-level data; provide overall numbers in this Reporting Summary. Please state if this information has not been collected. Report sex- and gender-based analyses where performed, justify reasons for lack of sex- and gender-based analysis.*
Reporting on race, ethnicity, or other socially relevant groupings	*Please specify the socially constructed or socially relevant categorization variable(s) used in your manuscript and explain why they were used. Please note that such variables should not be used as proxies for other socially constructed/relevant variables (for example, race or ethnicity should not be used as a proxy for socioeconomic status). Provide clear definitions of the relevant terms used, how they were provided (by the participants/respondents, the researchers, or third parties), and the method(s) used to classify people into the different categories (e.g. self-report, census or administrative data, social media data, etc.) Please provide details about how you controlled for confounding variables in your analyses.*
Population characteristics	*Describe the covariate-relevant population characteristics of the human research participants (e.g. age, genotypic information, past and current diagnosis and treatment categories). If you filled out the behavioural & social sciences study design questions and have nothing to add here, write "See above."*
Recruitment	*Describe how participants were recruited. Outline any potential self-selection bias or other biases that may be present and how these are likely to impact results.*
Ethics oversight	*Identify the organization(s) that approved the study protocol.*

Note that full information on the approval of the study protocol must also be provided in the manuscript.

Field-specific reporting

Please select the one below that is the best fit for your research. If you are not sure, read the appropriate sections before making your selection.

☒ Life sciences ☐ Behavioural & social sciences ☐ Ecological, evolutionary & environmental sciences

For a reference copy of the document with all sections, see [nature.com/documents/nr-reporting-summary-flat.pdf](#)

Life sciences study design

All studies must disclose on these points even when the disclosure is negative.

Sample size	No prior assumptions were made regarding sample size and no power calculation was performed. Sample sizes follow standard practice in the field; experiments were performed with at least two biological replicates, and all experiments with statistical analyses were performed on at

least three independent biological replicates (e.g. independent overnight cultures), with replicate numbers for each experiment given in the figure legends and Methods section. This was sufficient as the reported effects were large and consistent across replicates. For natural-isolate experiments with expanded T7 mutants, where effect sizes were more variable, replication was increased accordingly (see Methods).

Data exclusions	Some exclusions of E. coli natural isolate strains were performed for the T7K screen (for details please see Methods).
Replication	Experiments were always conducted in at least two biological replicates (independent overnight cultures). For number of replicates for each experiment, please see figure legends and Methods.
Randomization	Randomization was not relevant to our study. Experiments compared defined, investigator-imposed conditions (e.g. infected vs. uninfected) using isogenic cultures or defined natural isolates grown under identical conditions, so there were no heterogeneous subjects to allocate across groups. Controls were processed in parallel.
Blinding	For in vitro experiments, no blinding with performed, since it was necessary to keep track of which samples are used in each experiment. Controls were always processed in parallel.

Reporting for specific materials, systems and methods

We require information from authors about some types of materials, experimental systems and methods used in many studies. Here, indicate whether each material, system or method listed is relevant to your study. If you are not sure if a list item applies to your research, read the appropriate section before selecting a response.

Materials & experimental systems

n/a	Involved in the study	
☐	☒	Antibodies
☒	☐	Eukaryotic cell lines
☒	☐	Palaeontology and archaeology
☒	☐	Animals and other organisms
☒	☐	Clinical data
☒	☐	Dual use research of concern
☒	☐	Plants

Methods

n/a	Involved in the study	
☒	☐	ChIP-seq
☒	☐	Flow cytometry
☒	☐	MRI-based neuroimaging

Antibodies

Antibodies used	Anti-His (Invitrogen, MA1-21315) at 1:2,000 dilution
Validation	Mass spectrometry

Plants

Seed stocks	*Report on the source of all seed stocks or other plant material used. If applicable, state the seed stock centre and catalogue number. If plant specimens were collected from the field, describe the collection location, date and sampling procedures.*
Novel plant genotypes	*Describe the methods by which all novel plant genotypes were produced. This includes those generated by transgenic approaches, gene editing, chemical/radiation-based mutagenesis and hybridization. For transgenic lines, describe the transformation method, the number of independent lines analyzed and the generation upon which experiments were performed. For gene-edited lines, describe the editor used, the endogenous sequence targeted for editing, the targeting guide RNA sequence (if applicable) and how the editor was applied.*
Authentication	*Describe any authentication procedures for each seed stock used or novel genotype generated. Describe any experiments used to assess the effect of a mutation and, where applicable, how potential secondary effects (e.g. second site T-DNA insertions, mosiacism, off-target gene editing) were examined.*
