# Supplementary material for: Pervasive phosphorylation by phage T7 kinase disarms bacterial defences
Source: Nature. 2026 Aug 19;658(8135):454–64. doi: 10.1038/s41586-026-10934-5 (PMC13645617; doi:10.1038/s41586-026-10934-5)
Supplement: Supplementary file 3 [file 41586_2026_10934_MOESM4_ESM.pdf]

nature portfolio

Peer Review File

Pervasive phosphorylation by phage T7 kinase disarms bacterial defenses

Corresponding Author: Dr Athanasios Typas

Any redactions in this file are there to maintain patient confidentiality, the confidentiality of unpublished data, or to remove third-party material.

This file contains all reviewer reports in order by version, followed by all author rebuttals in order by version.

Version 0:

Reviewer comments:

Referee #1

(Remarks to the Author)
The article by Bartolec et al. describes a promiscuous phage kinase that counteracts several phage defense systems encoded in the host bacteria. The authors showed that the T7 protein kinase (T7K) phosphorylates almost all host proteins upon infection. Among the most phosphorylated proteins are RNA-binding and DNA-binding proteins, which appears to be due to the C-terminal domain of T7K that is positively charged. The affinity towards DNA-binding proteins helps T7K inhibit several phage defense systems, thereby helping T7 infect a broader range of cells.

This work is a tour-de-force that demonstrates a surprisingly general kinase ever found. Although the authors chose to focus on the effects of phosphorylation on phage defense systems, they also found that T7K modifies many other host proteins with high efficiency, which may have many important biological consequences. This study therefore opens new avenues for investigating how phage shuts off or manipulate the host machinery to its own benefits.

Shortly before this work was submitted, another article was published by Jiang et al. in Nature Microbiology that describes a homologous phage-encoded kinase as anti-defense systems (doi.org/10.1038/s41564-024-01851-2). These two studies have largely consistent results. The main advances in the current study by Bartolec et al. involve proteome-wide characterization of phosphorylation sites and identification of the C-terminal shut-off domain.

Main comments:
1. Many of the heavily phosphorylated proteins are related to RNA metabolism (RNase E, translation factors, etc.), which cannot be explained by its affinity to DNA. Considering that there is much more RNA than DNA in the cell, how is T7K not sequestered by the vast amount of RNA and ribonucleoproteins? Without direct evidence that T7K binds to DNA in vivo, it is possible that the ability to modify DNA-binding protein is in fact mediated via RNA and the proximity of RNA to DNA due to transcription-translation coupling. It will be important to rule out this model.
2. To establish that nucleic acid binding proteins are enriched in T7K targets, the authors applied GO-term enrichment analysis to highly modified substrates. It is unclear to me whether an appropriate background set of proteins was used in this analysis. As the authors pointed out, the ability to detect stoichiometrically phosphorylated proteins is influenced by peptide abundance. According to the Methods section, the background set of proteins was selected with essentially no cutoff in abundance. Could it be that the RNA-binding proteins appear to be enriched simply due to their abundance?
3. I do not understand the "normalized AUC" metric in Fig 3 and 4. Please explain what it means biologically.
Minor comments:
4. In line 254, the sentence "We found three systems (retrons Eco6 and Eco9, and DarTG1) defending against T7 phage infection" appears to be contradict with Fig. 3b. Fig. 3b shows that these systems fail to defend wildtype T7, but are effective when T7K is knocked out.

Referee #2

(Remarks to the Author)
Bartolec and colleagues, investigate in this manuscript a phage-encoded kinase (T7K) from bacteriophage T7 and demonstrates that it is a hyper-promiscuous, dual-specificity enzyme capable of depositing an unprecedented number of phosphorylation sites on both host and phage proteins during infection. This study addresses a long-standing puzzle in phage biology: how phages counteract the rapidly evolving battery of bacterial defense systems. Phage–host arms races are central to microbial ecology, and understanding the molecular mechanisms by which phages disable multiple different bacterial defenses is interesting. The authors employ state-of-the-art, time-resolved quantitative phosphoproteomics to reveal that T7K preferentially targets nucleic acid–binding proteins via its C-terminal domain, which directly binds DNA/RNA. Functionally, the study shows that phosphorylation of key residues in defense proteins such as Retron-Eco9 and DarTG effectively inactivates these systems. By screening a diverse library of natural E. coli isolates, the work extends its impact, suggesting that this counter-defense mechanism is broadly relevant in nature. Overall, the research addresses an important question in microbial defense, linking biochemical mechanism with evolutionary implications.

Major Concerns

1 - Overall, the work is technically very well done and very complete as it is. The authors convincingly show that T7K is hyper-promiscuous and uses its C-terminal nucleic acid–binding domain to target defense proteins. However, it remains unclear how generalizable these findings are to other related phage kinases.

1.1 - How common is this mode of action among other phage-encoded kinases? Are there structural or sequence similarities among kinases from diverse phages that suggest a conserved mechanism? Are phage kinases found with an array of other protein domains that might suggest what they could be targetting, like in the current example of DNA/RNA binding region ? A broader phylogenetic analysis and comparison with kinases found in prophage regions could be valuable. The authors could additionally perform the phosphoproteomics studies in the presence of other phage encoded kinases to determine the degree of generalization for the proposed mechanism. The authors mention a related study with a Salmonella phage JSS1 but is unclear if that study was done with the same degree of sensitivity in the MS profiling.

1.2 - The manuscript would benefit from discussing the evolutionary pressures that might have driven the divergence of substrate specificity, possibly providing insights into how phage kinases evolved toward promiscuity. Having some phosphoproteomic data on divergent phage kinases would make such an analysis even stronger.

2 - The manuscript acknowledges overlap with a recent paper (Jiang et al. Nature Microbiology 2024). It seems to me that the current study proposes a more general mechanism of a promiscuous kinase that can target a large number of different defence systems. Besides this, the level of technical detail and analysis of diverse lab strains done here seem much more in depth than Jiang et al. Again here, the current study would be more differentiated if the authors could study the generality of their findings across other phage kinases through computational and experimental approaches.

Minor Concerns

- line 107 – “Notably, no close homologues of T7K were found outside of phages” – what does “close” here mean. As described above, it would have been nice to have a better sense of the diversity of phage kinases and how they relate to other ST or STY kinases.

- The large degree of phosphorylation of the host proteins is very interesting and it is surprising that it does not generally lead to a massive disruption of protein function. Are the host protein phosphorylation sites mostly occurring in regions that are predicted to be accessible ?

- line 147 to 149 – The authors suggest that early phage proteins are perhaps selected to avoid having phosphorylable residues. This is an interesting idea but not really pursued in the manuscript. This is not something that I feel is very important but the authors could have tried to show that phages carrying related kinases have such sequence selection pressures to avoid phosphorylation of page proteins.

- when performing the analysis of stoichiometry, I wonder if there could be some bias to quantify stoichiometry dependent on the abundance of the protein. I don’t think this should be the case but the authors could check.

Referee #3

(Remarks to the Author)
This study investigates the T7 phage kinase and its role in disarming anti-phage defense systems. The authors perform a high quality phospho-proteome analysis, showing that T7K phosphorylates S/T/Y residues throughout the proteome. The analysis of peptides phosphorylated at a high stoichiometry shows a preference for nucleic acid binding proteins that is likely mediated by the SO domain of the kinase.
The authors then convincingly show that the T7K enables T7 to bypass the Retron-Eco9 and DarTG defense systems. They identify phosphorylation sites in proteins of these systems and show that phosphomimic mutants are indeed unable to provide defense. Finally, the authors screen the WT T7 and the T7K deletion mutant against a large collection of E. coli strains. This enabled the identification of a few strains that cannot be efficiently infected in the absence of T7K and one

strain that the T7K mutant infects better than the WT. These results suggest that the T7K can inhibit a wide range of defense systems, and that strain IAI15 might contain a defense system triggered by the T7K. The identity of this system is not revealed here.

Altogether these results are consistent with a recent report investigating JSS1_004, an homologue of T7K, and which also described broad S/T/Y phosphorylation. The study by Bartolec et al. is quite complementary in several respects. The phosphoproteome analysis performed here is much deeper, revealing a more detailed picture of the T7K activity. The set of defense systems found to be inhibited is different, reinforcing the picture of a broad anti-defense mechanism. This study also opens multiple research avenues by uncovering multiple strains affected one way or another by the T7K. The description of this family of kinases is a significant finding in the field of bacterial immunity, and this study an important contribution. The impact of the present work would however be made higher by further improving the aspects that differentiate it from the published work on JSS1.

Main comments:
Figure 2D shows an increased odds ratio for nucleic acid binding proteins but not for DNA binding. Could you clarify why that is the case? How is this consistent with your hypothesis that DNA binding directs preferential phosphorylation? Figure 2b could also use a representation (maybe color code) to highlight which of the proteins are known to interact with DNA or RNA.

It would be interesting to test solubilization of the SO domain not just with DNA but also with RNA. Having some biochemical data to compare DNA vs. RNA binding affinity of the SO domain could also be helpful.

At the moment the role of the SO domain in mediating preferential phosphorylation is not directly shown by the authors. A direct demonstration of this would require performing the phospho-proteome analysis in a T7K mutant lacking the SO domain. Such a mutant would also be interesting to test against the anti-phage defense systems.

T7 relies on nucleic acid-binding proteins for its cycle, including some of its own proteins such as the T7 RNAP. Are any of the T7 proteins phosphorylated at high stoichiometry? How does the solvent accessibility of, S, T and Y in phage proteins compare to that of E. coli proteins ? Do the proteins phosphorylated at high stoichiometry show better solvent accessibility?

The authors discuss RcsB in the context of defense against phage infection mediated by the colonic acid capsule, but I struggle to understand how T7 would benefit from inactivating RcsB when it is already inside the cell? Could the authors clarify their thinking?

Version 1:

Reviewer comments:

Referee #1

(Remarks to the Author)
The authors have address all my comments. I congratulate them for a very interesting work.

Referee #2

(Remarks to the Author)
The authors have addressed all of the concerns raised very extensively. The additional phosphoproteomic analysis of other related phage kinases, the phylogenetic and bioinformatic structural studies really add to the generalization of their findings beyond the initially studied kinase. This is a really complete and interesting study. I have no further concerns.

Referee #3

(Remarks to the Author)
The authors have addressed all my concerns and I support the publication of this article.

Open Access This Peer Review File is licensed under a Creative Commons Attribution 4.0 International License, which permits use, sharing, adaptation, distribution and reproduction in any medium or format, as long as you give appropriate credit to the original author(s) and the source, provide a link to the Creative Commons license, and indicate if changes were made.
In cases where reviewers are anonymous, credit should be given to 'Anonymous Referee' and the source.
The images or other third party material in this Peer Review File are included in the article's Creative Commons license, unless indicated otherwise in a credit line to the material. If material is not included in the article's Creative Commons license and your intended use is not permitted by statutory regulation or exceeds the permitted use, you will need to obtain permission directly from the copyright holder.
To view a copy of this license, visit https://creativecommons.org/licenses/by/4.0/

Rebuttal

We thank the reviewers and the editor for their time and effort spent considering our manuscript. Your comments helped us to significantly improve the manuscript. In this revised version, we have made several changes to incorporate new experimental and computational data, which address all questions raised. Three of the four main figures have been expanded to feature new data, and an additional fifth figure has been added to report on new findings about the conservation of T7K and evolutionary considerations on its function and promiscuity. Overall, this has led to expanding the utility and generalizability of our study.

Specifically, we:

- validate that the SO domain directs preference of the kinase to nucleic-acid binding substrates through its DNA-binding properties, and also obtain evidence that it is important for maintaining the kinase active for longer periods, likely through slowing down its catalytic autoinhibition
- show that the shutoff domain does not bind to RNA
- identify T7K homologues and characterize their phylogeny, sequence features, domain architecture, prevalence and the lifestyle of the encoding phages
- demonstrate the generalizability of kinase hyperpromiscuity for T7K homologues by mapping the phosphoproteomes during infection by several T7-related phages in the BASEL collection
- show that phage-mediated kinase signaling is not always promiscuous, by studying other phages within the BASEL collection known/predicted to have kinases
- discover a new kinase motif (DPV) that is specific to T7K and its homologues, which is related to the canonical kinase DFG active site motif (DFG-in, DFG-out) and may provide clues towards the mechanisms of T7K promiscuity

Below we contextualize these new experiments into a point-by-point response to each reviewer's comments, and also explain several analyses that we attempted/performed but will not report in the revised manuscript.

Referee expertise:

Referee #1: bacterial proteomics
Referee #2: phosphorylation, kinase signalling and evolution
Referee #3: phage-bacteria interactions, anti-phage defences

Referees' comments:

Referee #1 (Remarks to the Author):

The article by Bartolec et al. describes a promiscuous phage kinase that counteracts several phage defense systems encoded in the host bacteria. The authors showed that the T7 protein kinase (T7K) phosphorylates almost all host proteins upon infection. Among the most phosphorylated proteins are RNA-binding and DNA-binding proteins, which appears to be due to the C-terminal domain of T7K that is positively charged. The affinity towards DNA-binding proteins helps T7K inhibit several phage defense systems, thereby helping T7 infect a broader range of cells.

This work is a tour-de-force that demonstrates a surprisingly general kinase ever found. Although the authors chose to focus on the effects of phosphorylation on phage defense systems, they also found that T7K modifies many other host proteins with high efficiency, which may have many important biological consequences. This study therefore opens new avenues for investigating how phage shuts off or manipulate the host machinery to its own benefits.

Shortly before this work was submitted, another article was published by Jiang et al. in Nature Microbiology that describes a homologous phage-encoded kinase as anti-defense systems (doi.org/10.1038/s41564-024-01851-2). These two studies have largely consistent results. The main advances in the current study by Bartolec et al. involve proteome-wide characterization of phosphorylation sites and identification of the C-terminal shut-off domain.

We thank the reviewer for their generous and encouraging assessment of our work. We are pleased that the reviewer recognizes the breadth of T7K activity and the significance of its role in counteracting diverse bacterial defense systems. We also appreciate their highlighting of the additional implications of widespread host phosphorylation, which indeed opens exciting avenues for further investigation into how phages manipulate host cellular processes.

Main comments:

1. Many of the heavily phosphorylated proteins are related to RNA metabolism (RNase E, translation factors, etc.), which cannot be explained by its affinity to DNA. Considering that there is much more RNA than DNA in the cell, how is T7K not sequestered by the vast amount of RNA and ribonucleoproteins? Without direct evidence that T7K binds to DNA in vivo, it is possible that the ability to modify DNA-binding protein is in fact mediated via RNA and the proximity of RNA to DNA due to transcription-translation coupling. It will be important to rule out this model.

Thank you for this comment, we have now addressed it with the following experiment.

In the original refolding assay **(Fig. 2f)**, we demonstrated that DNA is able to directly interact with the purified T7K shutoff (SO) domain in the absence of RNA. To test whether the SO domain is also able to bind to RNA molecules, as would be suggested by the GO-term enrichment in our stoichiometry analysis and by the GraphBind prediction **(Fig. 2e)**, we extended the refolding assay in the presence of total *E. coli* RNA **(revised Fig. 2f with additional condition and updated schematic)**. For these samples, we also included RNase inhibitors and performed parallel control experiments to confirm RNA integrity in the conditions of the refolding assay (high urea, dialysis, potential residual RNases from the protein purification) and in the different fractions **(Extended Data Fig. 4c).** This revealed that RNA could not assist refolding in the same manner as DNA. Therefore, we currently have no experimental evidence to support direct RNA-binding of the T7K SO domain, which is something we state also in the manuscript. High stoichiometry RNA-binding substrates could be indeed related to transcription-translation coupling, to their high abundance in the cell, but also to T7K binding concentrating towards tRNA/rRNA DNA loci (see below).

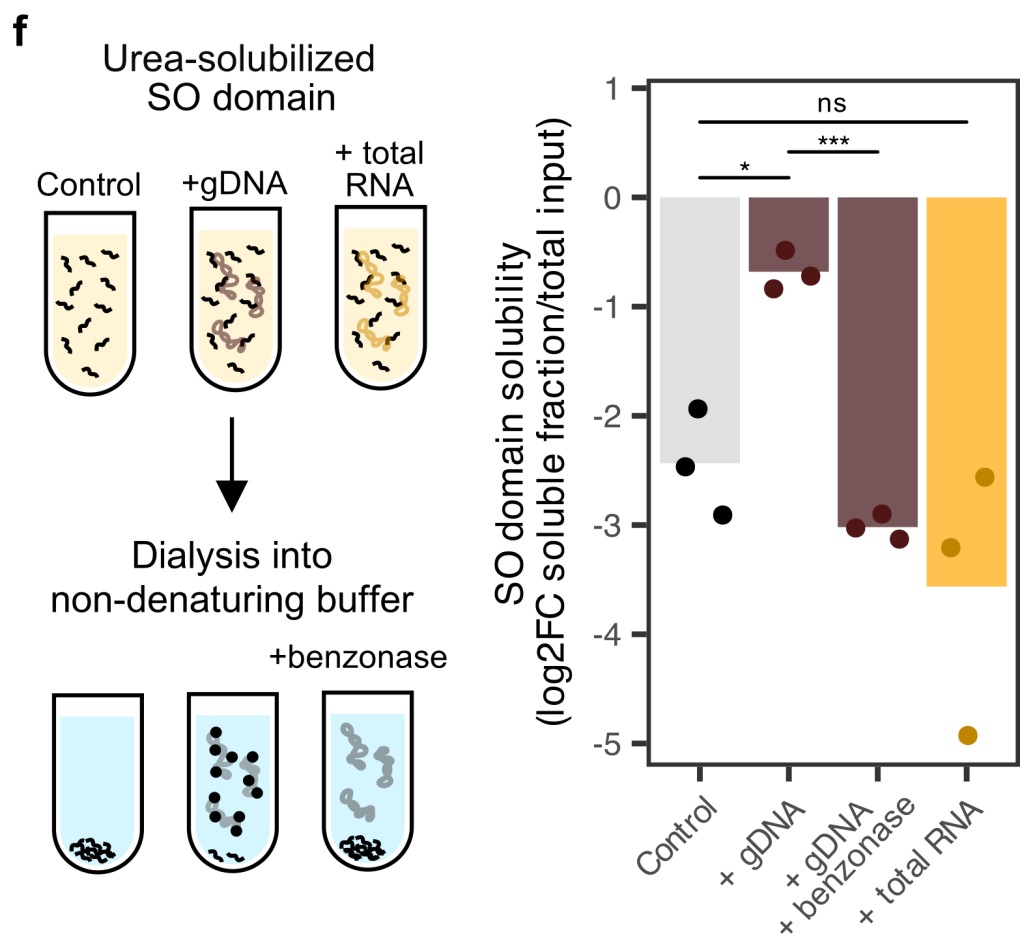

*__Revised Figure 2f:__ The T7K shutoff (SO) domain binds DNA but not RNA. Recombinant 6xHis-TEV-SO (SO) is insoluble (Extended Data Fig. 4b). Urea-solubilized recombinant SO folded into a soluble protein in non-denaturing buffer in the presence, but not in the absence of genomic DNA (gDNA) or in the presence of total RNA. Benzonase treatment reverted solubility, indicating that the SO domain assumes stable confirmation only when bound to DNA. Relative SO-domain solubility (abundance in the soluble fraction compared to the total input) was measured using TMT-multiplexed MS and shown as bar plot (N = 3 independent biological replicates; unpaired t-test analysis – ns (non-significant), * – p-value≤0.05, *** – p-value≤0.001).*

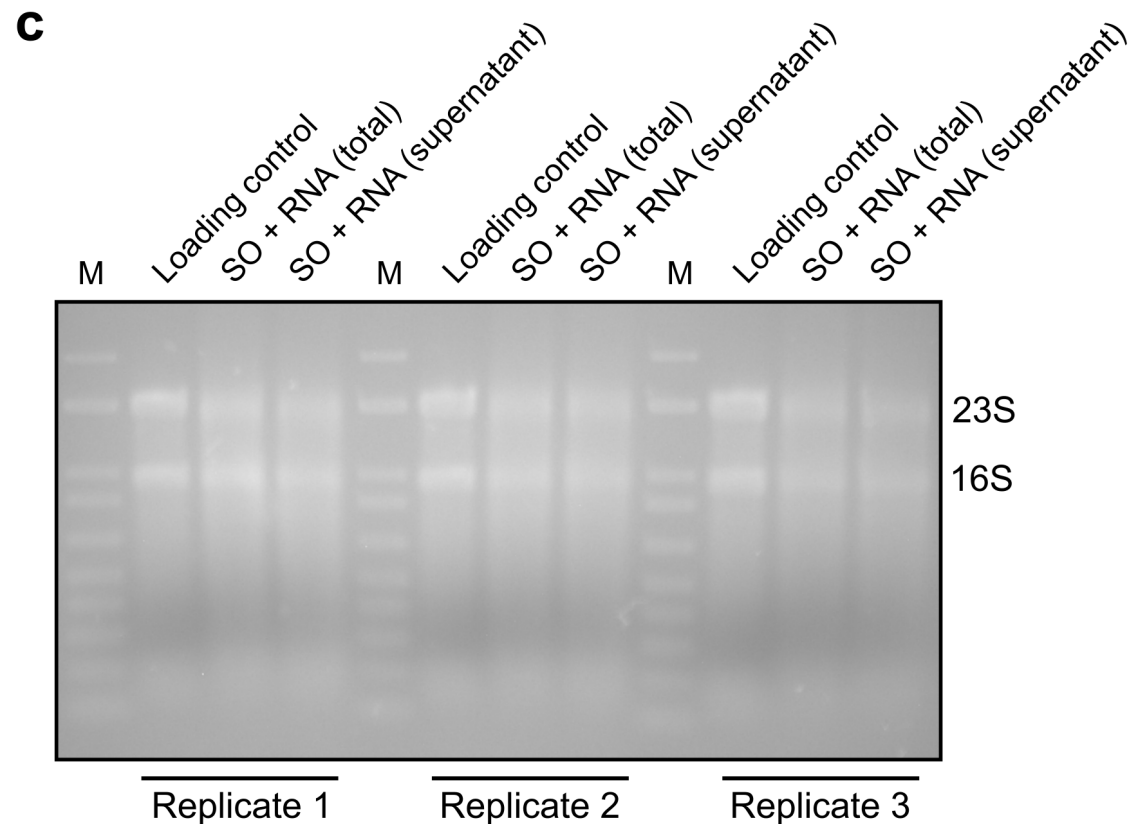

Extended Data Figure 4c: *Assessment of RNA integrity after overnight dialysis during in vitro refolding of 6xHis-TEV-SO. Samples from refolding reactions (total and supernatant) in the presence of RNA were analysed by electrophoresis on 1.5% agarose and ethidium bromide staining. Equivalent amounts of the RNA stock sample were loaded as controls. RNA integrity was assessed by comparing 23S and 16S bands from refolding reactions to controls. M, molecular weight marker.*

We note that our finding of a DNA-binding (and not RNA-binding) SO domain also corroborates our existing functional data, where defense systems found to be sensitive to T7K (Retron-Eco9, DarTG1) contained at least one protein with a link to DNA-related functions **(Fig. 3b)**. This is also in line with the findings of Jiang et. al. [1] regarding a homologous kinase in *Salmonella* phage JSS1 (JSS1_004), where all five kinase-sensitive defense systems had clear links to DNA interaction (DndFGH, CRISPR type I-E, SIR2+HerA, DUF4297+HerA, QatABCD through TatD domain). Finally, a recent study published since our manuscript submission [2] reported on ChIP-seq experiments characterizing the in vivo DNA-binding properties of JSS1_004 (enriched in rRNA/tRNA loci), providing orthogonal evidence that T7K-like kinases can bind DNA.

Action:

- We introduced a new experiment to expand upon the shutoff domain refolding assay to assess RNA-binding. We extend the existing **Fig. 2f** with this new data.

- We also introduce a new panel in the extended data describing control experiments to confirm integrity of total RNA preparations in the refolding experiment **(Extended Data Fig. 4c)**.

- We cite the new JSS1_004 ChIP-Seq findings in the following sentence:

  *"Studies on Salmonella phage JSS1, which carries a kinase with 54% amino-acid sequence identity to T7K **(Fig. 5b)**, recently demonstrated that this kinase could bind DNA and inactivate two other DNA-targeting defense systems by phosphorylating them at critical residues (Dnd, QatABCD). Involvement in deactivation for other three further systems with clear links to DNA interaction was also provided."*

- We include the following sentence in discussion to propose the suggested model relating to a link to transcription-translation coupling

  "This loose cannon kinase gains specificity towards proteins binding DNA or different RNA species through its C-terminal shutoff (SO) domain that binds DNA, and manages to stoichiometrically phosphorylate DNA and RNA-binding proteins (the latter possibly through the tight coupling between transcription and translation in prokaryotes), within its short window of activity."

2. To establish that nucleic acid binding proteins are enriched in T7K targets, the authors applied GO-term enrichment analysis to highly modified substrates. It is unclear to me whether an appropriate background set of proteins was used in this analysis. As the authors pointed out, the ability to detect stoichiometrically phosphorylated proteins is influenced by peptide abundance. According to the Methods section, the background set of proteins was selected with essentially no cutoff in abundance. Could it be that the RNA-binding proteins appear to be enriched simply due to their abundance?

Thank you for this comment, please see the detailed reply below.

Firstly, the reviewer correctly states that the GO-term enrichment analysis of high differential stoichiometry T7K-substrates involved no cut-off on protein abundance. This analysis used all identified proteins as background, as stated in the legend text for **Fig. 2b**.

Secondly, to investigate whether there were any biases in the abundance of RNA- and DNA-binding proteins that could impact our findings from the stoichiometry analysis, we compared the unique protein intensities of host proteins from the TMT proteomics dataset and stratified them by their membership in relevant GO-term groups **(Rebuttal Fig. 1)**. RNA-binding proteins were indeed significantly higher in abundance than DNA-binding proteins, and hence would be less sensitive to technical limitations in sensitivity for the phosphatase stoichiometry experiment.

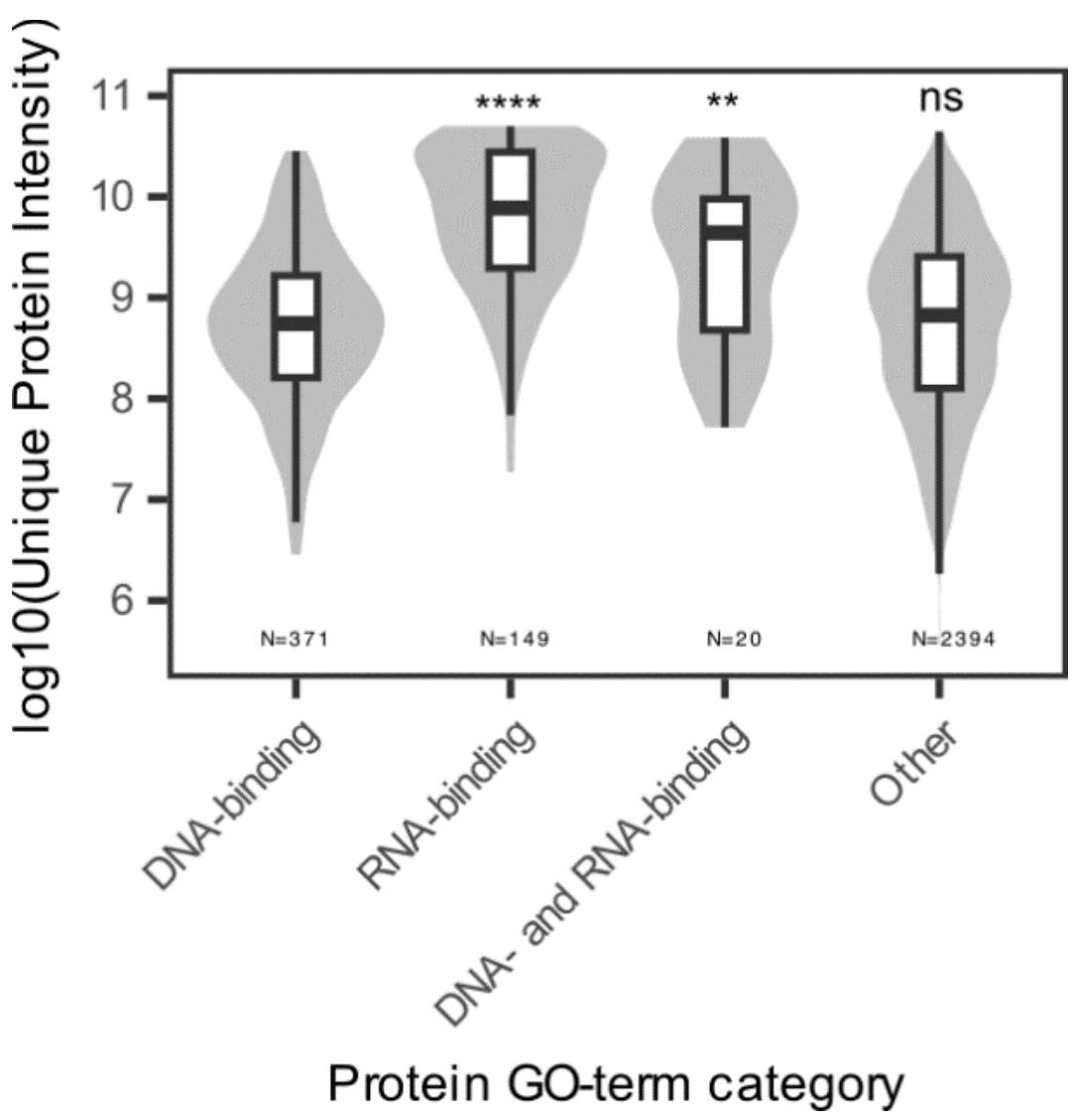

Rebuttal Figure 1:** Unique protein intensity of proteins measured in the TMT protein expression dataset stratified by whether they had "DNA binding" or "RNA binding" GO-terms, or both, or else neither "Other". Asterisks indicate statistical significance (unpaired t-test) compared to the DNA-binding group; ** – p-value≤0.01, * – p-value≤0.0001, ns – not significant, p-value≥0.05.*

Action:

- Given our existing data showing direct DNA-binding of the SO-domain which corroborates our GO-term analysis findings, we propose to keep the existing GO-term analysis as originally reported (using all detected proteins as background, no abundance cut-off).

3. I do not understand the "normalized AUC" metric in Fig 3 and 4. Please explain what it means biologically.

The "normalized AUC" metric reports on bacterial growth during infection (measured by following OD of culture over time – 6hrs) relative to an uninfected control over all tested MOIs. The plot thereby indicates at which MOIs the defense system can still defend against the phage (no lysis of the culture, normalized AUC values close to 1), and at which MOIs the defense breaks down (bacterial lysis, optical density drops, normalized AUC values close to 0). The strength of the defense can therefore be read from that representation.

In particular, for each tested MOI, the "normalized AUC" is calculated as the area under the optical density curve over time (linear scale, until 6 hours of infection) normalized by the area under the optical density curve over time of the uninfected control (linear scale, until 6 hours of infection). The normalized AUC for a chosen defense system has values between 0 and 1 at a specific MOI when the defense system shows some defense at the beginning of the growth assay, but fails to defend at later times (within the 6 hours of infection) and the bacterial population collapses. The 6 hours of infection were chosen, as the bacterial population stayed stable after 6 hours (either it had collapsed already earlier or the bacterial cells stayed alive).

Action:

- We have rephrased and added the following sentences to the legend text of **Fig. 3b** to help the readers better understand the normalized AUC metric (and more information is given in methods):

  > *"(b) The defense systems Retron-Eco9 and DarTG1 defend against T7 and are sensitive to the T7K. For each system, a representative growth curve at a selected MOI distinguishing WT from the 0.7 knockout mutant, and area under the curve normalized by the uninfected control (norm. AUC) over all tested MOIs, are shown. The norm. AUC metric summarizes growth curves across all MOIs and reflects phage efficiency and bacterial defense strength: more effective phages (bypassing defense systems) show low norm. AUC at lower MOIs*".

Minor comments:

4. In line 254, the sentence "We found three systems (retrons Eco6 and Eco9, and DarTG1) defending against T7 phage infection" appears to be contradict with Fig. 3b. Fig. 3b shows that these systems fail to defend wildtype T7, but are effective when T7K is knocked out.

We thank the reviewer for the question and want to clarify this point. Indeed, the upper panels in Figure 3b show that Retron-Eco9 and DarTG1 fail to defend against T7 WT whereas they defend against the T7K knockout at the indicated MOIs. In the lower plots ("norm. AUC") in Figure 3b, however, one can appreciate that this failure to defend against T7 WT is only true for the higher MOIs tested. For lower MOIs (10^{-5} for both Retron-Eco9 and DarTG1, and also 10^{-4} for Retron-Eco9), the systems also defend against T7 WT (norm. AUC is close to 1 and therefore bacterial cells are growing like an uninfected control at these MOIs). This defense improves in the T7K knockout (full defense also present at higher MOIs), supporting that T7K weakens the defense of Retron-Eco9 and DarTG1.

Action:

- The rephrasing and the additional sentence added to **Fig. 3b**, as stated above, helps the readers to understand the norm. AUC metric better.

- We added a half-sentence which makes it clearer that defense is dependent on the MOI:

  "We found three systems (retrons Eco6 and Eco9, and DarTG1) defending against T7 phage infection at least at the lowest MOI tested*, the first two in line with previous reports."*

Referee #2 (Remarks to the Author):

Bartolec and colleagues, investigate in this manuscript a phage-encoded kinase (T7K) from bacteriophage T7 and demonstrates that it is a hyper-promiscuous, dual-specificity enzyme capable of depositing an unprecedented number of phosphorylation sites on both host and phage proteins during infection. This study addresses a long-standing puzzle in phage biology: how phages counteract the rapidly evolving battery of bacterial defense systems. Phage–host arms races are central to microbial ecology, and understanding the molecular mechanisms by which phages disable multiple different bacterial defenses is interesting. The authors employ state-of-the-art, time-resolved quantitative phosphoproteomics to reveal that T7K preferentially targets nucleic acid–binding proteins via its C-terminal domain, which directly binds DNA/RNA. Functionally, the study shows that phosphorylation of key residues in defense proteins such as Retron-Eco9 and DarTG effectively inactivates these systems. By screening a diverse library of natural E. coli isolates, the work extends its impact, suggesting that this counter-defense mechanism is broadly relevant in nature. Overall, the research addresses an important question in microbial defense, linking biochemical mechanism with evolutionary implications.

We thank the reviewer for this very positive feedback.

Major Concerns

1 - Overall, the work is technically very well done and very complete as it is. The authors convincingly show that T7K is hyper-promiscuous and uses its C-terminal nucleic acid–binding domain to target defense proteins. However, it remains unclear how generalizable these findings are to other related phage kinases.

1.1 - How common is this mode of action among other phage-encoded kinases? Are there structural or sequence similarities among kinases from diverse phages that suggest a conserved mechanism? Are phage kinases found with an array of other protein domains that might suggest what they could be targetting, like in the current example of DNA/RNA binding region ? A broader phylogenetic analysis and comparison with kinases found in prophage regions could be valuable. The authors could additionally perform the phosphoproteomics studies in the presence of other phage encoded kinases to determine the degree of generalization for the proposed mechanism. The authors mention a related study with a Salmonella phage JSS1 but is unclear if that study was done with the same degree of sensitivity in the MS profiling.

We thank the reviewer for their complimentary view of our study and below provide our explanation of new analyses and findings related to several questions within comment #1.1, in a sentence-by-sentence manner.

How common is this mode of action among other phage-encoded kinases?

To investigate if our findings of T7K hyperactivity can generalize to other viral kinases, we introduce new experimental datasets investigating the phosphoproteome induced by infection of *E. coli* K12 with several other phages in the BASEL collection of *E. coli* lytic phages[3] **(new Fig. 5a, new Extended Data Fig. 8)**. Within this collection there are five other phages encoding homologous kinases (*Autographiviridae* family - Bas64, Bas65, Bas66, Bas67 and Bas68), and one phage with a different known kinase gene (P1*vir* with Doc kinase) Note: we profile and report our findings on all above *Autographiviridae* except Bas67, because of purity of stock. Our new experiments have revealed that whilst all tested *Autographiviridae* encode hyperpromiscuous and hyperactive protein kinases, not all phage kinases act in this manner, with the kinase within P1*vir* modifying much more restrained but still distinct substrate pools (**Extended Data Fig. 8c, d)**.

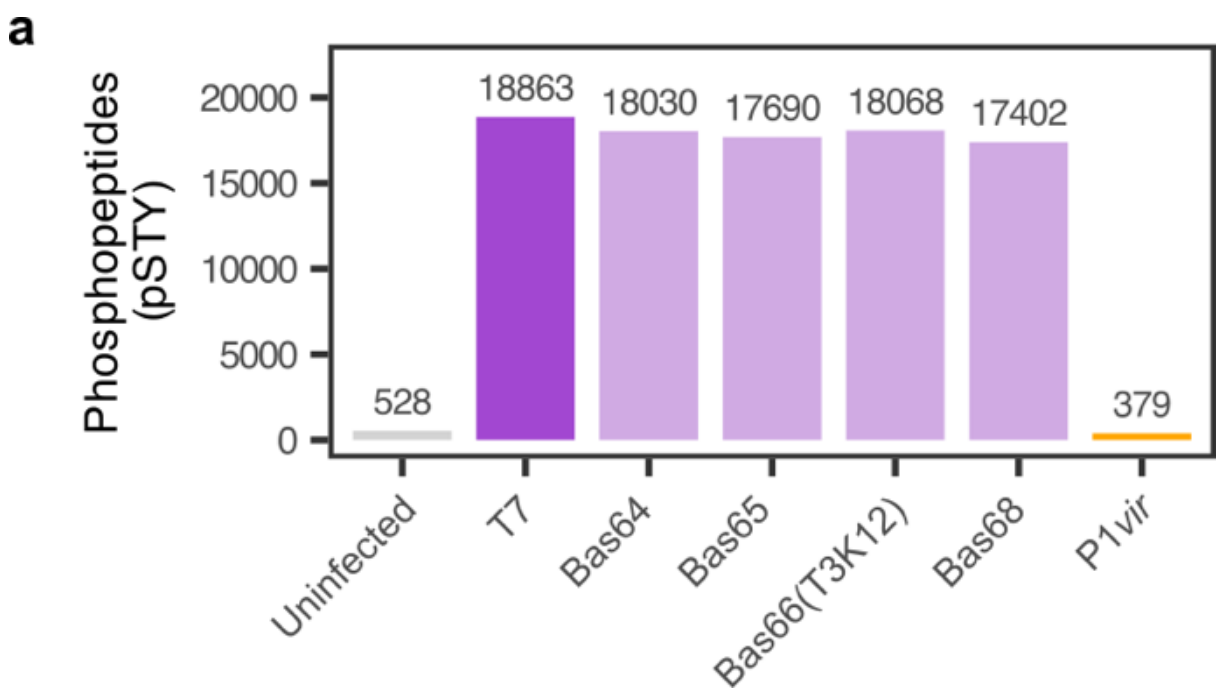

New Figure 5: Conserved hyperactivity and sequence features of T7K homologues. ***(a)*** *Hyperactivity is a conserved but unique feature of T7K homologues. Total number of unique serine (S), threonine (T) and tyrosine (Y) phosphopeptides identified from three biological replicates using phosphoenrichment and label-free MS/MS analysis of uninfected or phage-infected E. coli K12. Light purple indicates infections from Autographiviridae within the BASEL collection, which all encode T7K homologues. Orange indicates P1vir, which is known to encode an unrelated protein kinase, Doc kinase. Bas64, Bas65 and Bas66(T3K12) were sampled at 10 min, Bas68 at 5 min, and P1vir at 45 min.*

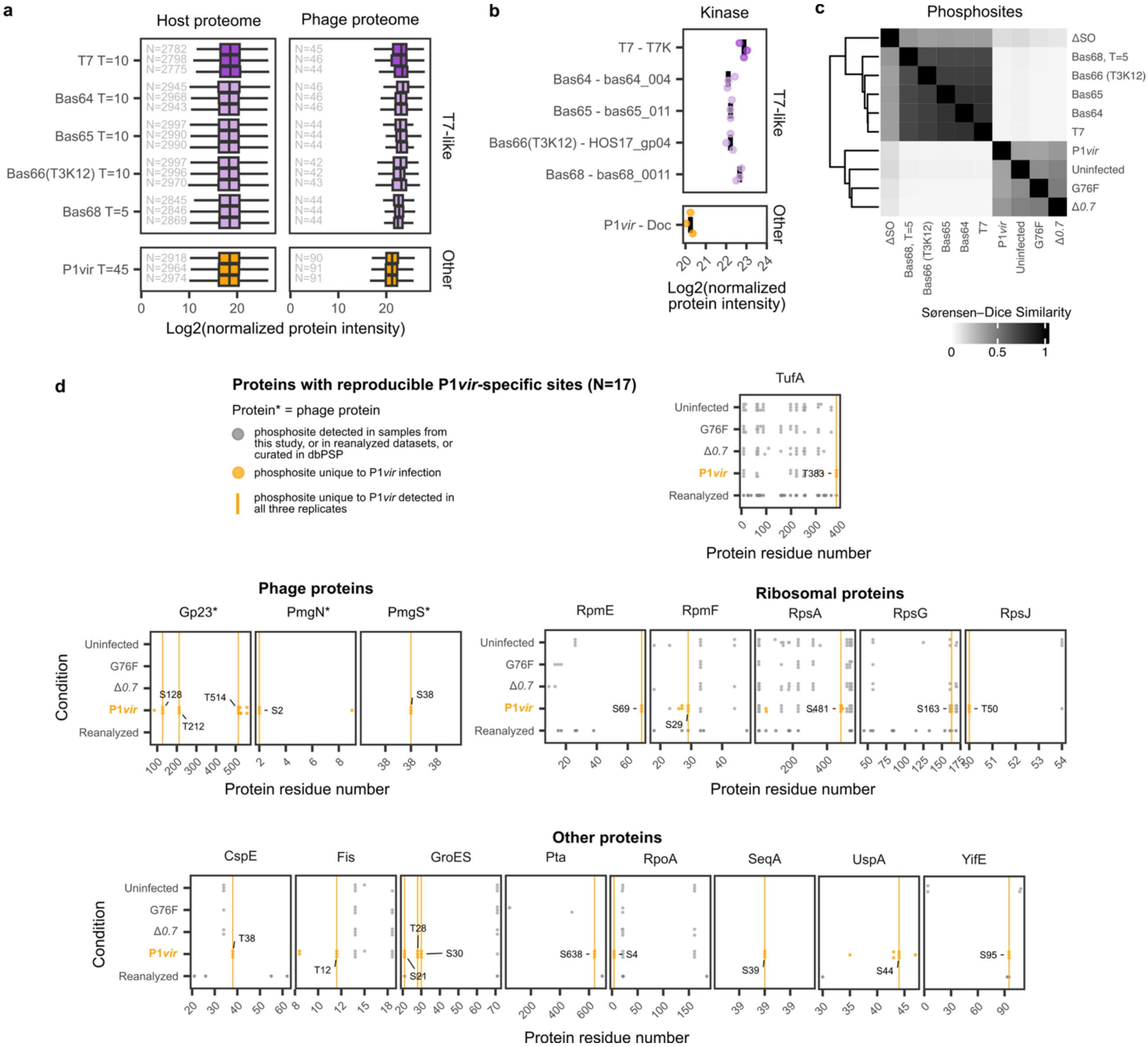

Extended Data Figure 8. Proteomics and phosphoproteomics of phages encoding diverse kinases. *Protein expression analysis (label-free data-independent analysis) was performed on samples from Fig. 5a to (a) confirm progression of phage infection cycle and (b) confirm expression of kinases at the sampled time-points. For both panels, the results from three independent biological replicates are shown. Boxplots in (a) show the distributions of all protein intensities in host or phage proteomes per replicate (showing statistics as in Extended Data Fig. 2i), with the N= indicating the number of underlying proteins (with at least one unique peptide) quantified in each replicate. In (b), the gene name and/or locus tag of the relevant kinase/s is indicated next to the virus name. (c) Similarity matrix of different infections based on identified phosphosites. Sites were derived from phosphopeptides reported in Fig. 1b (Δ0.7, G76F), Fig. 2g (ΔSO) and Fig. 5a (uninfected, T7, BASEL phages, P1vir), each from three biological replicates. Rows and columns represent individual infections. The Sørensen-Dice coefficient quantifies how similar two sets are, emphasizing the proportion of shared elements relative to their combined size, where similarity values range from 0 (no overlap; white) to 1 (identical; black). Rows are hierarchically clustered using average linkage based on their dissimilarity, i.e. 1 - Dice similarity, with the resulting dendrogram shown. (d) Proteins uniquely and reproducibly phosphorylated during P1vir infection. Each dot is an identification of a phosphopeptide at the indicated site in one of three independent biological replicates from label-free data-dependent analysis of E. coli K12 after infection with the indicated phages. The same datasets as detailed above in panel c were considered, except sites from WT and ΔSO T7 infections which were neither considered nor plotted. Note: all phosphopeptides are considered regardless of uniqueness within the FASTA database to enable visualization of the known Doc kinase site at TufA-383 (represented by a peptide sequence shared between TufA and TufB). Sites in the dbPSP database and those from the reanalyzed phosphoproteomics datasets in Extended Data Fig. 1a are also shown but are not considered when assessing uniqueness to P1vir infection. Orange dots indicate sites that only come up in P1vir, with a vertical orange line indicating the further subset of these sites which were identified in all three replicates. Proteins with an asterix are phage proteins.*

Action:

- We include new phosphoproteomics datasets investigating phages encoding diverse kinases, reported in the new **Fig. 5a** and new **Extended Data Fig. 8**.
- We report our new findings from these datasets under the new Results section subheading entitled "*Hyperpromiscuous kinase activity is conserved among T7K homologues*", which is also included verbatim below:

"*To investigate whether hyperpromiscuous kinase activity is common in phages, we searched for known protein kinases within the BASEL collection of E. coli lytic phages. We identified at least four other phages in the same family as T7 encoding T7K homologues (Autographiviridiae; Bas64, Bas65, Bas66(T3K12), Bas68), and the model phage P1vir which is known to encode the Doc kinase toxin*[4]*. We collected a single time-point during infection of E. coli K12, choosing 10 minutes for the T7-like phages (except Bas68, where we collected 5 minutes post-infection due to its faster infection dynamics), and a later time-point for P1vir (45 min) due to its longer infection cycle. Expression proteomics confirmed that each phage's infection cycle had progressed, as the majority of their proteins, including the kinase of interest, could be detected (Extended Data Fig. 8a-b). Phosphoproteomes of the same samples revealed extensive phosphorylation triggered by the Autographiviridiae phages (Bas64, Bas65, Bas66(T3K12), Bas68 - all encode T7K homologues), similar to that observed for T7K, but not by P1vir (Fig. 5a, Extended Data Fig. 8c, Supplementary Table 8). Among the tested phages, the kinase from Bas68 most closely resembles JSS1_004 from Salmonella phage JSS1 (95% sequence identity within the kinase domain) (Fig. 5b), which was reported to be unspecific albeit to a lower degree (~20-fold lower phosphorylation) than T7K. Given their high similarity, and the kinase hyperpromiscuity across all other Autographiviridiae tested here, the reported difference in activity for T7K and JSS1_004 likely arises from a more sensitive phosphoproteomics approach used in this work.*

On the other hand, phosphoproteome investigations after infection with P1vir, which encodes an unrelated kinase, revealed that not all phage kinases are hyperpromiscuous. Despite its low expression during infection (Extended Data Fig. 8b), we could reproducibly detect the single known phosphorylation site of Doc kinase (elongation factor Tu 1; TufA-T383). P1vir infection produced a phosphoproteome profile distinct from those of uninfected samples, T7 infection and of T7 mutants (Extended Data Fig. 8c) - with 20 additional sites across 17 proteins identified reproducibly and uniquely during P1vir infection (Extended Data Fig. 8d, Supplementary Table 8), including three phage proteins. These new sites may represent undescribed substrates of the Doc kinase or a host response specific to P1vir infection.

In summary, although the hyperpromiscuity and hyperactivity of T7K appears to be a conserved feature amongst T7K homologues in Autographiviridiae, it does not appear to be a general feature of phage-mediated kinase signalling"

Are there structural or sequence similarities among kinases from diverse phages that suggest a conserved mechanism?

We thank the reviewer for this comment that led to the realization that homologue kinases are found outside of the *Autographiviridae* phage family, and that the hallmark of these kinases is a novel DFG-like DPV motif, which provides structural insights into the hyper-promiscuity of these kinases. This analysis resulted into a new figure and extensive analysis presented in the revised manuscript and briefly explained below.

To investigate whether we could identify any conserved features of T7K, we first searched for sequence homologues within viral (BFVD) and bacterial (Progenomes3) genome databases, and also within the genomes of the phages in the BASEL collection which we can experimentally interrogate. We identified 190 viral and 32 bacterial homologous proteins **(new Fig. 5b, new Supplementary Table 8)**. The vast majority of homologues were found in T7-like phages from the *Autographiviridae* family, but after careful quality control of the remaining sequences, we also found evidence for more distant homologues within a group of genomes representing two closely related jumbophages, as well as five homologues of putative bacterial origin, four of which seem to be encoded within prophages **(Extended Data Fig 9c)**.

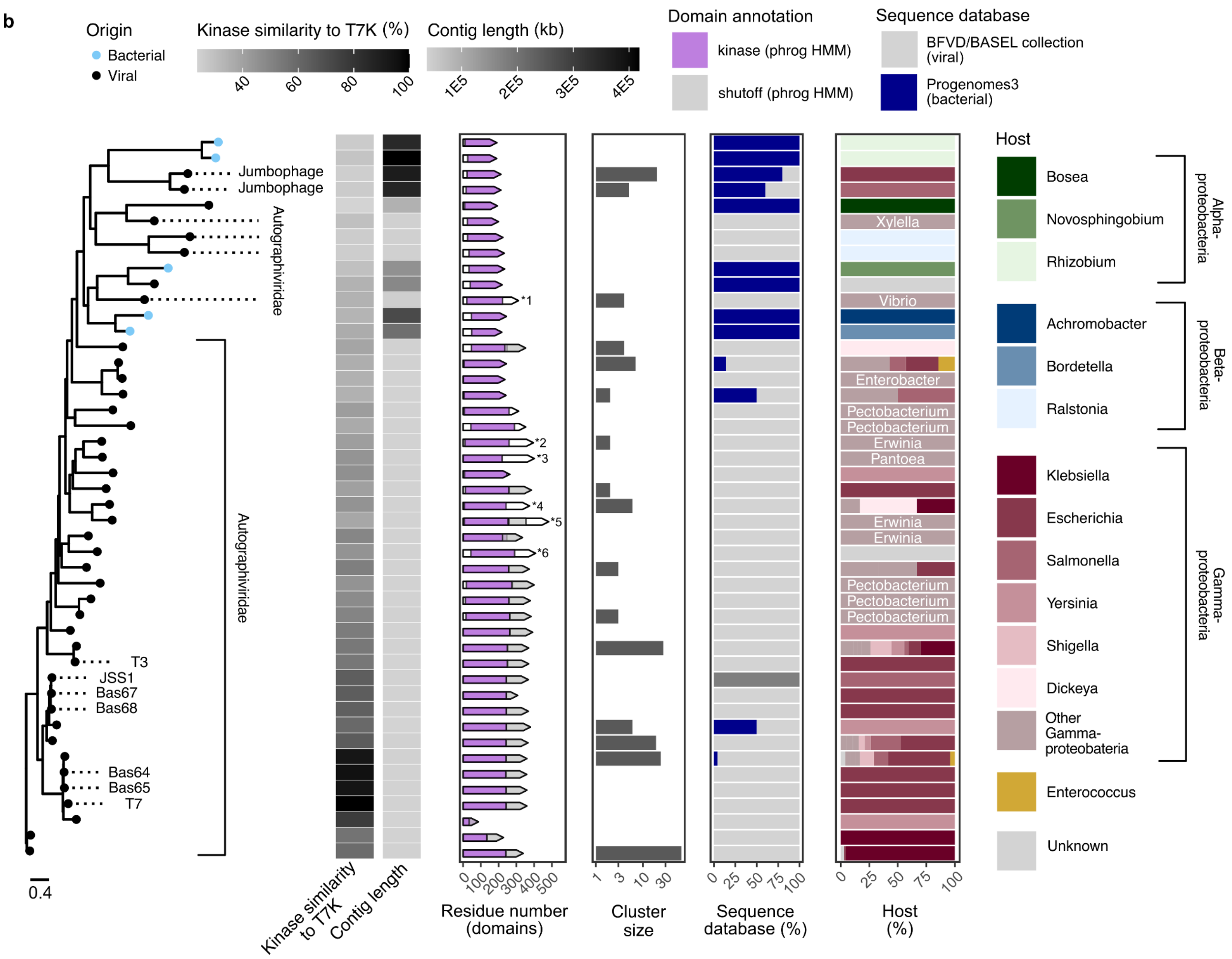

New Figure 5: Conserved hyperactivity and sequence features of T7K homologues. (b) *T7K homologues are common amongst Autographiviridae, but are also found in putative prophages and jumbophages with distinct domain architectures and host range. Phylogenetic tree of the clustered kinase domain sequences from putative T7K kinases found in viral and bacterial sequence databases (see Methods). We reduced sequence redundancy by clustering the kinase domains at 80% sequence identity into 46 representatives (see Supplementary Table 8 for all hits). Leaves labelled as "Jumbophage" represent T7K hits from Jumbophages resembling 'Salmonella phage Munch' and 'Escherichia phage G17'. Leaves with a blue tip represent homologues in putative prophages: they originate from bacterial genomes where a prophage-like region devoid of bacterial marker genes but containing a T7K-like protein (Extended Data Fig. 9b) is bordered by several bacterial marker genes. The labels 1-6 next to the gene arrows represent putative homologues with a divergent C-terminal end that were further structurally investigated (Extended Data Figure 9c).*

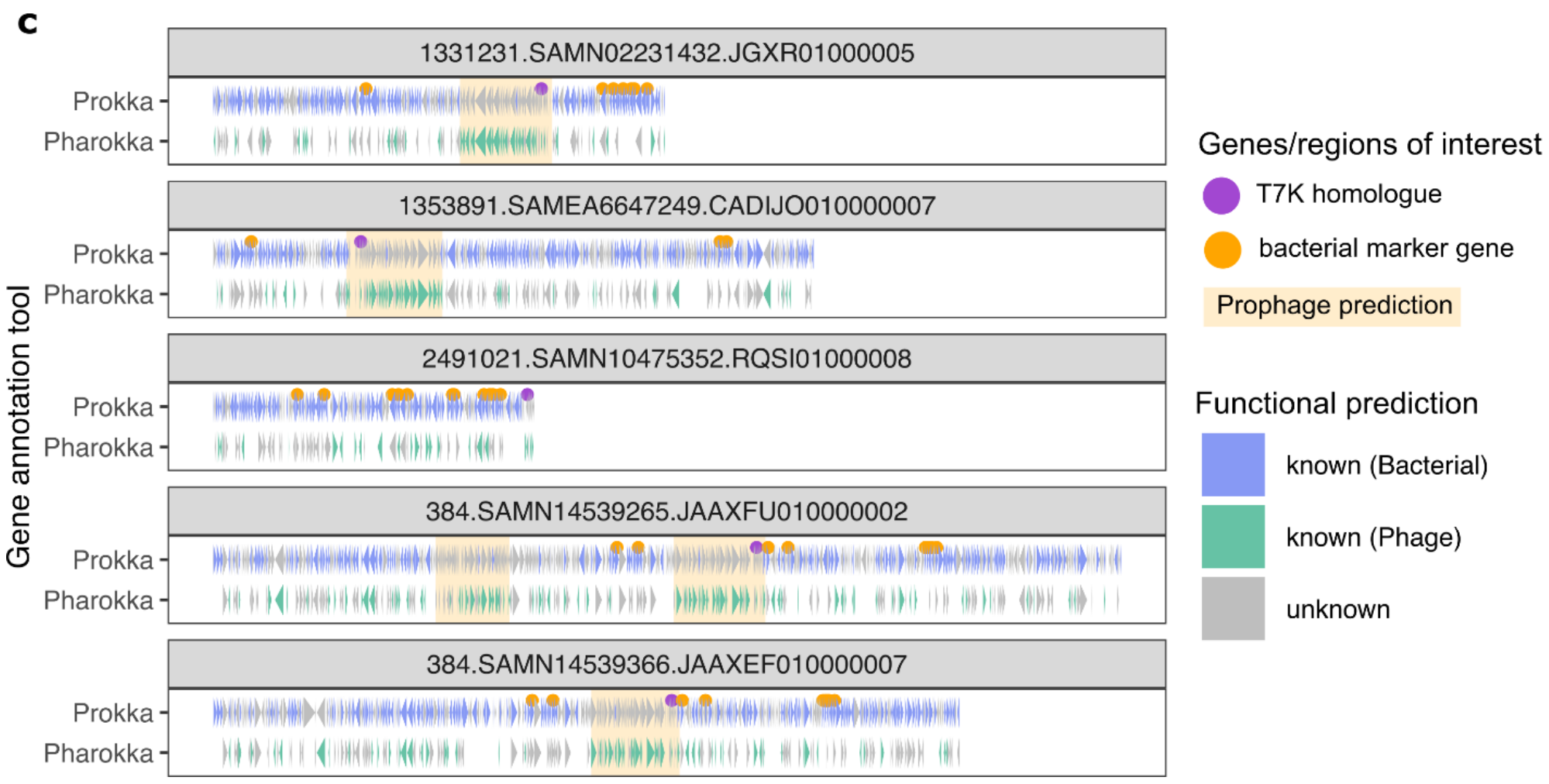

New Extended Data Figure 9c: *Quality control of putative prophage regions carrying a T7K homologue. Open reading frames and functional predictions were identified using both a bacteria-focused genome annotation tool (Prokka) and phage-focused genome annotation tool (Pharokka). Regions in yellow correspond to predicted prophage regions called by the PHASTEST tool. The purple dot highlights the locus encoding a T7K homologue; orange dots highlight bacterial marker genes identified with gtdb-tk.*

When closely examining these sequences, it became evident that the vast majority of T7K homologues contained a highly conserved ITDP(V/L/I)S motif, with kinases from *Autographiviridae* phages having a highly conserved ITDPVS variant **(new Fig. 5c)**. Closer inspection led to the realization that this a novel, DFG-like motif (DPV) which is positioned within the ATP-binding active site of the kinase. Furthermore, we identified kinase motif variants (ITDPLS in the jumbophage kinase motif) and a lower degree of conservation at the V site (V/L/I) in the small number of putative prophage kinases we identified.

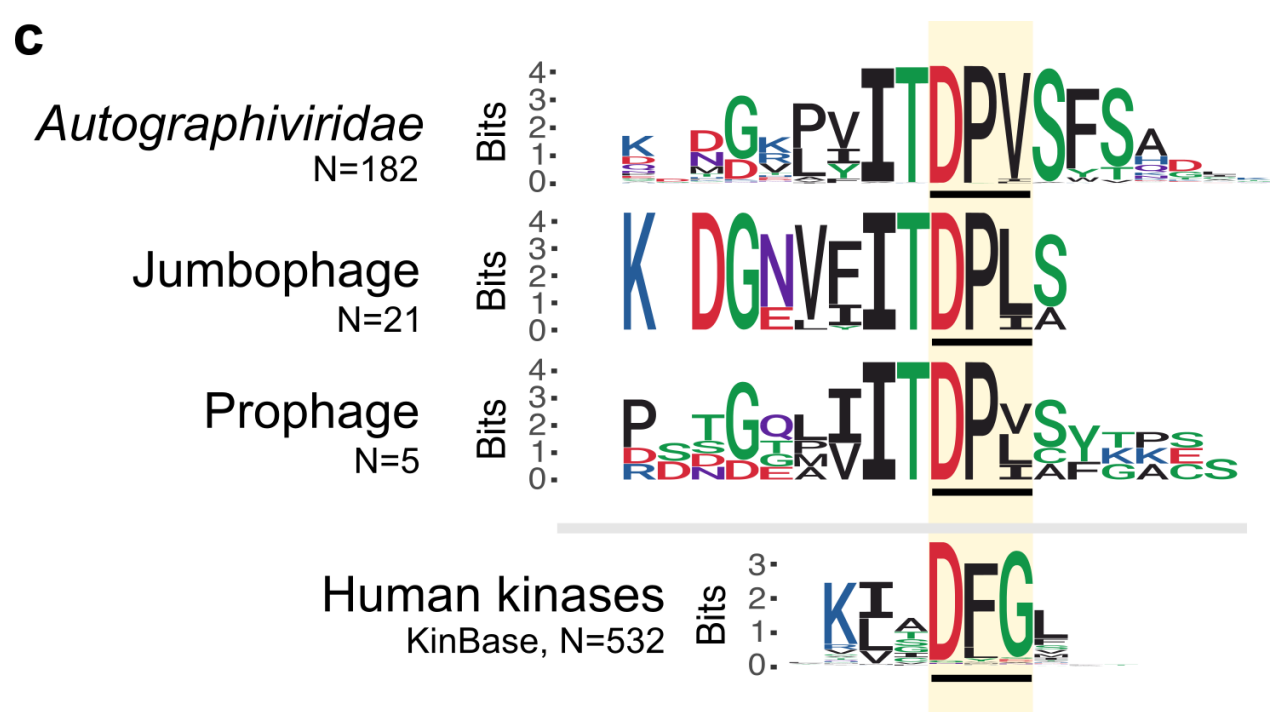

New Figure 5c: *T7K homologues contain a distinct, DFG-like motif. LOGO motif plots show the conservation of residues surrounding the kinase active site in T7K homologues from different origins - from Autographiviridae, and from Jumbophages or prophages (as both described above). The DPV motif corresponds to residues 213-215 in T7K. Conservation of residues surrounding the DFG motif in human kinase domain sequences (downloaded from KinBase94) are also shown for reference.*

The well-studied DFG motif in eukaryotic kinases is a highly conserved motif that undergoes two distinct conformational states controlling the accessibility to ATP, and hence are crucially involved in controlling the activity of the kinase. Structural comparison of the T7K AlphaFold model with experimental kinase structures in these two DFG conformations revealed that the DPV residues in the T7-like motif adopt a structure which strongly resembles the DFG-in (active) conformation **(new Fig. 5c)**. Together, these provide some insights towards plausible biochemical mechanisms of T7K's hyperactivity. Future studies could investigate the biochemistry of these enzymes further, although we believe it is outside the scope of the current manuscript.

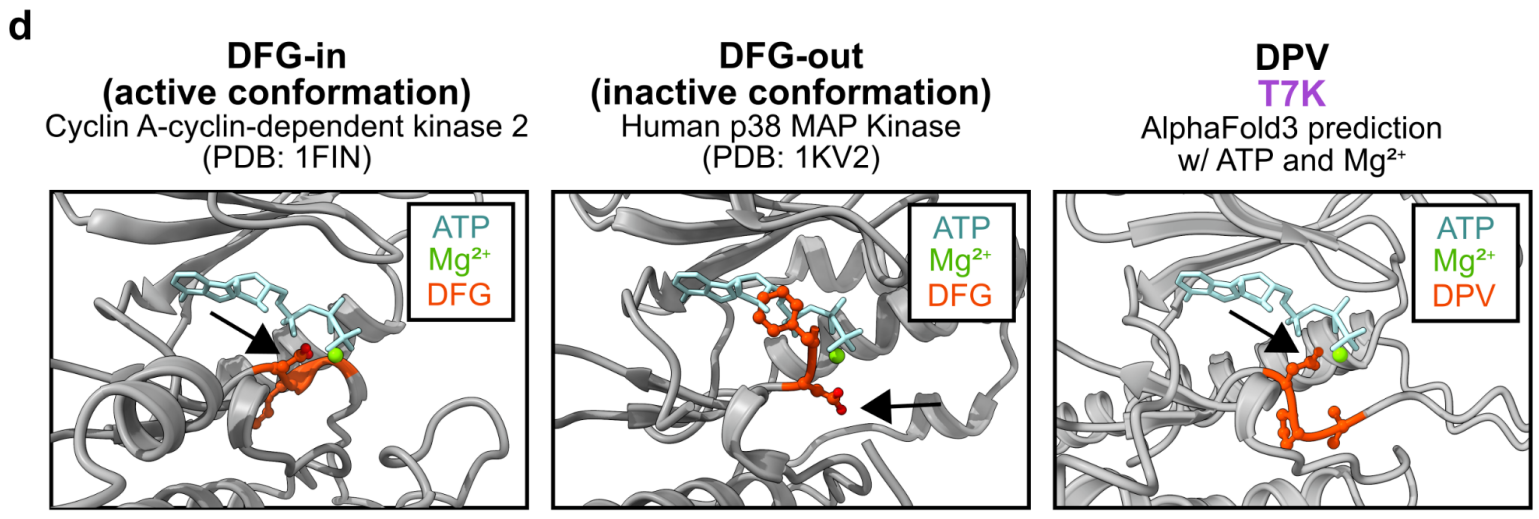

New Figure 5d: *The T7K DPV motif aligns with the DFG motif structural position in the kinase active site and mimics its active-state conformation. Zoomed-in views of the ATP-binding site after aligning DFG-in and DFG-out structures (PDB: 1FIN, 1KV2) to the AlphaFold3 T7K -* Mg^{2+} *- ATP model (Supplementary Fig. 13c). DFG/DPV motif residues are shown in orange and with sidechains, with arrows indicating the position of the aspartate (D) residue.*

Action:

- We introduce several new figures describing these findings **(Fig. 5b, 5c, 5d, Extended Data 9, Supplementary Table 8)**.
- We report our findings on the identification of T7K homologues and the new discovery of the novel DFG-like DPV motif in a new section of the text entitled **"***T7K homologues are found outside Autographviridiae and defined by a unique DFG-like motif*". We also include it here verbatim:

> *"To further understand the evolutionary origins and diversity of T7K, we undertook a comprehensive search for putative homologues in both viral and bacterial genome databases. We found 190 proteins in viral databases (BFVD, BASEL phage collection) and 32 proteins in the bacterial genome database Progenomes3 with significant sequence similarity to the kinase domain of T7K (Fig. 5b). To remove sequence redundancy, we clustered these proteins at 80% sequence similarity, ending up with 46 representative clusters (Fig. 5b).*
>
> *Most viral entities with a putative T7K homologue belonged to the Autographiviridae, the same family that the T7 phage belongs to, and had similar genome sizes to that of the T7 reference genome (Fig. 5b, Supplementary Table 8). This group contained the experimentally validated phages from the BASEL collection (Bas64, Bas65, Bas66(T3K12), Bas68), which were also among the closest homologues of T7K. Interestingly, early proteins in nearly all Autographiviridae phages with T7K homologues were depleted in S/T/Y residues (Extended Data Fig. 9a), suggesting that the kinase's promiscuity exerts positive selection on these genomes. Autographiviridae variants retained for the most part the domain architecture of T7K, with the shutoff domain following the kinase domain. We could not find evidence of different domains in those few variants in which the C-terminal sequence had diverged further (labelled 1-6 in Fig. 5b, Extended Data Fig. 9b). Further distant T7K homologues included two variants stemming from jumbophages (~400 kb genome size). The two clusters representing these variants included proteins of known lytic jumbophages: Salmonella phage Munch in one clade and Escherichia phage G17 in the other. Interestingly, both phages are frequently found as part of bacterial genome sequences – either due to contamination or due to some unresolved aspect of their lytic cycle84. This likely explains why most members of these two clusters were detected in the Progenomes3 database rather than from BFVD (Fig. 5b). Finally, after accounting for likely viral contamination in the bacterial database (see Methods), we only sporadically detected T7K homologues within bacterial contigs (N=5) (blue leaf nodes in Fig. 5b). Those represented single instances in phylogenetically diverse bacterial hosts and in most cases were part of a predicted prophage (N=4) (Extended Data Fig. 9c). Both jumbophage and the kinases of putative bacterial-origin had diverged further from the T7K, and had also lost the shutoff domain (Fig. 5b). The function and promiscuity of such more divergent kinases remains to be addressed in the future.*
>
> *In most eukaryotic kinases, catalytic activity is partially governed by residues within the conserved DFG motif. Sequence analyses of T7K homologues revealed a distinct but highly conserved DPV motif in the Autographiviridae homologues, with the valine being absent or less conserved in the jumbophage and prophage-encoded T7K homologues (Fig. 5c). The canonical DFG motif occurs at the beginning of the kinase activation loop and has two major structural conformations - DFG-in and DFG-out85 (Fig. 5d). In the DFG-in conformation, the aspartate coordinates with Mg2+ so that ATP is positioned well for hydrolysis, whereas in the DFG-out state the aspartate is displaced away from the Mg2+ by the rotated phenylalanine, leading to loss of this interaction and rendering the kinase into a catalytically incompetent conformation (Fig. 5d). The DPV motif occupies the same structural location within the AlphaFold3-modeled T7K structure as the DFG residues in ATP-binding sites of experimentally resolved kinase structures (Fig. 5d, Extended Data Fig. 9d), but lacks the phenylalanine residue which displaces ATP-binding in DFG-out conformation. To better understand how this change can be accommodated in the two conserved conformation states, we compared the T7K AlphaFold prediction with experimental (X-ray) structures representing the DFG-in and DFG-out conformations (Fig. 5d). These comparisons supported that the T7K and its binding pocket are much more similar to the DFG-in conformation instead of the DFG-out conformation. They also indicated that the aspartate from DPV is able to maintain its geometry for a productive coordination with the Mg2+ - ATP complex (Fig. 5d, black arrow), and that the proline has potential to support this arrangement and maintain a favorable aspartate - Mg2+ - ATP interaction, enabling higher activity from this kinase. Of note, the two previously reported T7K auto-phosphorylation sites are found immediately downstream of the DPV motif, with S216 (reported inhibitory site) being conserved in all variants, and S218 being prevalent in the Autographiviridae variants (Fig. 5c), and are therefore positioned to affect the activation loop. Overall, it seems plausible that the DPV motif maintains T7K in a constitutively active form by locking T7K in the DFG-in-like form and not allowing it to flip into any other conformation such as DFG-out. Autophosphorylation sites give a way to deactivate this highly processive kinase."*

Are phage kinases found with an array of other protein domains that might suggest what they could be targetting, like in the current example of DNA/RNA binding region ?

To investigate whether the T7K kinase domain was ever found with other domains, we examined the domain composition of the T7K sequence homologues using HMMs specific for either the kinase or the shutoff domains **(new Fig. 5b)**. This revealed that the vast majority of kinases from T7-like phages contained the same architecture as T7K (containing both the kinase and the shutoff domain), whilst prophage and jumbophage kinases appeared to only contain the kinase domain. The lack of shutoff domain and hence DNA-targeting ability in jumbophage kinase homologues is intriguing and could potentially be related to their unique infection cycle and will be an exciting topic for future studies. Similarly, the lack of a shut-off domain in prophages will be interesting to investigate in the future and could potentially shed light on how such kinases are domesticated.

The Figure 5b panel is included in the above responses already.

We also examined the viral proteins where there was significant extension of sequence beyond the kinase domain but where no shutoff domain could be detected. However, we could not find any sequence-based (InterProScan) annotations for the function of these sequences **(Extended Data Fig. 9b)**. Hence, we currently have no evidence of any other functional domains associated with the T7K kinase domain that would suggest functionalization towards different molecules within the cell.

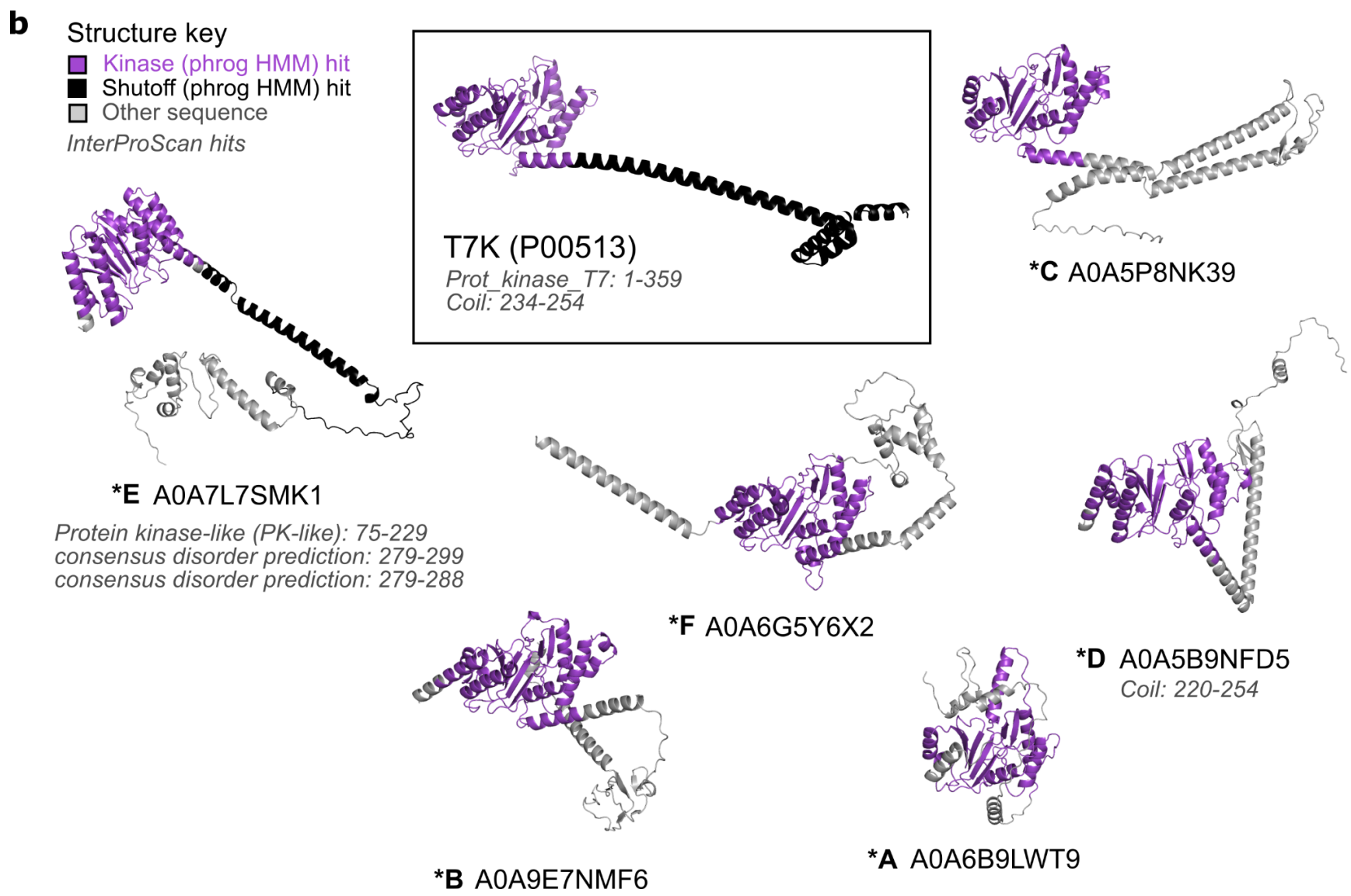

Extended Data Figure 9: (b) *AlphaFold3 predictions for clade-representing homologues from Fig. 5b with non-canonical domain architectures. These proteins had kinase (phrog HMM) matches (purple residues) but also had a significant stretch of their sequence without hits to the shutoff (phrog HMM) (grey residues). T7K is also shown for reference in the box. A0A7L7SMK1 also contained a shutoff (phrog HMM) hit (black residues). Regions corresponding to hits from InterProScan5 are shown in italics below the protein name (UniProt accessions). The numeric labels next to the structures are also indicated next to the gene arrows in Figure 5b.*

Action:

- We include the new analysis and findings on T7K homologue domain architectures in the new **Fig. 5b** and **Extended Data Fig. 9b.**
- We report the findings on the domain composition in the new Results section "*T7K homologues are found outside Autographviridiae and defined by a unique DFG-like motif*", which we already included verbatim in our above response.

A broader phylogenetic analysis and comparison with kinases found in prophage regions could be valuable.

Most of the T7K homologues we found within bacterial genomes (Progenomes 3 database) turned out to be phage contamination upon closer inspection (27/32) – were part of contigs that were of similar size and conserved/syntenic gene content as lytic *Autographviridiae* or the new clades of jumbophages found to contain T7K homologues **(Fig. 5b)**. Interestingly the latter have been both recently reported to be frequently found as part of bacterial genomes – either due to contamination or unknown biology of their lytic cycle[6].

The remaining five T7K homologues found in bacterial genomes represented single instances in phylogenetically diverse bacterial hosts and in most cases were part of a predicted prophage (N=4) **(Extended Data Fig. 9c)**. Because of their sporadic presence, and the significant phage contamination within the available sequences of bacterial isolate genomes, we refrain of making conclusive statements for the presence of T7K homologues in bacterial genomes, and raise the possibility that this may stem from genome assembly artefacts in Discussion.

We also looked into the sequence features of these putative bacterial (prophage) kinases, and although it is difficult to make too conclusions based on the small number of sequences found, it appears that they have a less conserved valine in the active site motif (ITDP(V/L/I)S) and also are only found in the kinase-only domain configuration **(Fig. 5c)**.

Extended Data 9b is included in the above responses already
The Figure 5b and 5c panels are included in the above responses already.

Action:

- We report the findings on the discovery of homologues in prophage-encoded regions in the new Results section "*T7K homologues are found outside Autographviridiae and defined by a unique DFG-like motif*":

  "To further understand the evolutionary origins and diversity of T7K, we undertook a comprehensive search for putative homologues in both viral and bacterial genome databases. We found 190 proteins in viral databases (BFVD, BASEL phage collection) and 32 proteins in the bacterial genome database Progenomes3 with significant sequence similarity to the kinase domain of T7K (Fig. 5b). To remove sequence redundancy, we clustered these proteins at 80% sequence similarity, ending up with 46 representative clusters (Fig. 5b).

  Most viral entities with a putative T7K homologue belonged to the Autographiviridae, the same family that the T7 phage belongs to, and had similar genome sizes to that of the T7 reference genome (Fig. 5b, Supplementary Table 8). This group contained the experimentally validated phages from the BASEL collection (Bas64, Bas65, Bas66(T3K12), Bas68), which were also among the closest homologues of T7K. Interestingly, early proteins in nearly all Autographiviridae phages with T7K homologues were depleted in S/T/Y residues (Extended Data Fig. 9a), suggesting that the kinase's promiscuity exerts positive selection on these genomes. Autographiviridae variants retained for the most part the domain architecture of T7K, with the shutoff domain following the kinase domain. We could not find evidence of different domains in those few variants in which the C-terminal sequence had diverged further (labelled 1-6 in Fig. 5b, Extended Data Fig. 9b). Further distant T7K homologues included two variants stemming from jumbophages (~400 kb genome size). The two clusters representing these variants included proteins of known lytic jumbophages: Salmonella phage Munch in one clade and Escherichia phage G17 in the other. Interestingly, both phages are frequently found as part of bacterial genome sequences – either due to contamination or due to some unresolved aspect of their lytic cycle84. This likely explains why most members of these two clusters were detected in the Progenomes3 database rather than from BFVD (Fig. 5b). Finally, after accounting for likely viral contamination in the bacterial database (see Methods), we only sporadically detected T7K homologues within bacterial contigs (N=5) (blue leaf nodes in Fig. 5b). Those represented single instances in phylogenetically diverse bacterial hosts and in most cases were part of a predicted prophage (N=4) (Extended Data Fig. 9c). Both jumbophage and the kinases of putative bacterial-origin had diverged further from the T7K, and had also lost the shutoff domain (Fig. 5b). The function and promiscuity of such more divergent kinases remains to be addressed in the future."

- We report our findings on the domain architecture and motif variant in putative prophage homologues in the new **Fig. 5b and c** and **Extended Data Fig. 9c,** and in associated text in the same Results subsection.

  "Most viral entities with a putative T7K homologue belonged to the Autographiviridae, the same family that the T7 phage belongs to, and had similar genome sizes to that of the T7 reference genome (Fig. 5b, Supplementary Table 8). This group contained the experimentally validated phages from the BASEL collection (Bas64, Bas65, Bas66(T3K12), Bas68), which were also among the closest homologues of T7K. Interestingly, early proteins in nearly all Autographiviridae phages with T7K homologues were depleted in S/T/Y residues (Extended Data Fig. 9a), suggesting that the kinase's promiscuity exerts positive selection on these genomes. Autographiviridae variants retained for the most part the domain architecture of T7K, with the shutoff domain following the kinase domain. We could not find evidence of different domains in those few variants in which the C-terminal sequence had diverged further (labelled 1-6 in Fig. 5b, Extended Data Fig. 9b). Further distant T7K homologues included two variants stemming from jumbophages (~400 kb genome size). The two clusters representing these variants included proteins of known lytic jumbophages: Salmonella phage Munch in one clade and Escherichia phage G17 in the other. Interestingly, both phages are frequently found as part of bacterial genome sequences – either due to

contamination or due to some unresolved aspect of their lytic cycle84. This likely explains why most members of these two clusters were detected in the Progenomes3 database rather than from BFVD (Fig. 5b). Finally, after accounting for likely viral contamination in the bacterial database (see Methods), we only sporadically detected T7K homologues within bacterial contigs (N=5) (blue leaf nodes in Fig. 5b). Those represented single instances in phylogenetically diverse bacterial hosts and in most cases were part of a predicted prophage (N=4) (Extended Data Fig. 9c). Both jumbophage and the kinases of putative bacterial-origin had diverged further from the T7K, and had also lost the shutoff domain (Fig. 5b). The function and promiscuity of such more divergent kinases remains to be addressed in the future.

In most eukaryotic kinases, catalytic activity is partially governed by residues within the conserved DFG motif. Sequence analyses of T7K homologues revealed a distinct but highly conserved DPV motif in the Autographiviridae homologues, with the valine being absent or less conserved in the jumbophage and prophage-encoded T7K homologues (Fig. 5c). The canonical DFG motif occurs at the beginning of the kinase activation loop and has two major structural conformations - DFG-in and DFG-out85 (Fig. 5d). In the DFG-in conformation, the aspartate coordinates with Mg2+ so that ATP is positioned well for hydrolysis, whereas in the DFG-out state the aspartate is displaced away from the Mg2+ by the rotated phenylalanine, leading to loss of this interaction and rendering the kinase into a catalytically incompetent conformation (Fig. 5d). The DPV motif occupies the same structural location within the AlphaFold3-modeled T7K structure as the DFG residues in ATP-binding sites of experimentally resolved kinase structures (Fig. 5d, Extended Data Fig. 9d), but lacks the phenylalanine residue which displaces ATP-binding in DFG-out conformation. To better understand how this change can be accommodated in the two conserved conformation states, we compared the T7K AlphaFold prediction with experimental (X-ray) structures representing the DFG-in and DFG-out conformations (Fig. 5d). These comparisons supported that the T7K and its binding pocket are much more similar to the DFG-in conformation instead of the DFG-out conformation. They also indicated that the aspartate from DPV is able to maintain its geometry for a productive coordination with the Mg2+ - ATP complex (Fig. 5d, black arrow), and that the proline has potential to support this arrangement and maintain a favorable aspartate - Mg2+ - ATP interaction, enabling higher activity from this kinase. Of note, the two previously reported T7K auto-phosphorylation sites are found immediately downstream of the DPV motif, with S216 (reported inhibitory site) being conserved in all variants, and S218 being prevalent in the Autographiviridae variants (Fig. 5c), and are therefore positioned to affect the activation loop. Overall, it seems plausible that the DPV motif maintains T7K in a constitutively active form by locking T7K in the DFG-in-like form and not allowing it to flip into any other conformation such as DFG-out. Autophosphorylation sites give a way to deactivate this highly processive kinase."

- We significantly expanded our discussion of these new findings on T7K homologues in the following sentences:

"In this work, we report that T7K acts as a hyper-promiscuous, dual-specificity kinase, which can phosphorylate any protein inside the infected cell. The substrate range of T7K exceeds that of any kinase studied before, and even that of all human kinases put together, with no motif whatsoever next to the phosphorylated S/T/Y residues. It likely achieves this high activity through its DFG-like DPV motif, which has the potential to maintain the kinase active site in an open, constitutively-active form. This loose cannon kinase gains specificity towards proteins binding DNA or different RNA species via its C-terminal shutoff (SO) domain that binds DNA, and manages to stoichiometrically phosphorylate DNA and RNA-binding proteins (the latter possibly through the tight coupling between transcription and translation in prokaryotes), within its short window of activity. The phage uses this stoichiometric phosphorylation to deactivate proteins sensing or attacking its DNA, and possibly its RNA. To mitigate the dangers of such uncontrolled phosphorylation, T7 inactivates the kinase within 6 mins after infection via autophosphorylation. The timing of this inactivation seems to be regulated at least partially through the SO domain, likely through its interactions with the DNA which reduces the pool of free floating T7K molecules. To which degree the SO domain acts directly or via the kinase domain in defense inactivation remains to be elucidated. The phage has also evolved to deplete S/T exposed residues from the surface of its early proteins. Such kinase activity has not been reported to our knowledge before, and sets a new paradigm of how phosphorylation can be used in living organisms – to deactivate molecular machines. Strikingly, and despite its vast impact on the cellular proteome, the role of the T7K has remained elusive for almost 50 years. This is because earlier tools lacked the sensitivity for identifying phosphorylated substrates, but also due to absence of strong phenotypes for the T7K mutant – having been tested in lab strains with poor phage defense repertoires.

Close homologues of T7K are almost exclusively found in phages (Supplementary Table 2, Fig. 5b) – the few sporadic instances in bacterial genomes (prophage sites) would need to be further verified to exclude genome assembly artefacts. This suggests that the hyper-promiscuity of T7K phosphorylation could be an ancestral strategy that is incompatible with the selective S/T/Y signaling found in complex organisms. In the future, it would be interesting to study the activity of the more distant T7K homologues (jumbophage and prophage variants), and their relation to more distant dual-specificity kinases found in bacteria and eukaryotes, as this may provide insights into how such kinases may have become "domesticated" by tighter control of their activity and/or by gaining or changing specificity."

The authors could additionally perform the phosphoproteomics studies in the presence of other phage encoded kinases to determine the degree of generalization for the proposed mechanism.

As described above, we introduce several new phosphoproteomics datasets studying T7K homologues in BASEL phages (Bas64, Bas65, Bas66, Bas68). We also study another phage-encoded kinase within P1*vir* **(Figure 5a)**. Additionally, we performed expression proteomics on the same samples to confirm successful infection of *E. coli* K12 with each phage **(Extended Data Fig. 8a)** and expression of the relevant kinases at the sampled time-points (T=5 or T=10 for *Autographviridae,* T=45 for P1*vir*) **(Extended Data Fig. 8b)**.

This revealed that not all phage kinases have the same hyperactivity and promiscuity as T7K homologues (Bas64, Bas65, Bas66(T3K12), Bas68), although they can still produce distinct changes to the phosphoproteome **(Extended Data Fig. 8c).**

The Figure 5a panel is included in the above responses already.
The Extended Data Figure 8 panel is included in the above responses already.

Action:

- We describe these new phosphoproteomics datasets in the new **Figure 5a** and **Extended Data 8** and under the new Results section subheading entitled "*Hyperpromiscuous kinase activity is conserved among T7K homologues*", which is also included verbatim in the response above.

The authors mention a related study with a Salmonella phage JSS1 but is unclear if that study was done with the same degree of sensitivity in the MS profiling.

We tried to obtain and then study the *Salmonella* phage JSS1 (Chen group, Jiang et. al.[1]). Altough we received the phage, we were not able to import the matching host strain due to biosafety regulations regarding imports from China to Germany and other EC countries (no BSL2 material allowed). We could also not find this host anywhere else, and all *Salmonella* strains we tested were not infected by JSS1.

However, when doing the phylogenetic analyses to investigate T7K homologues **(new Fig. 5b),** we identified that the Bas68-encoded kinase was a very close homologue to the JSS1_004 kinase (kinase domain sequence identity of 95%). We note that the degree of hyperphosphorylation induced by Bas68 infection resembles the clear hyperactivity of the other tested *E. coli* phages with T7K homologues **(new Fig. 5a)**, and we assume that any differences in the apparent hyperactivity reported in Jiang et. al [1] for JSS1 are due to technical reasons (sensitivity of phosphoproteomics), however we cannot state that conclusively and have avoided doing so in the manuscript.

The new Figure 5a and b panels are included in the above responses already.

Action:

- We include the following sentence pointing out the link between the kinases encoded by Bas68 and JSS1:

  "*Phosphoproteomes of the same samples revealed extensive phosphorylation triggered by the Autographiviridiae phages (Bas64, Bas65, Bas66(T3K12), Bas68 - all encode T7K homologues), similar to that observed for T7K, but not by P1vir* ***(Fig. 5a, Extended Data Fig. 8c, Supplementary Table 8)****. Among the tested phages, the kinase from Bas68 most closely resembles JSS1_004 from Salmonella phage JSS1 (95% sequence identity within the kinase domain)* ***(Fig. 5b)****, which was reported to be unspecific albeit to a lower degree (~20-fold lower phosphorylation) than T7K. Given their high similarity, and the kinase hyperpromiscuity across all other Autographiviridiae tested here, the reported difference in activity for T7K and JSS1_004 likely arises from a more sensitive phosphoproteomics approach used in this work.*"

1.2 - The manuscript would benefit from discussing the evolutionary pressures that might have driven the divergence of substrate specificity, possibly providing insights into how phage kinases evolved toward promiscuity. Having some phosphoproteomic data on divergent phage kinases would make such an analysis even stronger.

We agree with the reviewer and have provided more evidence regarding the evolution and divergence of kinase specificity and promiscuity. As described in our above response to **reviewer 1 comment #1.1**, we have collected new experimental data investigating the kinase activity of four divergent homologous kinases with between 55% (Bas66(T3K12)) and 95% (Bas64) sequence identity to the kinase-domain of T7K **(new Fig. 5a)**. We also studied an unrelated phage kinase encoded in P1*vir*, as detailed in **reviewer 1 comment #1.1**.

We also provide new sequence/structural analyses that hint towards how the DPV motif may contribute to kinase promiscuity **(new Fig. 5d),** and phylogenetic analyses that led to the discovery of homologues in jumbophages and potential prophage regions without the SO domain **(new Fig. 5b, 5c,** detailed in **reviewer 1 comment #1.1**) in relation to the potential for substrate specificity divergence.

All that being said, we also need to acknowledge that a full understanding of the divergence of such promiscuous kinases, the control of their activity and specificity, and their possible domestication would need to be the focus of several future studies.

The new Figure 5 with four panels are included in the above responses already.

Action:

- We report our new findings investigating new phages from the BASEL collection with phosphoproteomics in the new **Fig. 5a** and **Extended Data Fig. 8**. We report this in the text as follows:

> *"To investigate whether hyperpromiscuous kinase activity is common in phages, we searched for known protein kinases within the BASEL collection of E. coli lytic phages. We identified at least four other phages in the same family as T7 encoding T7K homologues (Autographiviridiae; Bas64, Bas65, Bas66(T3K12), Bas68), and the model phage P1vir which is known to encode the Doc kinase toxin. We collected a single time-point during infection of E. coli K12, choosing 10 minutes for the T7-like phages (except Bas68, where we collected 5 minutes post-infection due to its faster infection dynamics), and a later time-point for P1vir (45 min) due to its longer infection cycle. Expression proteomics confirmed that each phage's infection cycle had progressed, as the majority of their proteins, including the kinase of interest, could be detected (Extended Data Fig. 8a-b). Phosphoproteomes of the same samples revealed extensive phosphorylation triggered by the Autographiviridiae phages (Bas64, Bas65, Bas66(T3K12), Bas68 - all encode T7K homologues), similar to that observed for T7K, but not by P1vir (Fig. 5a, Extended Data Fig. 8c, Supplementary Table 8). Among the tested phages, the kinase from Bas68 most closely resembles JSS1_004 from Salmonella phage JSS1 (95% sequence identity within the kinase domain) (Fig. 5b), which was reported to be unspecific albeit to a lower degree (~20-fold lower phosphorylation) than T7K. Given their high similarity, and the kinase hyperpromiscuity across all other Autographiviridiae tested here, the reported difference in activity for T7K and JSS1_004 likely arises from a more sensitive phosphoproteomics approach used in this work.*
>
> *On the other hand, phosphoproteome investigations after infection with P1vir, which encodes an unrelated kinase, revealed that not all phage kinases are hyperpromiscuous. Despite its low expression during infection (Extended Data Fig. 8b), we could reproducibly detect the single known phosphorylation site of Doc kinase (elongation factor Tu 1; TufA-T383). P1vir infection produced a phosphoproteome profile distinct from those of uninfected samples, T7 infection and of T7 mutants (Extended Data Fig. 8c) - with 20 additional sites across 17 proteins identified reproducibly and uniquely during P1vir infection (Extended Data Fig. 8d, Supplementary Table 8), including three phage proteins. These new sites may represent undescribed substrates of the Doc kinase or a host response specific to P1vir infection."*

- We report our new findings on the discovery of the DPV motif in the new **Fig. 5c**. We describe this in the text as follows:

> *"In most eukaryotic kinases, catalytic activity is partially governed by residues within the conserved DFG motif. Sequence analyses of T7K homologues revealed a distinct but highly conserved DPV motif in the Autographiviridae homologues, with the valine being absent or less conserved in the jumbophage and prophage-encoded T7K homologues (Fig. 5c). The canonical DFG motif occurs at the beginning of the kinase activation loop and has two major structural conformations - DFG-in and DFG-out (Fig. 5d). In the DFG-in conformation, the aspartate coordinates with Mg2+ so that ATP is positioned well for hydrolysis, whereas in the DFG-out state the aspartate is displaced away from the Mg2+ by the rotated phenylalanine, leading to loss of this interaction and rendering the kinase into a catalytically incompetent conformation (Fig. 5d). The DPV motif occupies the same structural location within the AlphaFold3-modeled T7K structure as the DFG residues in ATP-binding sites of experimentally resolved kinase structures (Fig. 5d, Extended Data Fig. 9d), but lacks the phenylalanine residue which displaces ATP-binding in DFG-out conformation. To better understand how this change can be accommodated in the two conserved conformation states, we compared the T7K AlphaFold prediction with experimental (X-ray) structures representing the DFG-in and DFG-out conformations (Fig. 5d). These comparisons supported that the T7K and its binding pocket are much more similar to the DFG-in conformation instead of the DFG-out conformation. They also indicated that the aspartate from DPV is able to maintain its geometry for a productive coordination with the Mg2+*

- ATP complex (Fig. 5d, black arrow), and that the proline has potential to support this arrangement and maintain a favorable aspartate - Mg2+ - ATP interaction, enabling higher activity from this kinase. Of note, the two previously reported T7K auto-phosphorylation sites are found immediately downstream of the DPV motif, with S216 (reported inhibitory site) being conserved in all variants, and S218 being prevalent in the Autographiviridae variants (Fig. 5c), and are therefore positioned to affect the activation loop. Overall, it seems plausible that the DPV motif maintains T7K in a constitutively active form by locking T7K in the DFG-in-like form and not allowing it to flip into any other conformation such as DFG-out. Autophosphorylation sites give a way to deactivate this highly processive kinase."

- We include the following sentences to discuss these findings with explicit relation to divergence of substrate specificity, especially relating:

*"Close homologues of T7K are almost exclusively found in phages **(Supplementary Table 2, Fig. 5b)** – the few sporadic instances in bacterial genomes (prophage sites) would need to be further verified to exclude genome assembly artefacts. This suggests that the hyper-promiscuity of T7K phosphorylation could be an ancestral strategy that is incompatible with the selective S/T/Y signaling found in complex organisms. In the future, it would be interesting to study the activity of the more distant T7K homologues (jumbophage and prophage variants), and their relation to more distant dual-specificity kinases found in bacteria and eukaryotes, as this may provide insights into how such kinases may have become "domesticated" by tighter control of their activity and/or by gaining or changing specificity. Other S/T kinases found in prophage regions of bacteria and known to be involved in phage defense such as Stk2 have also been suggested to phosphorylate a broader substrate clientele than currently appreciated.*

2 - The manuscript acknowledges overlap with a recent paper (Jiang et al. Nature Microbiology 2024). It seems to me that the current study proposes a more general mechanism of a promiscuous kinase that can target a large number of different defence systems. Besides this, the level of technical detail and analysis of diverse lab strains done here seem much more in depth than Jiang et al. Again here, the current study would be more differentiated if the authors could study the generality of their findings across other phage kinases through computational and experimental approaches.

Thank you for acknowledging the strength and uniqueness of the manuscript. We agree and also firmly believe that the mechanistic depth of our study and the unique observation and characterization of the unprecedented phosphorylation event upon T7K infection clearly sets this study apart. Addressing the constructive comments from all reviewers have even further broadened and strengthened our study. As described throughout our above responses to **reviewer 1 comment #1**, we have introduced several new experimental (phosphoproteomics) and computational (search for homologues, phylogenetics, motif discovery, domain architecture, structural investigations) analyses that describe the generalizability of our findings of T7K kinase's promiscuity and hyperactivity – including very likely to that of the JSS1 kinase

---

Minor Concerns
- line 107 – "Notably, no close homologues of T7K were found outside of phages" – what does "close" here mean. As described above, it would have been nice to have a better sense of the diversity of phage kinases and how they relate to other ST or STY kinases.

Close homologues in this context refers to protein sequences within the same UniRef 50 cluster, which are UniProt proteins with at least 50% sequence identity to the full length T7K sequence. We have removed reference to these clusters and also the original PHROG-based phylogenetic tree, which was built using sequences curated in the PHROG database as phrog_2828 (and supplemented with JSS1_004 kinase from *Salmonella* phage JSS1). We have replaced them with a more in-depth analysis, which is now reported in the new **Fig. 5b**. This analysis involved searching for sequence-based homologues in genome databases using hmm models built from the kinase domain in phrog_2828 members. This analysis revealed further distant homologues in jumbophages and possibly also in prophage regions in bacterial genomes (isolated cases).

The new Figure 5b panel is included in the above responses already.

To give the readers a better sense of how T7K relates to other ST and STY kinases, we also include comparison of the newly discovered DPV motif with the DFG motif in human kinases at both sequence **(new Fig 5c)** and structural levels **(new Fig. 5d)**.

The new Figure 5c and 5d panels are included in the above responses already.

Action:

- The original PHROG-based phylogenetic tree and reference to UniRef50 clusters were removed and replaced with more sophisticated analysis in the new **Fig. 5b**.
- Kinase motif conservation for T7K homologues and human kinases is now shown in the new **Fig. 5c.**

- The large degree of phosphorylation of the host proteins is very interesting and it is surprising that it does not generally lead to a massive disruption of protein function. Are the host protein phosphorylation sites mostly occurring in regions that are predicted to be accessible ?

We investigated whether the surface accessible residues within host proteins were more likely to be phosphorylated using phosphosites identified through the TMT time-course analysis **(Extended Data Fig. 2d)**. This was the case, with phosphorylated S/T/Y residues exhibiting significantly higher residue surface accessibilities in AlphaFold protein structural models. This phosphorylation of mostly accessible residues is matching the short window of activity of T7K (for 5 mins), during which the majority of available proteome comes from already folded proteins rather than nascent/newly synthesized (20-25 mins doubling time).

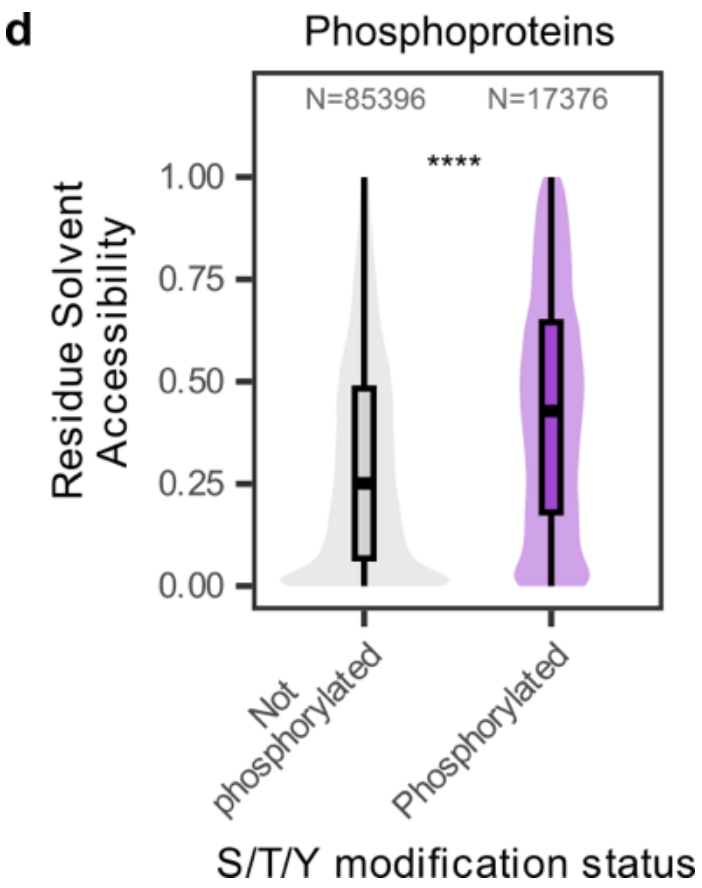

Extended Data Figure 2d: *Residue surface accessibility of S/T/Y residues within host phosphoproteins (proteins found phosphorylated at at least one site in the TMT analysis) subsetted by their phosphorylation status in the TMT analysis. Note: non-unique peptides were included for this analysis. The distributions were compared with unpaired t-tests (**** meaning p-value ≤ 0.0001). N= indicates the number of residues considered in each distribution.*

We wish to note that although the vast majority of soluble proteins are being modified to some degree by T7K, which would imply massive disruption of protein function, only a small proportion are being modified with a very high stoichiometry. Therefore, the vast majority of proteins have a large pool of unmodified proteoforms, and can function as usual (see e.g. Peters et al. [7], Mateus et al. [8], where it is shown that essential proteins are predominantly regulated in activity, not expression as they already exist in excess).

Action:

- We include a new figure describing this analysis **(Extended Data Fig. 2d)**, and introduce it in the main text with the following sentence:

> *Many host proteins were phosphorylated to high density (fraction of detectable S, T and Y residues that were phosphorylated), showcasing the processivity and promiscuity of T7K. This includes all 11 previously reported T7K-substrates, which were amongst the most abundant proteins in the cell **(Fig. 1g)**. As expected from the cytoplasmic action of the T7K, cell envelope and membrane proteins were largely depleted of phosphorylation **(Supplementary Table 3)**. Furthermore, modified sites within phosphorylated proteins were significantly more surface accessible **(Extended Data Fig. 2d)**, indicating T7K's activity on mature, folded proteins.*

- line 147 to 149 – The authors suggest that early phage proteins are perhaps selected to avoid having phosphorylable residues. This is an interesting idea but not really pursued in the manuscript. This is not something that I feel is very important but the authors could have tried to show that phages carrying related kinases have such sequence selection pressures to avoid phosphorylation of page proteins.

To understand whether phages encoding T7K might have been under selective pressure to minimize phosphorylatable residues in their proteome, we compared the frequency of S, T and Y residues in the proteomes of *Autographiviridae* relative to their approximate expression time during infection. We reasoned that, if such selective pressure were to exist, that early proteins would be under stronger selective pressure to avoid these residues as they would be co-expressed with the kinase and therefore have a depleted fraction.

To do this, we investigated all *Autographiviridae* with an identified T7K homologue (from both the BASEL collection as well as BFVD) and approximated membership of their genes in early, middle and late gene classes by transferring the boundaries of T7's early/middle/late genes by identifying homologues to T7 genes. We then calculated the cumulative fraction of S, T and Y residues in each of the three gene groups **(new Extended Data Fig. 9a)**. The plot clearly shows that the vast majority of *Autographiviridae* proteomes show a clear monotonic relationship between the fraction of S, T and Y expression time, with earlier genes having less of those residues, followed by middle and late genes

a

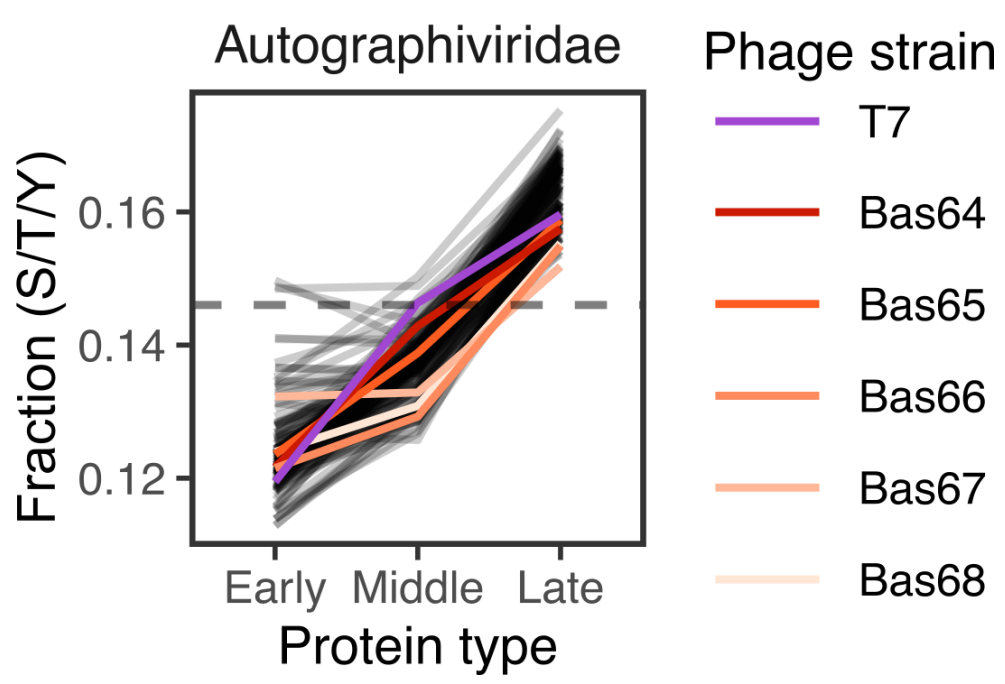

New Extended Data Figure 9a: *Cumulative fraction of serine (S), threonine (T) and tyrosine (Y) in early, middle and late genes over all Autographiviridae genomes with a T7-like kinase. We defined the boundaries of early, middle and late genes according to the boundary genes in 52 and transferred the information to other Autographiviridae by identifying homologues of key genes at T7 early/middle/late group boundaries using BLAST.*

Action:

- The new results showing a bias in early, middle, late proteins in the Autographiviridae with T7K-homologues is shown in the new **Extended Data Fig. 9a**, and also reported in the main text as below:

> *"Most viral entities with a putative T7K homologue belonged to the Autographiviridae, the same family that the T7 phage belongs to, and had similar genome sizes to that of the T7 reference genome **(Fig. 5b, Supplementary Table 8)**. This group contained the experimentally validated phages from the BASEL collection (Bas64, Bas65, Bas66(T3K12), Bas68), which were also among the closest homologues of T7K. Interestingly, early proteins in nearly all Autographiviridae phages with T7K homologues were depleted in S/T/Y residues **(Extended Data Fig. 9a)**, suggesting that the kinase's promiscuity exerts positive selection on these genomes."*

- when performing the analysis of stoichiometry, I wonder if there could be some bias to quantify stoichiometry dependent on the abundance of the protein. I don't think this should be the case but the authors could check.

It is true that technically, more abundant proteins will have a larger dynamic range of differential stoichiometry log2 fold-changes, and also more chance of producing peptides from which to calculate ratios from. There is also a clear bias in the abundances of key protein groups, as we showed for DNA-binding proteins **(Rebuttal Fig. 1)**.

Our analyses of DNA- and RNA-binding protein intensities and the global proteome distribution visible in **Rebuttal Fig. 1** shows that the vast majority of the proteome is abundant enough to produce high stoichiometry hits.

The Rebuttal Figure 1 is included in the above responses to reviewer 1 comment #2 already.

Action:

- We thank the reviewer for their comment but prefer to leave the main text unedited, and note that we had already flagged the sensitivity as a caveat for the readers in the main text in the following lines "*Note that this analysis has false negatives, as robust ratios for lower abundance (phospho)peptides are harder to measure.*"

Referee #3 (Remarks to the Author):

This study investigates the T7 phage kinase and its role in disarming anti-phage defense systems. The authors perform a high quality phospho-proteome analysis, showing that T7K phosphorylates S/T/Y residues throughout the proteome. The analysis of peptides phosphorylated at a high stoichiometry shows a preference for nucleic acid binding proteins that is likely mediated by the SO domain of the kinase.

The authors then convincingly show that the T7K enables T7 to bypass the Retron-Eco9 and DarTG defense systems. They identify phosphorylation sites in proteins of these systems and show that phosphomimic mutants are indeed unable to provide defense. Finally, the authors screen the WT T7 and the T7K deletion mutant against a large collection of E. coli strains. This enabled the identification of a few strains that cannot be efficiently infected in the absence of T7K and one strain that the T7K mutant infects better than the WT. These results suggest that the T7K can inhibit a wide range of defense systems, and that strain IAI15 might contain a defense system triggered by the T7K. The identity of this system is not revealed here.

Altogether these results are consistent with a recent report investigating JSS1_004, an homologue of T7K, and which also described broad S/T/Y phosphorylation. The study by Bartolec et al. is quite complementary in several respects. The phosphoproteome analysis performed here is much deeper, revealing a more detailed picture of the T7K activity. The set of defense systems found to be inhibited is different, reinforcing the picture of a broad anti-defense mechanism. This study also opens multiple research avenues by uncovering multiple strains affected one way or another by the T7K. The description of this family of kinases is a significant finding in the field of bacterial immunity, and this study an important contribution. The impact of the present work would however be made higher by further improving the aspects that differentiate it from the published work on JSS1.

We thank the reviewer for their thoughtful and encouraging evaluation of our work. We are grateful for the recognition of the depth of our phosphoproteome analysis, the mechanistic insights into T7K-mediated disarming of defense systems, and the broader implications for bacterial immunity. We also appreciate the reviewer highlighting the complementarity of our study to the recent work on JSS1_004.

We agree that strengthening the aspects that distinguish our study from the JSS1 report will further enhance its impact. In response, we have included several new analyses as described at the top of this rebuttal letter that significantly extend and differentiate our study towards the conservation of the T7K function and the role of the shut-off domain in kinase activity. New experiments and analysis include phosphoproteomics studying diverse phages and their kinases, new phylogenetic and sequence/structure-based analyses of T7K homologues and new insights into key structural features of its active sites, and biochemical and functional experiments on the role of the shut-off domain in RNA-binding and kinase activity.

Main comments:

Figure 2D shows an increased odds ratio for nucleic acid binding proteins but not for DNA binding. Could you clarify why that is the case? How is this consistent with your hypothesis that DNA binding directs preferential phosphorylation? Figure 2b could also use a representation (maybe color code) to highlight which of the proteins are known to interact with DNA or RNA.

Whilst we originally found experimental evidence that DNA interacted with the shutoff domain in the in vitro refolding assay, our new experiments could not provide any evidence towards its RNA-binding properties (see our detailed response to **reviewer 1's comment #1**). A plausible explanation for enrichment of RNA-binding GO term hits could be transcription-translation coupling (colocalization of these proteins with DNA).

Regarding the odds ratio, RNA-binding proteins are higher abundance than DNA-binding **(Rebuttal Figure 1)** and so are theoretically more likely to be detected as high stoichiometry substrates in our phosphatase experiments. This would increase the proportion of RNA-binding proteins used as input for GO-term enrichment analysis, which would affect the odds ratios of RNA-binding and nucleic acid binding terms. Regardless of this bias we still have significant DNA-binding terms coming up, corresponding to many high stoichiometry DNA-binding proteins. This is now visualised in the **revised Fig. 2b** below.

It is worth mentioning that all defense systems substrates found by us as well as in the JSS1 study (7 in total) are DNA targeting/bound. Please see our response to **reviewer 1's comment #2** for more details.

The Rebuttal Figure 1 is included in the above responses to reviewer 1 comment #2 already.

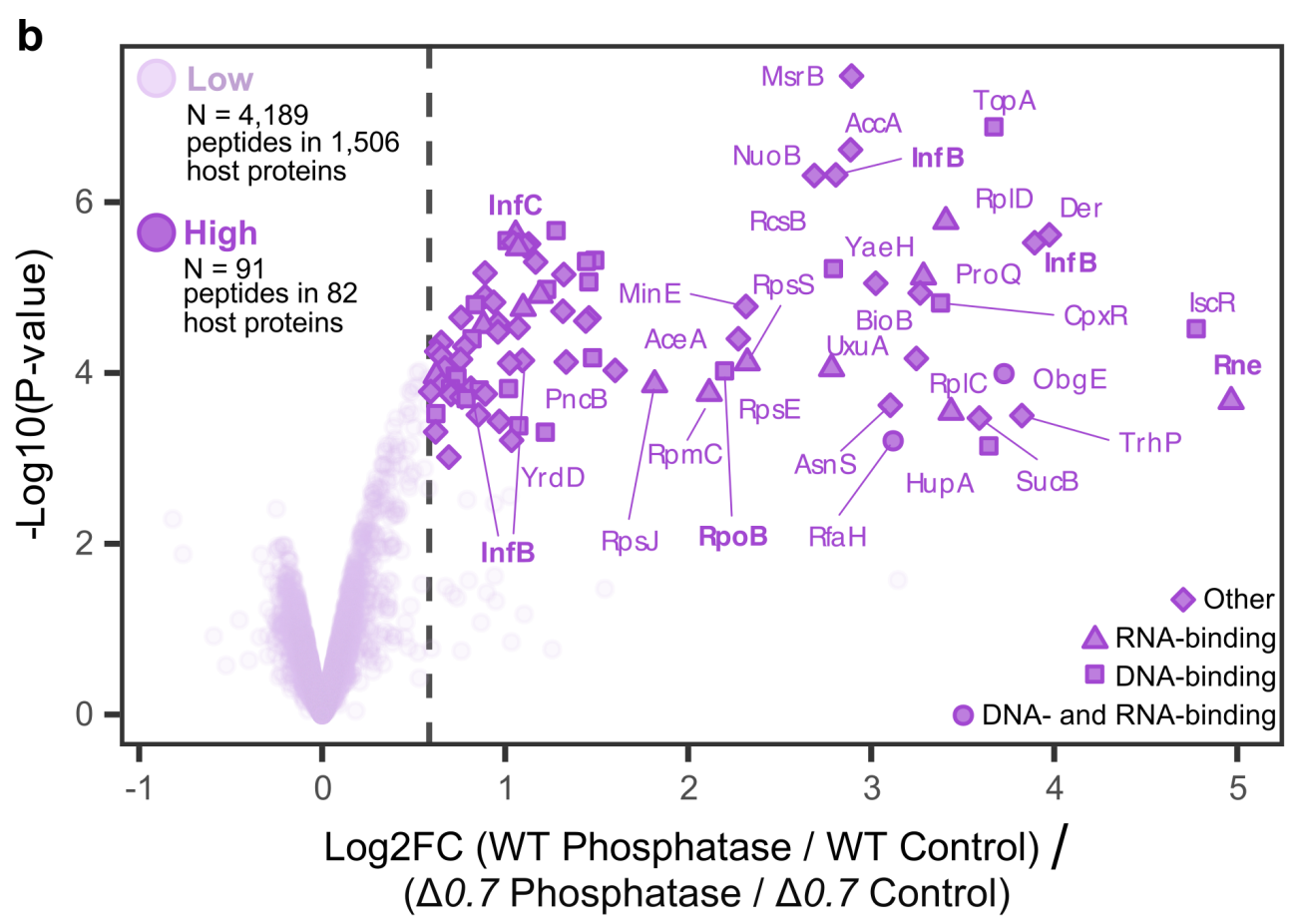

Revised Figure 2b: *Quantitative analysis of phosphorylation in wild-type vs Δ0.7 infected cells (T=10 minutes of infection, MOI=5, N=3 biological replicates) reveals high stoichiometry for specific host proteins – shown as volcano plots* ***(b)*** *and distributions of estimated stoichiometries (c). Only peptides uniquely mapping to host proteins, encompassing previously identified phosphosites from the time-course analysis (Fig. 1e), and with quality Phosphatase/Control ratios (see Methods), were assessed. Dark and light purple indicate peptides with a "High" (limma adjusted p-values<0.05, fold-change (FC)>1.5) and "Low" differential stoichiometry, respectively. The corresponding proteins are shown for peptides with high differential stoichiometry in b; bold font denotes previously described substrates. Shapes show membership of proteins to DNA- and/or RNA-binding GO-terms.*

Action:

- We now include a **revised Figure 2b** which includes the representation of values of peptides from proteins with DNA- and/or RNA-binding annotations as different shapes.

It would be interesting to test solubilization of the SO domain not just with DNA but also with RNA. Having some biochemical data to compare DNA vs. RNA binding affinity of the SO domain could also be helpful.

We have now introduced new experiments extending our refolding assay to consider RNA-binding properties, which revealed that the shutoff domain was not able to interact with RNA. Therefore, there is no direct evidence of RNA-binding. Please see the detailed description of these experiments in our response to **reviewer 1's comment #1**.

Action:

- We introduced a new experiment to expand upon the shutoff domain refolding assay to assess RNA-binding. We extend the existing **Fig. 2f** with this new data.
- We also introduce a new panel in the extended data describing control experiments to confirm integrity of total RNA preparations in the refolding experiment **(Extended Data Fig. 4c)**.

At the moment the role of the SO domain in mediating preferential phosphorylation is not directly shown by the authors. A direct demonstration of this would require performing the phospho-proteome analysis in a T7K mutant lacking the SO domain. Such a mutant would also be interesting to test against the anti-phage defense systems.

To address these comments, we initially planned experiments to directly compare phosphoproteome changes induced by infection of *E. coli* with either the wild-type phage T7 or a new mutant T7 missing the T7K shutoff domain (ΔSO). This revealed an unexpected additional function of the domain, where the loss of the shutoff domain appeared to generally dampen the overall extent of hyperphosphorylation **(new Fig. 2g-h)**. Through subsequent experiments, we discovered that the mutant has altered kinetics of kinase activity **(Fig. 2j)**, likely through earlier inactivation of kinase activity through auto-phosphorylation **(Fig. 2h)**. Nonetheless, we were able to clearly observe that the shutoff domain was indeed mediating the preference of T7K to highly modify nucleic-acid binding substrates, with the phosphorylation on substrates belonging to the high stoichiometry GO-terms much more extremely affected by its deletion **(Fig. 2i)**.

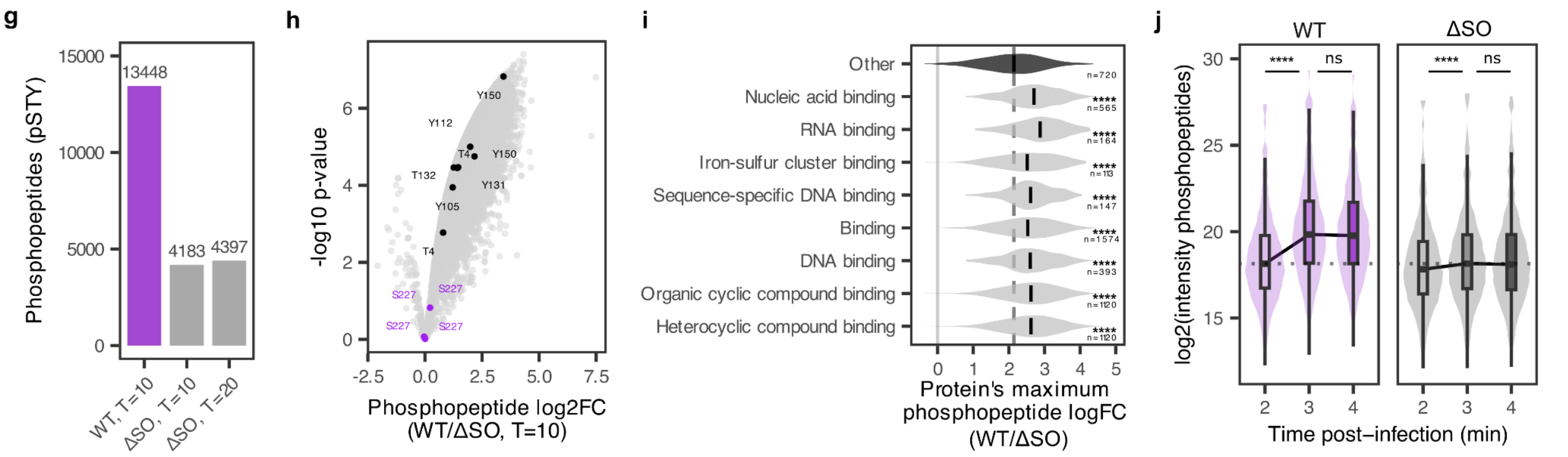

Revised Figure 2: (g) (g)** Loss of the SO domain reduces the scale of T7K-mediated hyperphosphorylation. Total number of unique serine (S), threonine (T) and tyrosine (Y) phosphopeptides identified from three biological replicates of wild-type and ΔSO infections using phosphoenrichment and label-free MS/MS analysis. MOI = multiplicity of infection. T = 10 or 20 minutes post-infection. **(h)** The loss of the SO domain generally dampens hyperphosphorylation, except for specific auto-sites. Limma analysis of quantitative TMT-based phosphoproteomics comparing 10 minutes of WT and ΔSO infection. N=3 independent biological replicates. Labelled sites are those within the T7K protein, with purple indicating previously reported auto-site from [9]. **(i)** The SO domain mediates higher modification rates of nucleic-binding proteins by T7K. The maximum phosphopeptide log2FC per protein, with proteins subsetted by their membership in the high stoichiometry substrates GO-terms identified from Fig. 2d. Crossbars show the median maximum phosphopeptide log2FC per protein in each group and the dotted line shows the median from phosphoproteins not belonging to these GO-terms ("Other"). Unpaired t-test analysis comparing each GO-term distribution to the "Other" distribution – * – p-value≤0.0001). **(j)** Loss of SO domain causes earlier inactivation of T7K activity. Summed TMT reporter ion intensities per phosphopeptide derived from a quantitative TMT-based phosphoproteomics monitoring 2, 3 and 4 minutes of WT and ΔSO infections. N = 3 independent biological replicates; unpaired t-test analysis – ns (non-significant), *** – p-value≤0.001, **** – p-value≤0.0001. The dotted line shows the median log2(summed intensity) for 2 minutes of WT infection. The distributions of summed reporter ion intensities in individual biological replicates of infections are shown in Extended Data Fig. 4e.*

Note: we now report the results of label-free phosphoproteomics on the new G76F mutant in **revised Fig. 1b**, where we also replace wild-type and Δ*0.7* datasets with newly acquired ones to match the T=10 minute post-infection sampling of other infections within the study.

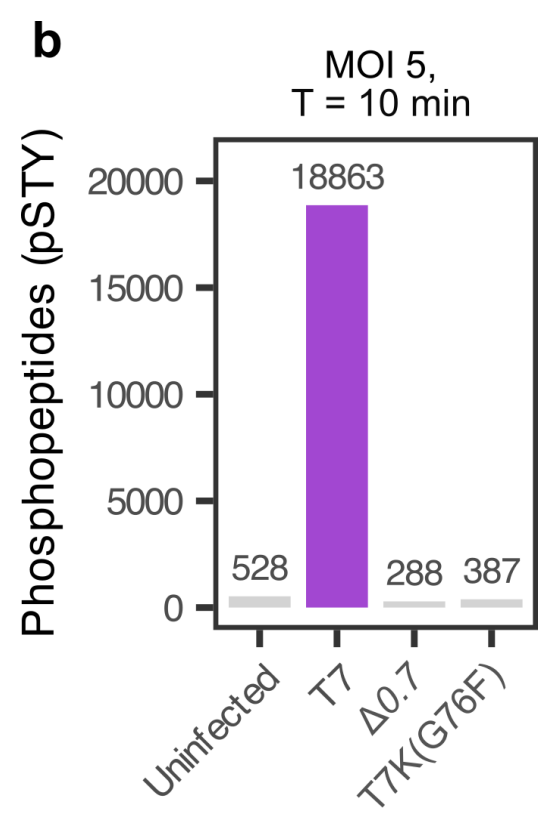

***Revised Figure 1b:** Infection by wild-type but not Δ0.7 or T7K(G76K) causes a massive phosphorylation of the E. coli proteome. Total number of unique serine (S), threonine (T) and tyrosine (Y) phosphopeptides identified from 3 biological replicates using phosphoenrichment and label-free MS/MS analysis. MOI = multiplicity of infection.*

We also performed several new infection assays using the two new mutants (catalytic-dead G76F, ΔSO) to dissect the effect of selective removal of either SO domain or catalytic activity of the kinase **(Extended Data Fig. 7)** which revealed a requirement for both modalities for efficient host takeover for the most sensitive strains and defense system. It also revealed that the SO domain has likely an independent anti-defense mechanism from the kinase.

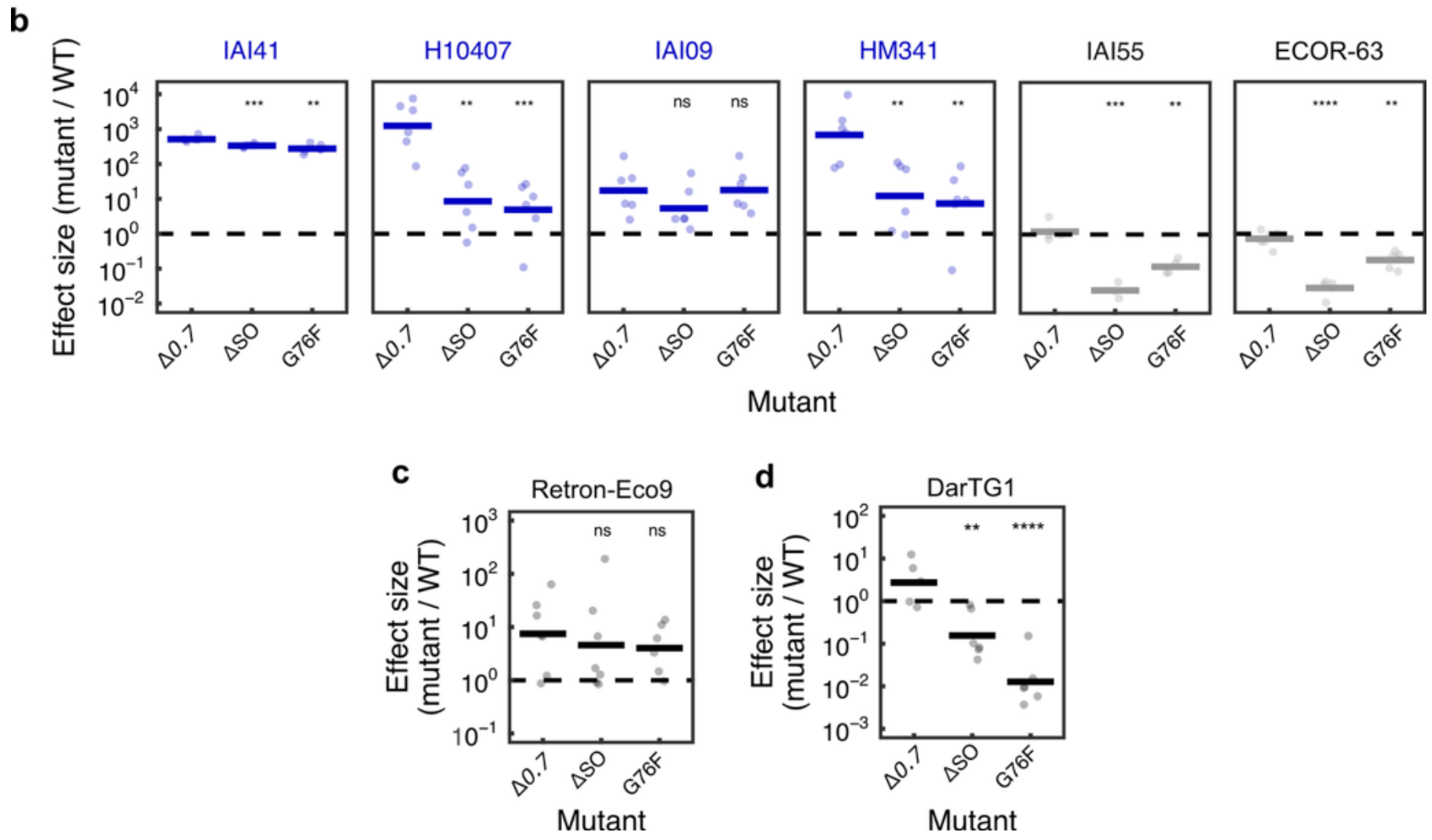

New Extended Data Figure 7: Infection assays on natural isolates and using partial T7K mutants. (b) *Mean log10(effect sizes) for selected natural isolates testing fitness of the expanded T7 mutant panel (Δ0.7, ΔSO and G76F catalytic dead) against WT T7. For these experiments, effect sizes were calculated using an expanded MOI range (5, 1, 0.5, 10^{-1} serially diluted 1:10 down to 10^{-8}). Blue strains are those determined to be highly T7K-sensitive, and black strains are control, i.e. T7K-neutral strains. Crossbars show the mean log10(effect sizes) and points show the estimated effect sizes from 4-6 biological replicates. Data was excluded when the effect size could not be quantified (as above). If the highest MOI did not lyse the population (mutants for IAI41), the effect size was estimated assuming that a 10-fold higher MOI would lyse the population. If the lowest MOI did not allow defense against a specific phage, the effect size was estimated assuming that a 10-fold lower MOI would allow defense against T7. The results of unpaired t-tests comparing log10(effect sizes) from partial mutants (ΔSO and G76F) to the full deletion (Δ0.7) are shown, with ns meaning non-significant (p-value > 0.05), ** meaning p-value ≤ 0.01, *** meaning p-value ≤ 0.001 and **** meaning p-value ≤ 0.0001. Effect sizes were estimated from the normalized AUC curves shown in Supplementary Fig. 4a.* ***(c)*** *Same as for panel b but showing the results of infection assays on BW25113 expressing Retron-Eco9 or* ***(d)*** *DarTG1. Effect sizes were estimated from the normalized AUC curves shown in Supplementary Fig. 4b for Retron-Eco9 and 4c for DarTG1.*

Action:

- We report on our new experiments investigating the role of the shutoff domain in regulating T7K specificity and kinase activity in new panels in Fig. 2 **(Fig. 2g-j)**, and in the following extension of the results under existing heading "*The T7 kinase gains specificity to nucleic acid-binding substrates via its C-terminal domain*", included verbatim below:

> *"To probe the function of the SO domain further, we quantified the phosphoproteome upon infection with a T7 phage carrying a truncated T7K (ΔSO). Deletion of the SO domain resulted in lower overall T7K-mediated phosphorylation (~31% of the phosphorylated peptides detected compared to WT) (Fig. 2g, Extended Data Fig. 4d, Supplementary Table 4). This was the case despite sampling at a late point in infection (10 minutes), when T7K activity has long been autoinhibited (Extended Data Fig. 2c). We also observed a substantial decrease in phosphorylation in the ΔSO mutant as compared to WT T7K by quantitative phosphoproteomics (Fig. 2h). Although this decrease was evident for nearly all proteins, GO-terms enriched in stoichiometric substrates for WT T7K (Fig. 2d) had an even more pronounced decrease in phosphorylation (Fig. 2i). This is consistent with the DNA-binding properties of the SO domain functioning to direct kinase preference to these substrates.*
>
> *We sought to better understand why the lack of the SO domain resulted in lower overall phosphorylation. We hypothesized that the kinase may be inhibited by autophosphorylation faster in the ΔSO mutant. Although we were once again unable to detect the autoinhibitory S216 site with TMT-based mass spectrometry, another prominent and adjacent auto-phosphorylation site, S227, could be detected and monitored via several peptides. Interestingly, all S227 phosphopeptides were equally well phosphorylated in WT and ΔSO infections (Fig. 2h, Supplementary Table 4), implying that the kinase is equally active in both situations. To get insights into the kinetics of phosphorylation of WT and ΔSO T7K, we quantitatively monitored the phosphoproteome earlier in infection (2, 3, 4 minutes post-infection). Although both phages produced similar levels of phosphorylation early on in infection (2 min), ΔSO-induced phosphorylation plateaued faster than its WT counterpart (Fig. 2j, Extended Data Fig. 4e).*
>
> *Together these results suggest a dual role for the SO domain in (1) directing a preference of the kinase to nucleic-acid binding substrates through its DNA-binding properties, and in (2) maintaining the kinase active for longer periods, likely through slowing down its catalytic autoinhibition."*

- We discuss these new findings on the shutoff domain in the discussion with the following sentences:

  To mitigate the dangers of such uncontrolled phosphorylation, T7 inactivates the kinase within 6 mins after infection via autophosphorylation. The timing of this inactivation seems to be regulated at least partially through the shutoff domain, likely through its interactions with the DNA which reduces the pool of free floating T7K molecules. To which degree the shutoff domain acts directly or via the kinase domain in defense inactivation remains to be fully elucidated.

- We include the extended infection assays in **Extended Data Fig. 7b-d** and report them as follows:

  "Uncovering a multitude of T7K kinase deletion phenotypes against both overexpressed defense systems and natural isolates prompted us to specifically dissect the effect of selective removal of either SO domain or catalytic activity of the kinase. We used an extended panel of T7K mutants (Δ0.7, catalytic-dead G76F, ΔSO) to dissect the functional importance of the two T7K domains (kinase, SO), focusing on the most T7K-sensitive strains (effect sizes ~100-1000 fold: IAI41, H10407, IAI09, HM-341) and two neutral strains (ECOR-63, IAI55). In two strains (IAI41 and IAI09) and for Retron-Eco9 loss of either the kinase activity or the SO domain phenocopied the full T7K deletion strains, whereas in two others (H10407 and HM-341) this led to partial loss of counter-defense (Extended Data Fig. 7b,c). These results suggest that the SO domain may also play a direct role, independent of the kinase domain, in defense inactivation of at least some of the defense systems (those in H10407 and HM-341). This role remains to be further elucidated, but may be linked with its reported role on RNA-polymerase inhibition. We also noticed that the two partial mutants (catalytic or SO domain) were fitter than the full T7K deletion when infecting neutral strains (ECOR-63, IAI55). Although we cannot fully explain this at this point (may be due to slightly different genetic backgrounds of the different phages – e.g. cmk marker in full deletion), this precluded us from rigorously assessing the role of individual domains in systems with small effect sizes, such as DarTG1 (Extended Data Fig. 7d)."

- We include new phosphoproteomics data on the G76F catalytic-dead mutant in the **revised Figure 1b**

T7 relies on nucleic acid-binding proteins for its cycle, including some of its own proteins such as the T7 RNAP. Are any of the T7 proteins phosphorylated at high stoichiometry? How does the solvent accessibility of, S, T and Y in phage proteins compare to that of E. coli proteins ? Do the proteins phosphorylated at high stoichiometry show better solvent accessibility?

Only one T7 protein was found to be highly stoichiometrically phosphorylated by T7K, which is gp4.7 (a protein with no known function). This information can be found in the existing **Supplementary Table 4**. To provide further nuance for readers, we modified the existing **Extended Fig. 3a** panel to only label the substrates in each infection which we consider to be high stoichiometry, independent of the differential stoichiometry analysis reported in **Fig. 2b**. Those are peptides with fold-changes>1.5 and adjusted p-values<0.05 in the phosphatase/control limma analysis. This highlights the fact that gp4.7 is uniquely highly stoichiometrically modified in wild-type T7 infection, and also an interesting finding that the host itself is likely phosphorylating the T7 proteins gp4.3 and gp3.5 to a very high stoichiometry.

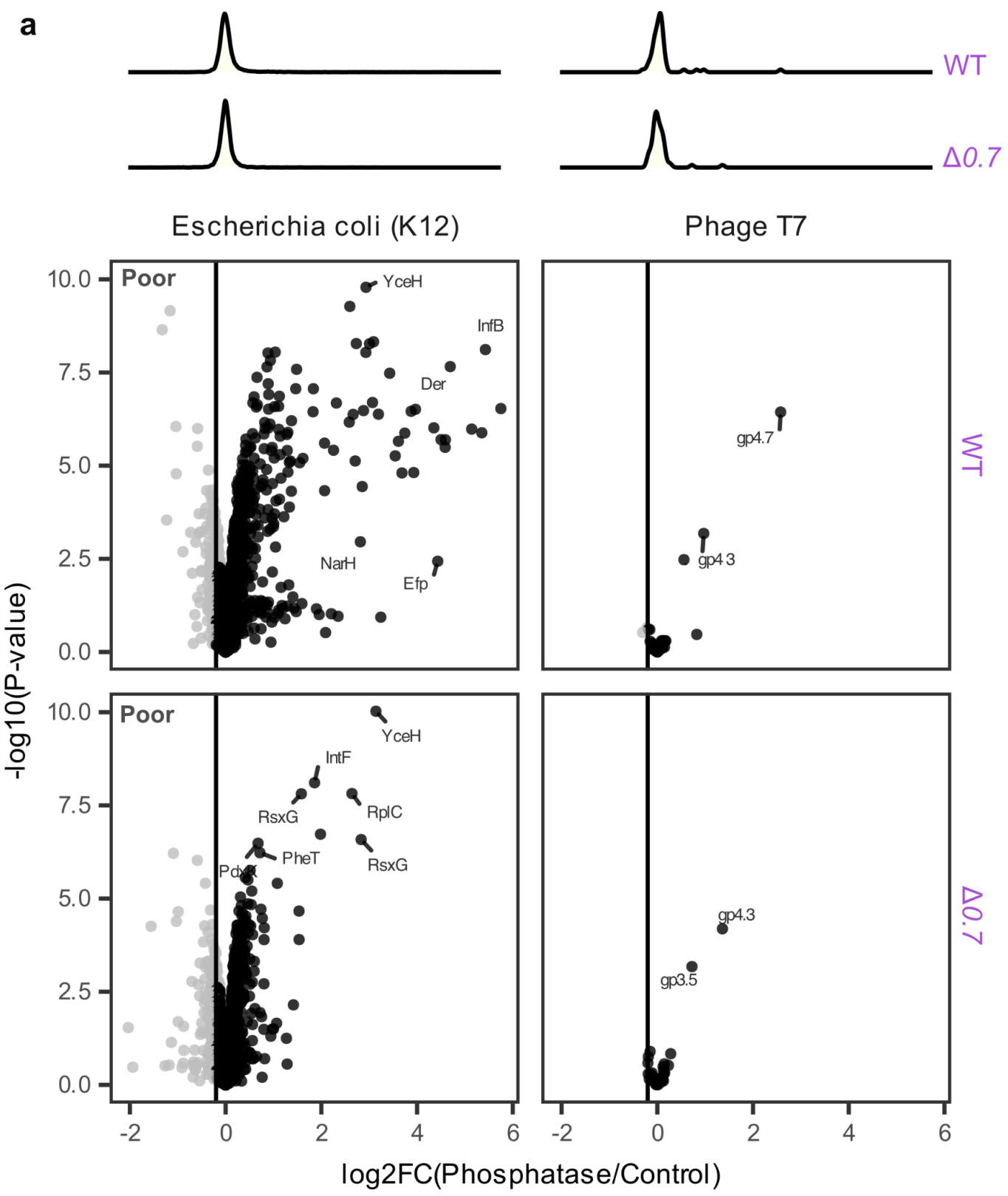

***Revised Extended Data Figure 3:** Estimating phosphorylation stoichiometries. **(a)** The Phosphatase/Control ratios of peptides across host and phage proteins considered for the estimation of phosphorylation stoichiometries (N = 4,371 peptides). These were (non-phosphorylated) peptides with residues previously identified to be phosphorylated in the TMT time-course phosphoproteomics dataset (Fig. 1e). Stoichiometries were estimated using three biological replicate samples of the T=10 minutes infection time-points, and as such only phosphosites on peptides representing proteins that were not found to significantly differ between WT T7 and Δ0.7 infection at 10 minutes in the protein expression analysis (Extended Data Fig. 4b) were considered. We excluded "Poor" Phosphatase/Control ratios (grey dots) from downstream differential stoichiometry analyses using limma (Fig. 2b) when the Phosphatase/Control ratio was either significantly down-regulated (limma, adjusted p-value < 0.05, log2 fold-change (FC) < 0) or the log2FC was low (logFC < -0.2, no significance requirement). The log2FC = -0.2 position is indicated. This represented the minority of ratios (see corresponding density histogram for each sample, top of the graph), but removed peptides that had clear evidence of confounding technical factors (as the addition of phosphatase should not result in a decrease in signal, in theory). Both ratios were categorised as poor if they were found to be poor in at least one of the conditions (wild-type T7 or Δ0.7). Several peptides which are labelled with the name of the protein they belong to, namely those that would be considered significantly high stoichiometries within their respective (adjusted p-value < 0.05, fold-changes (FC) > 1.5).*

Next, as requested by the reviewer, we sought to plot the host proteome's residue solvent accessibility values alongside the values for early, middle and late T7 proteins as shown in **Fig. 1h (Rebuttal Fig. 2a)**. However, host proteins have considerably skewed solvent accessibilities due to the fact they encode much larger proteins, and so a large portion of their residues are buried **(Rebuttal Fig. 2b).** Given the fact that there is no significant difference in protein lengths between early, middle and late phage proteins **(Rebuttal Fig. 2c)**, we propose to leave the existing **Fig. 2h** as is (that is, only showing T7 proteins sub-setted by phage gene class) to preserve clarity and flow of the manuscript.

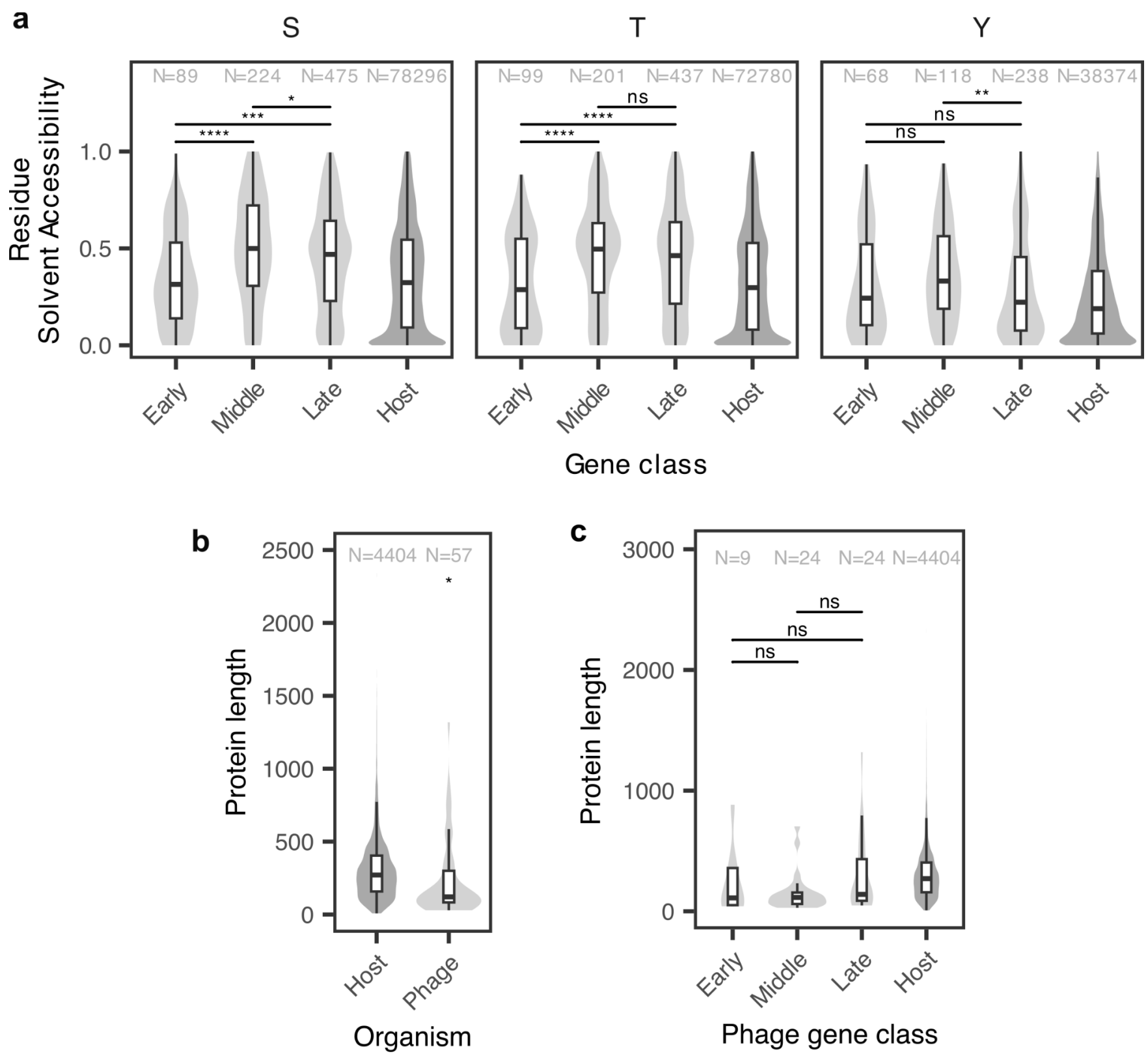

Rebuttal Figure 2: Host proteins have lower residue surface accessibilities than phage proteins as they are larger. (a) *Adapted Fig 1h with an additional distribution shown for host proteins.* ***(b)*** *Protein length (number of amino acids) in host and phage proteins.* ***(c)*** *Protein length in host proteome, or in phage proteome stratified by T7 gene class. For b and c, N= shows the number of proteins in each distribution and statistical significance is shown as * meaning p<0.05 and ns = non-significant, resulting from an unpaired t-test.*

Finally, we investigated whether there was any relationship between stoichiometry status (low, high) and surface accessibility. This appeared to be the case, with the high stoichiometry sites in proteins that had at least one high stoichiometry site being considerably more exposed than both other low stoichiometry sites in same protein or sites on low stoichiometry proteins **(new Extended Data Fig. 3b).**

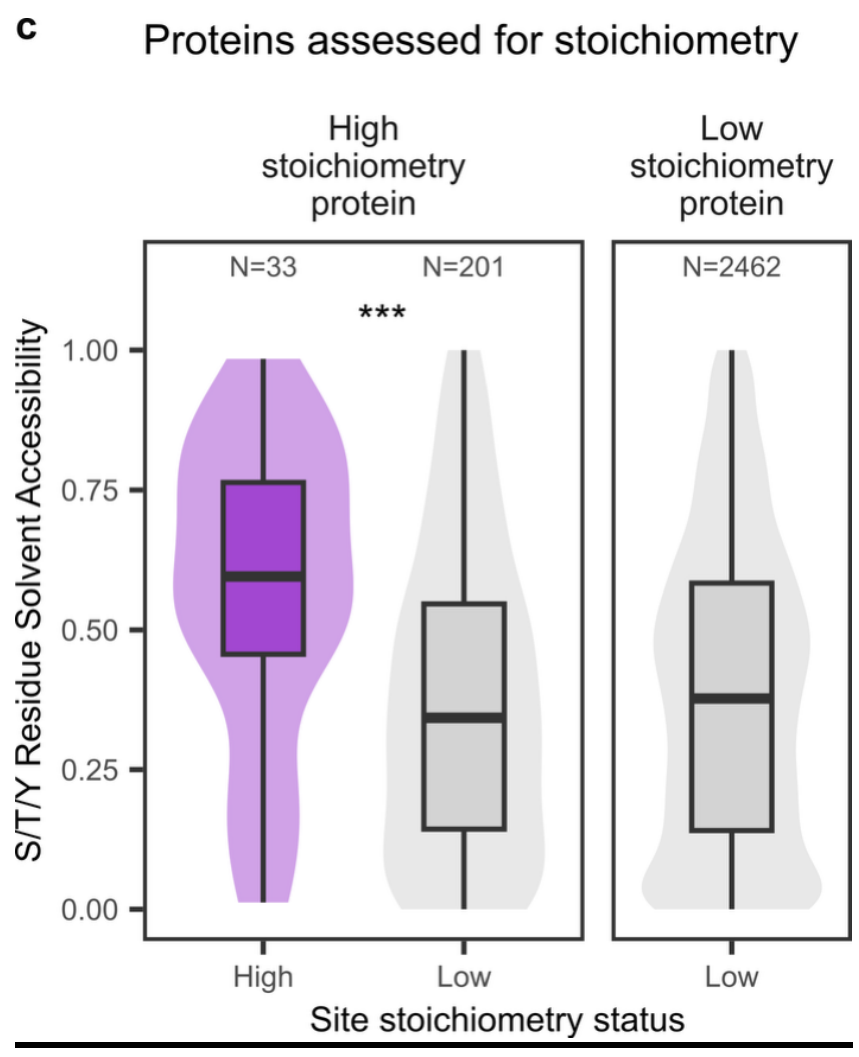

New Extended Data Figure 3b: *High stoichiometry sites are more surface accessible within protein structures. The residue surface accessibility values for S/T/Y residues were calculated from AlphaFold predictions for phosphoproteins that had quality phosphatase ratios calculated and used in Fig. 2b. Only proteins with peptide sequences distinctly encompassing a single phosphosite were considered.*

Action:

- To aid the readers in appreciating the high stoichiometry phage substrates, we now include a revised **Extended Data Figure 3a** to include distinction between phosphatase ratios in host and phage proteins

- We decided not include the distribution of host protein residue solvent accessibilities to aid readability.

- We add the new **Extended Data Figure 3b** to show the bias in higher solvent accessibility for high stoichiometry phosphosites, and introduce the result with the following sentence:

*"We then identified peptides with significantly higher Phosphatase/Control ratios in the WT over the Δ0.7 T7 infection (limma, adjusted p-value < 0.05 and fold-change > 1.5) **(Fig. 2b)**, which pointed to highly stoichiometric events **(Supplementary Table 4)**. Peptides belonging to 82 host proteins, including 4/11 known substrates (bold), exhibited highly stoichiometric phosphorylation at accessible residues **(Extended Data Fig. 3b)**."*

The authors discuss RcsB in the context of defense against phage infection mediated by the colonic acid capsule, but I struggle to understand how T7 would benefit from inactivating RcsB when it is already inside the cell? Could the authors clarify their thinking?

Colanic acid can help neighboring (uninfected) cells from being infected, as it is secreted and not attached to the cell surface, being a common good of a bacterial microcolony. Progeny could get trapped in this after release from an infected cell. Hence the new progeny would have better chances to infect neighboring cells if colanic production from an infected cell was halted.

Colanic acid capsules and resulting mucoidy[10] are known to directly influence phage infectivity by providing a physical barrier against adsorption. For example in T7-resistant *E. coli* strains prevent efficient adsorption by overproducing colanic acid, and *E. coli* phage PBECO4 produces the ORF547 colanic-acid degrading protein (in infected cells) to enable phage re-entry[11].

Therefore, we posit that T7K-mediated inactivation of biofilm synthesis would function to aid subsequent rounds of infection of T7 progeny in adjacent cells, as these phages would normally be subjected to increased difficulty in adsorption.

Action:

- We are not able to make any clear mechanistic statements on RcsB T7K-mediated phosphoregulation beyond our observations linking non-canonical RcsB phosphorylation to down-regulation of *wza* operon components. Considering this is a minor finding, we do not wish to speculate more and therefore would prefer not to expand upon this finding in our revised main text.

References

1. Jiang, S. *et al.* A widespread phage-encoded kinase enables evasion of multiple host antiphage defence systems. *Nat. Microbiol.* **9**, 3226–3239 (2024).

2. He, Y. *et al.* Mechanistic insights into JSS1_004-mediated antagonism of the DndBCDE-FGH restriction system and engineering applications. *MBio* **16**, e0138625 (2025).

3. Maffei, E. *et al.* Systematic exploration of Escherichia coli phage-host interactions with the BASEL phage collection. *PLoS Biol.* **19**, e3001424 (2021).

4. Castro-Roa, D. *et al.* The Fic protein Doc uses an inverted substrate to phosphorylate and inactivate EF-Tu. *Nat. Chem. Biol.* **9**, 811–817 (2013).

5. Arter, C., Trask, L., Ward, S., Yeoh, S. & Bayliss, R. Structural features of the protein kinase domain and targeted binding by small-molecule inhibitors. *J. Biol. Chem.* **298**, 102247 (2022).

6. Perfilyev, A. *et al.* Large-scale analysis of bacterial genomes reveals thousands of lytic phages. *Nat. Microbiol.* **11**, 42–52 (2026).

7. Peters, J. M. *et al.* A comprehensive, CRISPR-based functional analysis of essential genes in bacteria. *Cell* **165**, 1493–1506 (2016).

8. Mateus, A. *et al.* The functional proteome landscape of Escherichia coli. *Nature* **588**, 473–478 (2020).

9. Gone, S. & Nicholson, A. W. Bacteriophage T7 protein kinase: Site of inhibitory autophosphorylation, and use of dephosphorylated enzyme for efficient modification of protein in vitro. *Protein Expr. Purif.* **85**, 218–223 (2012).

10. Piché, L. C. *et al.* Evolutionary responses of Escherichia coli to phage pressure: insights into mucoidy and colanic acid overexpression. *BMC Genomics* **26**, 448 (2025).

11. Kim, M. S. *et al.* Phage-encoded colanic acid-degrading enzyme permits lytic phage infection of a capsule-forming resistant mutant Escherichia coli strain. *Appl. Environ. Microbiol.* **81**, 900–909 (2015).
